# A multicenter, randomized, double-blind, placebo-controlled phase 3 study of Socazolimab or placebo combined with carboplatin and etoposide in the first-line treatment of extensive-stage small cell lung cancer

**DOI:** 10.1038/s41392-024-02115-5

**Published:** 2025-01-13

**Authors:** Zhiwei Chen, Jianhua Chen, Dingzhi Huang, Wei Zhang, Lin Wu, Tienan Yi, Qiming Wang, Liang Han, Liping Tan, Yinyin Li, Zhihong Zhang, Na Li, Jie li, Tongmei Zhang, Ying Hu, Hongmei Sun, Youhua Wu, Zhiyong He, Runxiang Yang, Peng Cheng, Xingya Li, Jianhua Shi, Guohua Yu, Daiyuan Ma, Benjamin Xiaoyi Li, Xiangrong Dai, Michael Wong, Yujie Li, Xiaohui Yu, Shun Lu, for the Socazolimab Study Group, Zhiwei Chen, Jianhua Chen, Dingzhi Huang, Wei Zhang, Lin Wu, Tienan Yi, Qiming Wang, Liang Han, Liping Tan, Yinyin Li, Zhihong Zhang, Na Li, Jie li, Tongmei Zhang, Ying Hu, Hongmei Sun, Youhua Wu, Zhiyong He, Runxiang Yang, Peng Cheng, Xingya Li, Jianhua Shi, Guohua Yu, Daiyuan Ma, Shun Lu, Benjamin Xiaoyi Li, Xiangrong Dai, Michael Wong, Yujie Li, Xiaohui Yu

**Affiliations:** 1 Department of Medical Oncology, Shanghai Chest Hospital, Shanghai Jiao Tong University School of Medicine, Shanghai, China; 2 Department of Medical Oncology, Hunan Cancer Hospital, Changsha, China; 3 Department of Medical Oncology, Tianjin Tumor Hospital, Tianjin, China; 4 Department of Respiratory Medicine, First Affiliated Hospital of Nanchang University, Nanchang, China; 5 Department of Medical Oncology, Xiangyang Central Hospital, Xiangyang, China; 6 Department of Medical Oncology, The Affiliated Cancer Hospital of Zhengzhou University & Henan Cancer Hospital, Zhengzhou, China; 7 Department of Medical Oncology, Xuzhou Central Hospital, Xuzhou, China; 8 Department of Medical Oncology, Guangxi Medical University cancer Hospital, Nanning, China; 9 Department of Medical Oncology, Shenyang Tenth People’s Hospital, Shenyang, China; 10 Department of Respiratory Medicine, Anhui Provincial Cancer Hospital, Hefei, China; 11 Department of Medical Oncology, Suining Central Hospital, Suining, China; 12 Department of Respiratory Medicine, First Affiliated Hospital of Gannan Medical University, Ganzhou, China; 13 Department of Medical Oncology, Beijing Chest Hospital Affiliated to Capital Medical University, Beijing, China; 14 Department of Medical Oncology, Jiamusi Cancer Hospital, Jiamusi, China; 15 Department of Medical Oncology, First Affiliated Hospital of University of South China, Hengyang, China; 16 Department of Medical Oncology, Fujian Cancer Hospital, Fuzhou, China; 17 Department of Medical Oncology, Yunnan Cancer Hospital, Kunming, China; 18 Department of Medical Oncology, First Affiliated Hospital of Nanyang Medical College, Nanyang, China; 19 Department of Medical Oncology, First Affiliated Hospital of Zhengzhou University, Zhengzhou, China; 20 Department of Medical Oncology, Linyi Cancer Hospital, Linyi, China; 21 Department of Medical Oncology, Weifang People’s Hospital, Weifang, China; 22 Department of Medical Oncology, Affiliated Hospital of North Sichuan Medical College, Nanchong, China; 23 Zhaoke (Guangzhou) Oncology Pharmaceuticals, Guangzhou, China

**Keywords:** Lung cancer, Drug development

## Abstract

This is a randomized, double-blind, placebo-controlled phase 3 clinical trial (ClinicalTrials.gov, NCT04878016) conducted in 54 hospitals in China. Adults who were histologically diagnosed and never treated for extensive-stage small cell lung cancer (ES-SCLC) were enrolled. Eligible Patients were randomly assigned (1:1) to receive four cycles (21 days as one cycle) of intravenous carboplatin (area under the curve of 5 mg/mL per min, day 1 of each cycle) and etoposide (100 mg/m² of body-surface area, on days 1–3 of each cycle) with either socazolimab (5 mg/kg, day 1 of each cycle) or matching placebo, following maintenance therapy with socazolimab or placebo. From July 15, 2021, to May 12, 2022, 498 eligible patients were randomly assigned to receive socazolimab (250 patients) or placebo (248 patients) combined with chemotherapy. As of October 13, 2023, patients treated with socazolimab presented significant overall survival (OS) benefit (13.90 months) compared with the placebo plus EC group (11.58 months) (hazard ratio for death, 0.799; 95% CI, 0.652–0.979; *p* = 0.0158). The median progression free survival (PFS) was 5.55 months in the socazolimab plus EC group, prolonging disease progression or death by nearly 1.2 months (5.55 months vs 4.37 months, hazard ratio for progression or death, 0.569; 95% CI, 0.457–0.708; *p* < 0.0001). 200 (80.3%) patients in the socazolimab plus EC group experienced ≥ grade 3 treatment-related adverse events and 187 (75.7%) patients occurred in the placebo plus EC group. Socazolimab combined with standard EC regimen chemotherapy for first-line treatment of ES-SCLC significantly prolonged overall survival and did not increase the safety risk of treatment.

## Introduction

Small cell lung cancer (SCLC) accounts for about 13% of lung cancer and is the most aggressive subtype,^1,2^ with a poor prognosis of the average 5-year overall survival (OS) rate is only 5%.^3^ SCLC has the characteristics of short tumor cell division time and rapid multiplication, which causes most patients to have metastasis by the time of initial diagnosis .For more than 30 years since the 1990s, platinum-based (combined with etoposide or irinotecan) has been the standard chemotherapy for ES-SCLC, and the survival time of chemotherapy is about 10 months.^4,5^ Although the mechanism is not yet clear, rapid resistance to chemotherapy leads to an unsustainable treatment response and rapid progression in SCLC. Etoposide plus cisplatin (EP) is the preferred regimen for patients with ES-SCLC. In recent years, carboplatin has also been commonly used instead of cisplatin in clinical practice, due to the comparable efficacy of carboplatin combined with etoposide (EC) and EP. Because carboplatin has lower emetic and renal toxicity, but carboplatin also cause allergic reactions and increase the risk of hematologic toxicity. Etoposide combined with platinum based drugs is generally used for 4 cycles in first-line treatment, with a maximum of 6 cycles. Longer chemotherapy cycles do not improve survival rates, and there are no better treatment options in second-line treatment.

Until the emergence of immunotherapy represented by immune checkpoint inhibitors (ICIs) in recent years, it has brought long-lost progress to the treatment of ES-SCLC. Immunotherapy targeting programmed cell death protein 1/ligand 1 (PD-1/PD-L1) or cytotoxic T lymphocyte antigen (CTLA)-4 is currently the two most critical immunotherapy sequencing methods. Blocking checkpoints can block the tumor immune evasion system, reactivate the function of T cell effectors, and enhance anti-tumor immunity, leading to cancer cell death. The anti-tumor therapeutic effect of chemotherapy drugs is achieved by inducing immune cell apoptosis, inhibiting regulatory T cells to restore T cell activity, and reducing the number of myeloid-derived suppressor cells, thereby achieving immune enhancement. Due to the fact that ICIs and chemotherapy act at different stages of tumor cell apoptosis, theoretically they have a synergistic anti-tumor effect. The feasibility of combination therapy has also been explored on ES-SCLC. with the success of PD-L1 inhibitors Atezolizumab and Durvalumab in Impower133 study and CASPIAN study,^6,7^ it is proved that PD-L1 monoclonal antibody combined with standard first-line chemotherapy can significantly prolong the survival time of patients with extensive SCLC. This result has been continuously verified by similar drugs in SCLC, and then Serplulimab has demonstrated for the first time that PD-1 monoclonal antibody can also prolong survival in ASTRUM-005 study,^8^ which is mainly in Chinese ES-SCLC patients. At the same time, CAPSTONE-1,^9^ EXTENTORCH and RATIONALE-312 studies carried out entirely in the Chinese patients have once again confirmed that PD-L1 or PD-1 monoclonal antibody have similar effects.^10,11^ Through subgroup analysis of the above studies, we found that different anti-PD-1/L1 monoclonal antibodies combined with the same chemotherapy drugs have different benefits for the same population. Taking Atezolizumab and Durvalumab, which were first approved by NMPA, as an example. Both subgroups of the study showed that female patients had better survival benefits than male patients, but the Atezolizumab treatment group had better efficacy in the population aged ≥65 years, while the Durvalumab treatment group showed better efficacy in the population aged <65 years. Similarly, two large Phase III studies conducted in China showed that male patients had better life benefits in their subgroups. The Serplulimab treatment group showed better efficacy in patients with ECOG score 0, while the Adebrelimab treatment group showed better efficacy in patients with ECOG score 1. Currently, there is no clear and reliable explanation for the above results, and it cannot be ruled out that structural differences in the composition of PD-1/L1 may be the cause.

Socazolimab (formerly ZKAB001) is a fully human IgG1 monoclonal antibody that can only bind to PD-L1 protein in monkeys and humans. We explored pharmacokinetics and recommended phase 2 dose for advanced recurrent metastatic cervical cancer,^12^ adjuvant therapy for osteosarcoma^13^, and first-line treatment for urothelial carcinoma. The appropriate dosage for Phase 2 is 5 mg/kg/time, with a treatment cycle of 2–3 weeks. Based on the efficacy and safety results obtained in patients with recurrent and metastatic cervical cancer, the China National Medical Products Administration (NMPA) granted conditional approval of Socazolimab launch in December 2023. Prior to this study, a single-arm, phase 1b exploratory trial was completed.^14^ A total of 20 ES-SCLC patients who met the inclusion criteria were treated with socazolimab combined with EC. The study has preliminarily confirmed the safety of combination therapy and reported Median OS was 14.88 months .Preliminary clinical trials with small sample sizes had shown the anti-tumor activity of combination therapy, we conducted phase 3 clinical trials in China to further explore the efficacy and safety of socazolimab combined with EP for ES-SCLC in a large sample population.

## Results

### Demographic and baseline characteristics

**Fig. 1:**
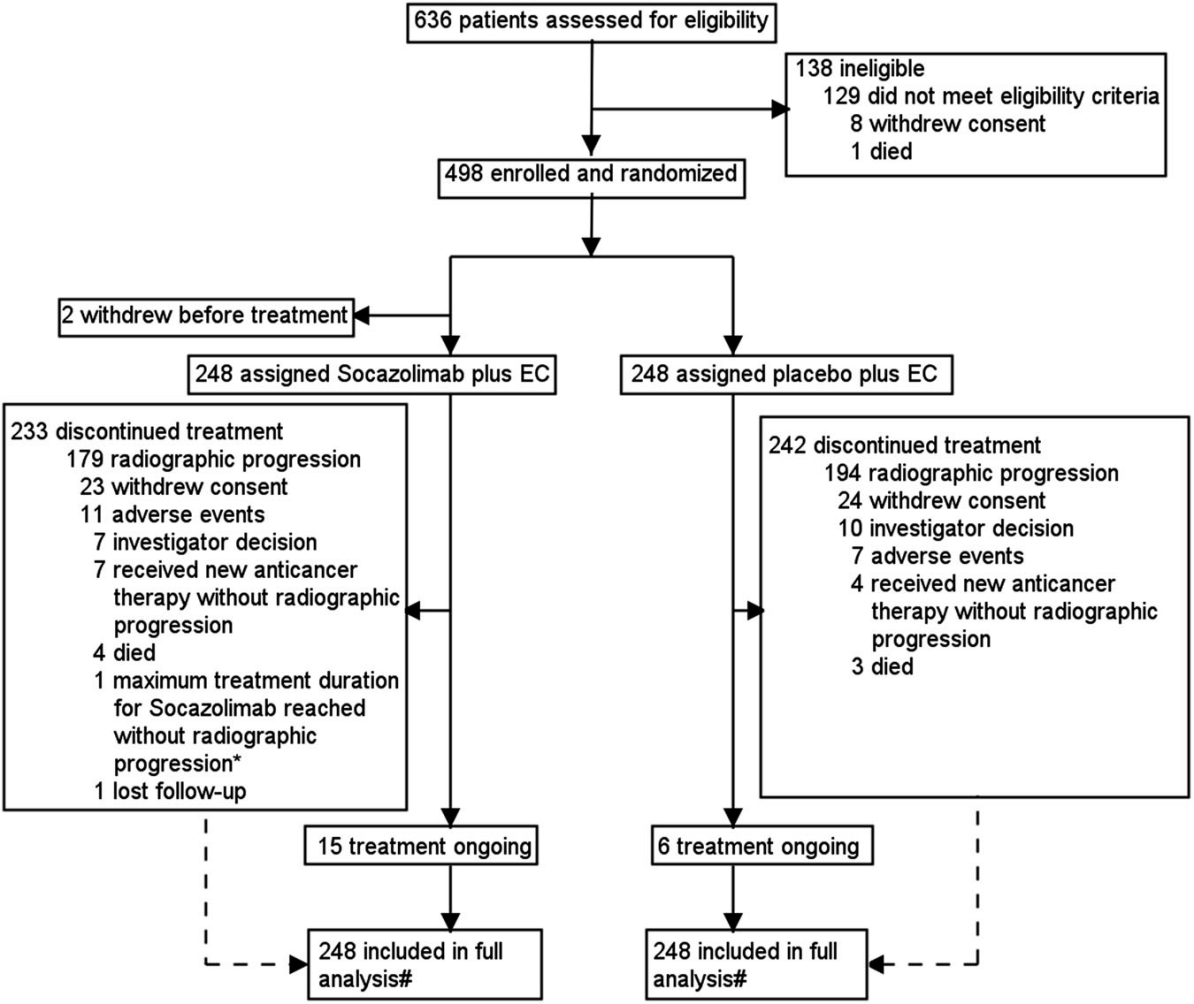
Trial Profile. The date of data cutoff was October 13, 2023. ^*^The maximum duration of treatment allowed for socazolimab or placebo was 2 years. One patient discontinued treatment due to reaching the maximum medication duration of 2 years. ^#^ full analysis: all Patients who received at least 1 dose of study treatment

From July 15, 2021, to May 12, 2022, 636 patients were screened in 54 hospitals in China. Finally, 498 patients were randomized and 496 of them had received at least one dose of socazolimab or placebo. There were 248 patients in the socazolimab plus EC group and 248 in the controlgroup. As October 13th, 2023, 15 patients in the socazolimab plus EC group and 6 patients in the control group had not yet completed treatment. The median duration of follow-up for OS was 13.86 months in the socazolimab plus EC group and 11.58 months in the placebo plus EC group. Two treatment groups had 233 patients (94.0%) and 242 patients (97.6%) who discontinued treatment, respectively the socazolimab plus EC groupthe placebo plus EC group (Fig. 1). The enlargement of tumor lesions or the emergence of new lesions, which cannot be effectively controlled, is the main reason for discontinuing research drug treatment. After study medication, 188 patients (75.8%) in the socazolimab plus EC group and 209 patients (84.3%) in the placebo plus EC group received subsequent anticancer therapy. The most frequent subsequent anticancer therapy was chemotherapy or chemotherapy combined with other therapies, with 149 patients (60.1%) in the socazolimab plus EC group and 161 patients (64.9%) in the placebo plus EC group. In addition, 59 (23.8%) and 54 (21.7%) patients in the socazolimab and placebo groups respectively used immunotherapy or immunotherapy combined with other therapies (Supplementary Table 1) .

The baseline demographic and disease characteristics of the patients included in both groups were balanced (Table 1), but median age of the socazolimab plus EC group (64.0 years) was slightly higher than that of the placebo plus EC group (61.0 years). In the full analysis set, the median age was 61.9 years (range 31–79) and most patients were men (411 [82.9%]), and had stage IV disease at baseline (449 [90.5%]), 17 (3.4%) patients had brain metastases, 122 (24.6%) patients had liver metastases, 409 (82.5%) patients with an ECOG performance status of one and 384 (77.4%) patients with PD-L1 tumor cells <1%.

**Table 1:** Demographic and baseline characteristics

	Socazolimab plus EC group *n* (%),(*N* = 248)	Placebo plus EC group *n* (%),(*N* = 248)
Age, (years)		
Median (range)	62.8 (38,79)	61.0 (31,79)
≥65	119 (48.0)	98 (39.5)
<65	129 (52.0)	150 (60.5)
Gender		
Male	206 (83.1)	205 (82.7)
Female	42 (16.9)	43 (17.3)
Disease stage		
III	27 (10.9)	20 (8.1)
IV	221 (89.1)	228 (91.9)
Brain metastases		
Yes	10 (4.0)	7 (2.8)
ECOG performance status		
0	44 (17.7)	43 (17.3)
1	204 (82.3)	205 (82.7)
PD-L1 tumor cells		
<1%	189 (76.2)	195 (78.6)
≥1%	53 (21.4)	50 (20.2)
Not evaluable or no data	6 (2.4)	3 (1.2)

Data are *n* (%) or median (range)

*ECOG* Eastern Cooperative Oncology Group

### Efficacy

**Fig. 2:**
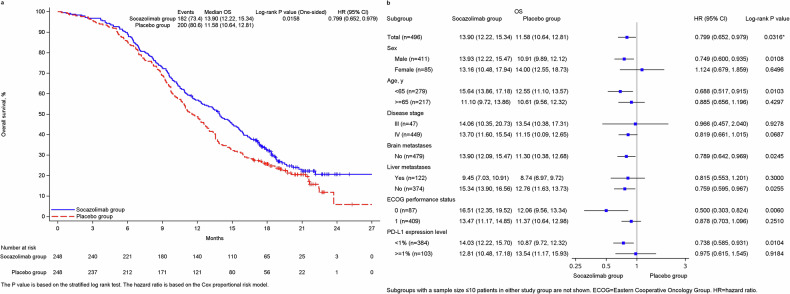
**a** Kaplan-Meier curve of overall survival. **b** Forest plot of subgroup analysis of overall survival Median OS follow-up time was estimated by the reverse Kaplan-Meier method with 95% Cis estimated using the Brookmeyer-Crowley method. The median duration of follow-up for overall survival was 20.47 months (IQR, 18.33–22.18 months) for the socazolimab plus EC group and 20.63 months (IQR, 19.78–21.49 months) for the placebo plus EC group. ^*^Calculated using a 2-sided stratified log-rank test

At final analysis, 182 patients (73.4%) in the socazolimab plus EC group and 200 patients (80.6%) in the placebo plus EC group had died. Compared with 48 patients (19.4%) in the placebo plus EC group who were still alive, 64 patients (25.8%) out of 66 patients (26.6%) in the socazolimab group were still alive, 2 others patients (0.8%) were lost to follow-up. The median OS was 13.90 m (95% CI, 12.22–15.34) in the socazolimab plus EC group compared with 11.58 m (95% CI, 10.64,12.81) in placebo group. The1-and 2-year survival rates were 56.8% (95% CI, 50.4–62.7) and 20.7% (95% CI, 14.8–27.3) in the socazolimab plus EC group and were 48.8% (95% CI, 42.4–54.8) and 5.9% (95% CI, 0.8–18.9) in the placebo plus EC group. Compared with the control group, the combination of socazolimab and EC group extended the OS of the subjects by more than 2 months and reduced the mortality rate by 20%. (HR, 0.799；95% CI, 0.652–0.979, one-sided *p* = 0.0158) (Fig. 2a). The analysis of OS results based on the pre-set subgroups of the trial shown in Fig. 2b.

**Fig. 3:**
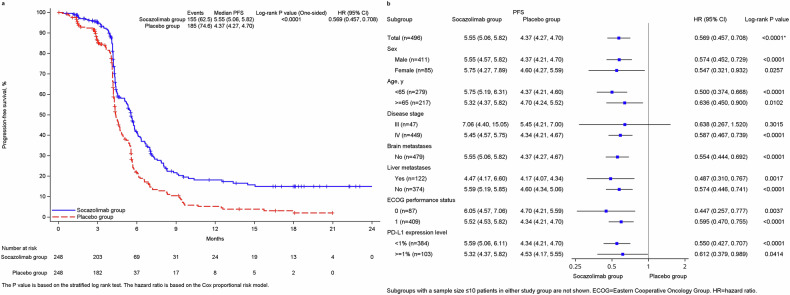
**a** Kaplan-Meier curve of progression free survival. **b** Forest plot of subgroup analysis of progression free survival. Data are from assessments by the independent radiology review committee using RECIST version 1.1. The median duration of follow-up for progression-free survival was 13.6months (IQR, 4.67–18.37 months) for the socazolimab plus EC group and 16.62 months (IQR, 5.09–18.07 months) for the placebo plus EC group. ^*^Calculated using a 2-sided stratified log-rank test

155 patients (62.5%) and 185 patients (74.6%) had disease progression or died at cutoff date. The median progression free survival (PFS) assessed by IRC was 5.55 m (95% CI, 5.06–5.82) in the socazolimab plus EC group compared with 4.37 m (95% CI, 4.27–4.70) in the placebo plus EC group with an hazard ratio of 0.569 (95% CI, 0.457–0.708, *p* < 0.0001)(Fig. 3a). Progression results of prespecified subgroups are shown in Fig. 3b. 176 patients (75.5%) in the socazolimab plus EC group and 160 patients (68.1%) in the placebo plus EC group had confirmed partial response of disease, with no complete response in both groups. The objective response rate of the socazolimab plus EC group was 75.5% (95% CI, 69.5–80.9), and 68.1% (95% CI, 61.7–74.0) in the placebo plus EC group. The duration of disease response (DoR) for patients in the combination therapy group with socazolimab and chemotherapy could reached 4.44 m (95% CI, 4.11–5.32), while the DoR for patients treated with placebo and chemotherapy is only 3.45 m (95% CI, 3.06–4.11). The 6-month and 12-month response rates in the socazolimab plus EC group were 33.3% and 20.5%, respectively, and those in the placebo plus EC group were 18.7% and 5.5%, respectively. The results of the investigators’ assessment were similar to those of the IRC, as shown in Table 2.

**Table 2:** Secondary outcomes of tumor response assessed by the independent radiology committee and investigators

	Socazolimab plus EC group (*N* = 248)	Placebo plus EC group (*N* = 248)
Secondary outcomes assessed by IRC		
Progression free survival (PFS)		
Events, *n* (%)	155 (62.5)	185 (74.6)
Median (95% CI) (months)	5.55 (5.06–5.82)	4.37 (4.27–4.70)
Hazard ratio (95% CI) and one-sided p^a^		0.569 (0.457–0.708), <0.0001
6 months PFS % (95%CI)	41.3 (34.2–48.2)	21.9 (16.2–28.1)
12 months PFS% (95%CI)	18.2 (12.6–24.5)	5.2 (2.5–9.4)
Objective response rate (ORR), *n* (%) (95% CI) ^b^	176 (75.5) (69.5–80.9)	160 (68.1) (61.7–74.0)
Complete response (CR) *n* (%)	0	0
Partial response (PR) *n* (%)	176 (75.5)	160 (68.1)
Stable disease (SD) *n* (%)	48 (20.6)	54 (23.0)
Duration of response (DoR)		
Median (95% CI) (months)	4.44 (4.11–5.32)	3.45 (3.06–4.11)
DoR ≥ 6 months (%)	33.3	18.7
DoR ≥ 12 months (%)	20.5	5.5
Secondary outcomes assessed by investigators		
Progression free survival (PFS)		
Events, *n* (%)	199 (80.2)	214 (86.3)
Median (95% CI) (months)	5.32 (4.44–5.65)	4.30 (4.17–4.40)
Hazard ratio (95% CI) and one-sided p^a^		0.653 (0.536–0.795), <0.0001
6 months PFS % (95%CI)	33.3 (27.0–39.7)	21.2 (16.0–26.8)
12 months PFS% (95%CI)	12.2 (8.2–17.1)	3.1 (1.3–6.2)
Objective response rate (ORR), *n* (%) (95% CI) ^b^	165 (70.5) (64.2–76.3)	154 (65.3) (58.8–71.3)
Complete response (CR) *n* (%)	0	0
Partial response (PR) *n* (%)	165 (70.5)	154 (65.3)
Stable disease (SD) *n* (%)	52 (22.2)	53 (22.5)
Duration of response (DoR)		
Median (95% CI) (months)	4.24 (4.11–4.63)	3.88 (3.02–4.11)
DoR ≥ 6 months (%)	42.0	22.9
DoR ≥ 12 months (%)	19.8	3.9

^a^The *P* value is based on the stratified log rank test. The hazard ratio is based on the CoX proportional risk model

^b^Based on the evaluable patients in the full analysis set

### Safety

At the data cutoff, median number of cycles was 8 (range, 1–32 cycles) in the socazolimab plus EC group and 6 (range, 1–36 cycles) in the placebo plus EC group. One patient who was randomly assigned into the placebo plus EC group was analyzed in the socazolimab plus EC group in the safety set because he had once mistakenly treated with socazolimab instead of placebo. 246 (98.8%) patients in the socazolimab plus EC group and 243 (98.4%) patients in the placebo plus EC group had treatment-related adverse events (TRAE, defined as adverse events related to socazolimab/placebo or chemotherapy), including two groups of 200 (80.3%) and 187 (75.7%), who experienced ≥grade 3AEs.the socazolimab plus EC group the placebo plus EC group (Table 3).

**Table 3:** Treatment related adverse events in safety set

	Any grade^b^		Grade ≥ 3^c^	
	Socazolimab (*n* = 249)	Placebo (*n* = 247)	Socazolimab (*n* = 249)	Placebo (*n* = 247)
Any treatment related adverse event^a^	246 (98.8)	243 (98.4)	200 (80.3)	187 (75.7)
Decreased neutrophil count	221 (88.8)	224 (90.7)	172 (69.1)	165 (66.8)
leukocytopenia	221 (88.8)	222 (89.9)	110 (44.2)	84 (34.0)
Anemia	212 (85.1)	205 (83.0)	58 (23.3)	51 (20.6)
Decreased platelet count	184 (73.9)	163 (66.0)	84 (33.7)	69 (27.9)
Nausea	79 (31.7)	70 (28.3)	3 (1.2)	4 (1.6)
Increased alanine aminotransferase	61 (24.5)	57 (23.1)	3 (1.2)	0
Increased aspartate aminotransferase	57 (22.9)	44 (17.8)	2 (0.8)	1 (0.4)
Vomiting	45 (18.1)	42 (17.0)	3 (1.2)	2 (0.8)
Constipation	34 (13.7)	38 (15.4)	0	0
Hyponatremia	43 (17.3)	31 (12.6)	12 (4.8)	12 (4.9)
Decreased appetite	43 (17.3)	43 (17.4)	1 (0.4)	0
Asthenia	33 (13.3)	22 (8.9)	2 (0.8)	0
Decreased body weight	32 (12.9)	21 (8.5)	0	0
Hypoalbuminemia	28 (11.2)	23 (9.3)	0	0
Increased alkaline phosphatase	26 (10.4)	17 (6.9)	3 (1.2)	0
Hypothyroidism	25 (10.0)	8 (3.2)	0	0
Decreased lymphocyte count	21 (8.4)	18 (7.3)	8 (3.2)	6 (2.4)
Hypokalemia	18 (7.2)	25 (10.1)	3 (1.2)	7 (2.8)
Infectious pneumonia	15 (6.0)	9 (3.6)	11 (4.4)	6 (2.4)

Data are *n* (%)

^a^Adverse events related to socazolimab/placebo, carboplatin or etoposide. Adverse events were graded according to version 5.0 of the National Cancer Institute Common Terminology Criteria for Adverse Events.b. Adverse events related to socazolimab or placebo

^b^Occurred in 10% or greater of patients in either group

^c^Occurred in 2% or greater of patients in either group

The most common TRAEs ≥ grade 3 in socazolimab group and placebo group were mainly hematological toxicities, including decreased neutrophil count (69.1% and 66.8%, respectively), leukocytopenia (44.2% and. 34.0%, respectively), decreased platelet count (33.7% and 27.9%, respectively) and anemia (23.3% and 20.6%, respectively) (Table 3). The TRAEs that led to termination of treatment in the socazolimab plus EC group and placebo group occurred in 12 (4.8%) and 7 (2.8%) patients, respectively (Supplementary Table 2). The death caused of TRAE were reported in 3 patients (1.2%, 1 patient of acute pancreatitis complicated with infectious pneumonia and 2 patients of unknown cause of death) in the socazolimab plus EC group and 4 patients (1.6%, 1 patient of heart failure complicated with infectious pneumonia, 1 patient of ventricular fibrillation and 2 patients of unknown cause of death) in the placebo plus EC group.

Immune-related adverse events (irAE) were reported in 19.3% patients in the socazolimab plus EC group and 9.7% patients in the placebo plus EC group. The most common irAE in the socazolimab plus EC group were hypothyroidism (3.6%), hyperthyroidism (2.8%) and elevated alanine aminotransferase (2.8%); and in the placebo plus EC group were alanine aminotransferase increased (2.8%), aspartate aminotransferase increased (1.2%). Grade 3 or above irAE occurred in 6.0% patients in the socazolimab plus EC group and 0.8% patients in the placebo plus EC group, with alanine aminotransferase increased (1.2%) in the socazolimab plus EC group and pneumonia (0.8%) most commonly seen in the placebo plus EC group were the most (Supplementary Table 3).

## Discussion

This multicenter, randomized, double-blind, placebo-controlled phase 3 study met its primary endpoint of OS, demonstrated that socazolimab combined with EC compared to EC have statistical significance and clinical benefits as the first line treatment in ES-SCLC patients. The addition of Socazolimab to chemotherapy improved OS by 2.32 months(13.90 m vs.11.58 m), and reduced the risk of death by 20.1%(HR 0.799 [95% CI 0.652–0.979]). Moreover, the outcomes of the secondary efficacy endpoints of progression-free survival and duration of response also showed significant benefit in the socazolimab plus EC group than in the placebo plus EC group.

In the IMpower133, the median OS was 2.0 months longer in the Atezolizumab group than in the placebo plus EC group (HR 0.76 [95% CI 0.60–0.95]).^16^ In the CASPIAN trials, Durvalumab combined with chemotherapy improved OS by 2.4 months. (HR 0.75 [95% CI 0.62–0.91]).^17^ Compared with these phase 3 trials, our study showed that adding Socazolimab to standard first-line chemotherapy has clinical benefits for patients, such as prolonging survival, consistent with the two trials mentioned above. Especially, in male patients aged under 65, socazolimab prolonged OS by 4.33 months compared to the control group (15.7 m vs. 11.37 m), which has never been seen in trials of other PD-1/PD-L1.

The prespecified subgroup analysis showed that patients had favorable OS with the treatment of socazolimab + EC regimen except female. Considering that female patients who received socazolimab had better PFS than those who received the placebo, we believe that the failure to contribute to survival was due to the influence of subsequent treatment. There is another theory that the response of women to chemotherapy is higher than that of men, and the tumor microenvironment and hormone status of women benefit less from immunotherapy than men.^18^ Subgroup analysis indicated an imbalance in OS benefit in patients aged 65 years or older, with younger patients significantly better. In this study, the proportion of the elderly in the socazolimab plus EC group (48.0%) was higher than that in the placebo plus EC group (39.5%). We believe that with a more balanced age distribution between two groups, the survival benefit could be enlarged. In addition, 66.9% of the socazolimab plus EC group and 65.3% of the placebo plus EC group were treated with corticosteroids during treatment. Considering the gastrointestinal adverse events and allergic reactions associated with EC based chemotherapy, this study allows for short-term corticosteroid pretreatment prior to chemotherapy, which may also be one of the factors affecting the efficacy of socazolimab Even so, the socazolimab plus EC group still maintained survival benefits.

It is worth noting that after disease progression or other reasons, 75.8% and 84.3% of patients in two groups received new anti-tumor treatments (mainly chemotherapy, but still 23.8% and 21.7% of patients used immunotherapy, respectively), which was much higher than in previous studies (treatment groups 44.2–59%; control groups 46–70%).^6–9^ Although the placebo plus EC group showed a higher proportion of subsequent anticancer therapy than the socazolimab plus EC group, the socazolimab plus EC group showed sustained survival benefits.

The maximum treatment period of the socazolimab was 2 years, considering that similar products based on other cancer types have a low recurrence rate and sustained response after discontinuation of treatment, long-term use may not increase benefits but increase the risk of related adverse events.^19^ A recent meta-analysis showed that there was no significant difference in OS rate between immunotherapy fixed duration (2 years) and indefinite immunotherapy (>2 years).^20^ The regimen does not limit the upper dose of carboplatin (e.g., 750 mg). Considering that the actual GFR of some patients does exceed 125 mL/min, limiting the carboplatin dose for these patients may lead to an underestimation of carboplatin AUC. On the premise that the patient can tolerate it, increasing the dose of carboplatin may not lead to higher toxicity, and the treatment effect can be better.^21^ Based on the results of this study, there was no significant difference in the incidence of adverse events of grade 3 or above among patients treated with carboplatin above and below 750 mg.

Safety profile of the study showed that the incidence of treatment related adverse events (TRAE) were generally similar between the two groups. Compared with other similar studies, the incidence of treatment-related grade 3 or above in the treatment group (80.3%) were higher than that in foreign similar studies (67.2% in Impower133 study, 62.8% in the Caspian study) and similar to the domestic studies (82.5% in ASTRUM-005 study, 80.6% in Capstone-1 study). The most common TRAE of grade 3 or above were hematological toxicities, which further confirmed that Asian patients were less tolerant to chemotherapy with the same dose regimen.^22,23^ The incidence of irAE in the treatment group was 19.3%, which was also similar, or lower than that of other studies (20.2%, 19.6%, 37.0%, and 28% in Impower133, Caspian, ASTRUM-005 and Capstone-1 studies, respectively). Three deaths were determined to be possibly related to the socazolimab combination, some due to severe underlying diseases and some due to deterioration of underlying diseases caused by chemotherapy intolerance.

There are several limitations regarding this study. We did not do a head-to-head study because at the specific time point when this study began, Atezolizumab was the only ICI that was approved as standard first line treatment of ES-SCLC in China but not covered by national medical insurance system. Considering the payment ability of most Chinese patients, EC regimen is still the first choice of standard treatment for most SCLC patients. Another limitation is that we failed to enroll enough patients with brain metastasis in either groups to draw a meaningful conclusion. This could be a result of a common problem that the exclusion tends to be strict when it came to patients with brain metastasis.^24^

In summary, the results of this phase 3 clinical trial indicate that socazolimab plus EC prolongs patient survival time compared to placebo plus EC in the first-line treatment of extensive-stage SCLC, without introducing any new safety risks. With the increase of product types, physicians can choose treatment plans more flexibly based on the specific clinical conditions of patients.

## Materials and methods

### Study design

This study is a randomized, double-blind, placebo-controlled multicenter III study. The study followed the Declaration of Helsinki and Good Clinical Practice guidelines. Written informed consent was provided by all patients, and the protocol and amendments were approved by all sites’ institutional review board and independent ethics committee.

### Patients

Key eligibility criteria wereage ≥18 years；histologically confirmed SCLC not previously systemic therapy for ES-SCLC that classified by the American Veterans Association for Lung Cancer; Eastern Cooperative Oncology Group (ECOG) performance status of 0 or 1; estimated survival time >8 weeks; at least one measurable lesion according to Response Evaluation Criteria in Solid Tumours version 1.1 (RECIST v1.1); adequate organ and bone marrow functional reserves; provision of tumor samples for biomarker PD-L1 assessment.

The main exclusion criteria include: history of autoimmune diseases; Received CTLA-4 inhibitors, PD-1 inhibitors, PD-L1/2 inhibitors, or other drugs targeting T cells; Brain metastases that require treatment; Use corticosteroids or other immunosuppressants within 14 days prior to randomization; clinical symptoms or diseases of the heart that cannot be well controlled; uncontrolled active infections; other malignant tumors occurred less than 5 years before randomization.

### Randomization and blinding

Eligible patients were randomly assigned to two groups in a 1:1 ratio. The general information of eligible patients were input into the Randomization and Trial Supply Management provided by an independent third party for randomization. Stratified block randomization (block size: 4) was adopted in this study. Random assignment was stratified by gender (male or female), ECOG PS (0 or 1) and brain metastasis (yes or no). Socazolimab and placebo were consistent in appearance and shape. The patients, investigators, and sponsors or their designated personnel were not aware of the grouping.

### Procedure

Socazolimab (5 mg/kg) or the corresponding placebo were treated intravenously every 21 days until disease progression, intolerable toxicity or death, or up to 2 years. In the chemotherapy regimen, carboplatin was given once within the area under the serum drug concentration time curve of 5 mg/mL/min (AUC 5) on the first day of each 21 days cycle, and etoposide was given continuously with 100 mg/m^2^ on the 1st, 2nd, and 3rd day of each 21 days cycle. The treatment of EC regimen was used until the completion of 4 cycles, with disease progression, intolerable toxicity, or death. During the treatment, the dose of reduction or suspension of EC was allowed, but socazolimab or placebo was only allowed for dose suspension up to 12 weeks. All dose modifications methods were shown in protocol. The main reason for choosing placebo combined with EC as the control group is that EC remains the recommended first-line treatment for ES-SCLC in China during the study design.^15^

During the research process, radiological examinations were conducted every 6 weeks or as deemed necessary by the central investigator,, and radiological evaluation was performed by the investigators and Independent Radiology Committee (IRC) respectively, but it was only up to the investigators to decide whether to continue treatment according to the evaluation results of the investigator.

### Outcomes

The primary end point of this study was OS, defined as the time from randomization to death. Key secondary outcomes included PFS (progression-free survival, defined as the time from random assignment to disease progression or death from any cause), ORR (objective response rate, defined as the proportion of patients with a complete response or partial response to treatment) and DoR(duration of response, defined as the time from onset of response to progression or death due to any reason, whichever occurs earlier) assessed by investigators or IRC based on RECIST v1.1, and OS rates at 1 and 2 years, adverse events, the association between PD-L1 expression levels in tumor tissues and efficacy, and other secondary outcomes including immunogenicity and quality of life that were not reported here.

Adverse events were recorded up to 90 days after the last dose of the entire study treatment and scored using the National Cancer Institute’s Common Terminology for Adverse Events (version 5.0). Treatment-related adverse events (TRAE) were defined as adverse events associated with either carboplatin, etoposide, socazolimab or placebo. Adverse events related to the use of ICI therapy are defined as immune-related adverse events (irAE).

Expression of PD-L1 was assessed by immunohistochemical method in formalin-fixed, paraffin-embedded tumor samples. The analyst stained PD-L1 positive paraffin tissue with PD-L1 antibody (Anti-PD-1 28-8) and used its isotype control antibody (Recombinant Rabbit IgG) as a control to confirm the specificity of the antibody and determine non-specific staining and/or background staining in the tissue.The definition of PD-L1 positivity is that tumor cells with partial or complete membrane staining account for at least 1% of the total live tumor cells.

### Statistical analysis

The calculation of sample size for clinical trials is based on the primary endpoint of OS. An estimated 5% patient dropout rate per year is planned to include 498 eligible patients. Assuming a median OS of 10.5 months for the placebo plus EC group and a required number of deaths of 369 to provide 85% of the detection power, the HR for death between the socazolimabplus EC group and the control group is set at 0.73 (based on CASPIAN study result.^7^)

An interim analysis was planned when ~222 (60% of the 369) expected deaths had occurred, a α-spending function of O’Brien-Fleming type is used to control the overall type I error rate. The final analysis would initiated when the cumulative number of deaths reaches 369. At the actual interim analysis cutoff date (March 20, 2023), As of the actual mid-term analysis deadline (March 20, 2023), there were 225 deaths (61.0%) (significant level, one sided *p* = 0.0041). In the interim analysis, compared with the placebo plus EC group, the HR of death in the socazolimab plus EC group was 0.73 (95%CI 0.56–0.96; one sided *p* = 0.0109), and did not reach the predetermined superiority boundary. Based on these results, the Independent Data Monitoring Committee recommended that the trial be continued according to the original design. As of October 13, 2023, there were 382 deaths and a final analysis of the OS was carried out, with the one-sided alpha value for hypothesis testing is 0.0236. The final statistical analysis results are presented here.

The full analysis set was used for efficacy evaluation, including all randomized patients who have received ≥1 study drug treatment. Among the patients included in the full analysis set, those with safety assessment data were all included in the safety analysis set.

The Kaplan-Meier method was used to estimate the efficacy endpoints, including OS, PFS, and DOR, and the 95% CIwas calculated using the Brookmeyer Crowley method. The survival data between the two groups was calculated using a stratified log rank test. Estimation of heart rate and 95% confidence interval using a stratified Cox proportional hazards model (gender, ECOG PS, and brain metastasis as stratification variables, treatment group as a covariate). Based on age, gender, ECOG status, brain metastasis, liver metastasis, disease stage, and PD-L1 expression, a stratified Cox proportional hazards model was used to conduct pre planned subgroup analysis of survival benefit indicators, including OS rate and PFS rate. The number and proportion of patients who achieved objective response in each group were calculated, and 95% CI of response rate was calculated based on Clopper Pearson method. Cochran-Mantel-Haenszel (CMH) chi-square test (gender, ECOG PS, and brain metastasis as stratification variables) was used to compare the objective response rate between two groups.

## Supplementary information

SAP

protocol

Supplementary Materials

## Data Availability

Thirty-six months after the completion of the study, the sponsor and the principal investigator can be requested to provide trial data that conceals personal information of participants in the results report of this study. Researchers need to submit an explanation of the reasons for requesting data. The principal investigator, sponsor, and clinical institution ethics committee will evaluate whether it affects the rights and interests of the patients. The study protocol and statistical analysis plan are provided.
