# Supplementary material for: A multicenter, randomized, double-blind, placebo-controlled phase 3 study of Socazolimab or placebo combined with carboplatin and etoposide in the first-line treatment of extensive-stage small cell lung cancer
Source: Signal Transduct Target Ther. 2025 Jan 13;10:28. doi: 10.1038/s41392-024-02115-5 (PMC11725569; doi:10.1038/s41392-024-02115-5)
Supplement: Supplementary file 1 — SAP [file 41392_2024_2115_MOESM1_ESM.docx]

Statistical Analysis Plan

**A multicenter, randomized, double-blind, placebo-controlled phase 3 study of Socazolimab or placebo combined with carboplatin and etoposide in the first-line treatment of extensive-stage small cell lung cancer**

**zkab001-lees-2020-07**

| **Investigational Medicinal Product:** | Recombinant anti-Human PD-1 functional monoclon injection (ZKAB001) combined with carboplatin and etoposide |
| --- | --- |
| **Comparator product:** | Placebo combined with carboplatin and etoposide |
| **Indications:** | First-line treatment of extensive-stage small cell lung cancer |
| **Staging:** | Phase III |
|  |  |
| **Document type:** | Statistical Analysis Plan |
| **Version:** | 1.0 |
| **Release Date:** | 11/01/2024 |

**Table of Contents**

[1 Introduction 5](#_Toc7882)

[1.1 Study Objectives 5](#_Toc8976)

[1.1.1 Primary Objective 5](#_Toc32491)

[1.1.2 Secondary Objectives 5](#_Toc32264)

[1.2 Study Endpoints: 5](#_Toc8856)

[1.2.1 Primary endpoints 5](#_Toc355)

[1.2.2 Secondary endpoints 5](#_Toc10496)

[1.3 Study Design 6](#_Toc10721)

[1.3.1 Summary of Study Design 6](#_Toc13573)

[1.3.2 Determination of Sample Size 10](#_Toc19317)

[1.3.3 Randomization and Blinding Plan 10](#_Toc32000)

[2 Estimand 10](#_Toc29821)

[2.1 Main Estimand 10](#_Toc27034)

[2.2 Secondary Estimand 11](#_Toc14267)

[2.2.1 PFS (RECIST v1.1 criteria) as assessed by the investigator and independent review committee (IRC) 11](#_Toc17501)

[2.2.2 ORR (RECIST v1.1 criteria) as assessed by the investigator and IRC 11](#_Toc31512)

[2.2.3 DoR as assessed by the investigator and IRC 12](#_Toc15346)

[2.2.4 DCRs as assessed by the investigator and IRC 12](#_Toc6518)

[2.2.5 OS rate at 1 and 2 years 12](#_Toc29475)

[3 Statistical Analyses General Consideration 13](#_Toc22558)

[3.1 Basic Principle 13](#_Toc14619)

[3.2 Analysing Sets 13](#_Toc29833)

[3.3 Multicentre Trial 14](#_Toc15485)

[3.4 Adjustments for Covariates 14](#_Toc30107)

[3.5 Multiple comparisons and multiplicity 14](#_Toc22592)

[3.6 Subgroup analysis 14](#_Toc903)

[4 Data-processing Principles 15](#_Toc20603)

[4.1 Derived Variable 15](#_Toc25586)

[4.1.1 Baseline and change relative to baseline 15](#_Toc18053)

[4.1.2 Time from first tumour diagnosis to enrolment 15](#_Toc25617)

[4.1.3 Therapy Day 15](#_Toc10002)

[4.2 Missing data 15](#_Toc2259)

[4.2.1 Missing efficacy data 15](#_Toc10803)

[4.2.2 Missing/incomplete dates 15](#_Toc23903)

[4.3 Data cut-off principle 18](#_Toc31845)

[4.4 Visit window 18](#_Toc14951)

[5 Statistical analysis Methods 19](#_Toc8469)

[5.1 Research Target 19](#_Toc29980)

[5.1.1 Patient Disposition 19](#_Toc25154)

[5.1.2 Protocol Deviation 19](#_Toc6861)

[5.1.3 Demographics and Baseline Characteristics 19](#_Toc32290)

[5.1.4 Previous history of tumour treatment 20](#_Toc18039)

[5.1.5 Medical history 21](#_Toc2800)

[5.1.6 Prior and co-medication 21](#_Toc18378)

[5.1.7 Previous and combined non-pharmacological treatment 21](#_Toc2651)

[5.1.8 Follow-up anti-tumour therapy 22](#_Toc20343)

[5.2 Efficacy Analyses 22](#_Toc12584)

[5.2.1 Primary Efficacy Endpoint 22](#_Toc12988)

[5.2.2 Secondary Efficacy Endpoint 24](#_Toc19703)

[5.3 Safety Analyses 26](#_Toc10382)

[5.3.1 Extent of Exposure 26](#_Toc16737)

[5.3.2 Adverse Events (AEs) 27](#_Toc13520)

[5.3.3 Clinical Laboratory Evaluations 30](#_Toc7870)

[5.3.4 Vital Signs 31](#_Toc23154)

[5.3.5 ECOG PS Score 31](#_Toc10321)

[5.3.6 Electrocardiogram (ECG) 31](#_Toc13173)

[5.3.7 Physical Exam 32](#_Toc3251)

[5.3.8 Other safety Exam 32](#_Toc17960)

[5.4 Immunogenicity Analysis 32](#_Toc27312)

[5.5 Quality of life analysis 32](#_Toc26897)

[5.5.1 EORTC QLQ-C30 Scores 32](#_Toc19957)

[5.5.2 EORTC QLQ-LC13 Scores 33](#_Toc21345)

[6 Planned Analyses 34](#_Toc27199)

[6.1 Interim Analysis 34](#_Toc9714)

[6.2 Final Analysis 35](#_Toc20711)

[7 Protocol Deviation 35](#_Toc22794)

[8 Statistical analysis tables/lists/charts 35](#_Toc31753)

[9 References 35](#_Toc7559)

1. Introduction

This statistical analysis plan is based on the "A multicenter, randomized, double-blind, placebo-controlled phase 3 study of Socazolimab or placebo combined with carboplatin and etoposide in the first-line treatment of extensive-stage small cell lung cancer", which was bid by Zhaoke(Guangzhou)Oncology Pharmaceuticals (No. ZKAB001-LEES-2020-07) was developed as a detailed description of the statistical analysis methods and data processing principles of the study protocol. The preparation of this statistical analysis plan is based on the protocol of the clinical study ZKAB001-LEES-2020-07 (version number: 1.2, version date: 27 April 2022).

- 1. Study Objectives
     1. Primary Objective

To evaluate the Overall Survival (OS) of ZKAB001 in combination with carboplatin and etoposide versus placebo in combination with carboplatin and etoposide in the first-line treatment of extensive-stage small cell lung cancer.

- - 1. Secondary Objectives
- To evaluate Progression Free Survival (PFS), Objective Response Rate (ORR), Disease Control Rate (DCR), During of Response (DoR), and OS rates at 1 and 2 years in ZKAB001 combined with carboplatin and etoposide versus placebo combined with carboplatin and etoposide in the first-line treatment of extensive-stage small cell lung cancer;
- Safety of treatment;
- Relationship between PD-L1 expression levels and clinical effects;
- Evaluation of the immunogenicity of ZKAB001;
- Evaluate quality of life before and after treatment.
  1. Study Endpoints:
     1. Primary Endpoints

OS.

- - 1. Secondary Endpoints
- PFS (RECIST v1.1 criteria) as assessed by the investigator and independent review committee (IRC);
- ORR (RECIST v1.1 criteria) as assessed by the investigator and IRC;
- DoR as assessed by the investigator and IRC;
- DCRs as assessed by the investigator and IRC;
- OS rates at 1 and 2 years;
- Incidence and severity of adverse events (AEs) and serious adverse events (SAEs) as assessed by CTCAE 5.0, and indicators of abnormal laboratory tests;
- PD-L1 expression levels in tumour tissues;
- Positive rate of Antidrug antibodies (ADA);
- Quality of life scores (evaluated using EORTC QLQ-C30 and QLQ-LC13).
  1. Study Design
     1. Summary of Study Design

This is a randomised, double-blind, placebo-controlled, multicentre phase III study. Patients eligible for enrolment will be randomised in a 1:1 ratio to either the treatment group: ZKAB001 + carboplatin + etoposide or the control group: placebo + carboplatin + etoposide, every 3 weeks is one treatment cycle. Stratification factors include gender (male/female), PS score (0/1), and brain metastasis (yes/no).

The study consisted of a Screening Period, a Treatment Period in which subjects received study treatment until there was confirmed disease progression, or an intolerable toxic reaction, or the maximum dosing cycle (2 years) was reached, or the subject voluntarily requested that study treatment be ended, and a Follow-up Period (consisting of both a safety follow-up and a survival follow-up component).

Study design:


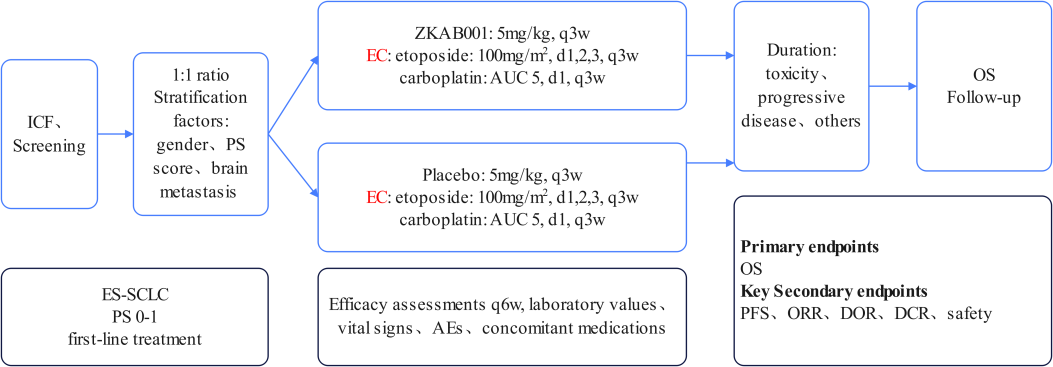


The detailed programme of the test is shown in Table 1 Table 1.

**Table 1 Schedule of Activity**

| visit  event | screening period^1^ | base line (in geodetic survey)^2^ | Treatment period (3 weeks (-3 to +7 days)) | | follow-up period | | |
| --- | --- | --- | --- | --- | --- | --- | --- |
|  |  |  |  |  | End-of-test inspection^3^ | security follow-up^4^ | Survival follow-up^5^ |
| Research time (days) | -28 to 1 day | -7 to 1 day | Per cycle - within 7 days | Every 2 cycles - within 7 days | Within 7 days of last treatment/withdrawal from study | +30 days (±7 days) after last treatment | Previous follow-up + 30 days (± 7 days) |
| Informed consent | **×** |  |  |  |  |  |  |
| Inclusion/exclusion criteria | **×** |  |  |  |  |  |  |
| Demographics/medical history/tumour-related treatment history^6^ | **×** |  |  |  |  |  |  |
| Confirmation and staging, including tumour tissue specimens^7^ | **×** |  |  |  |  |  |  |
| Blood/urine HCG^8^ | **×** |  |  |  | **×** |  |  |
| Vital signs, physical examination^9^ | **×** | **×** | **×** |  | **×** | **×** |  |
| PS Rating^10^ | **×** | **×** | **×** |  | **×** | **×** |  |
| Tumour Imaging^11^ | **×** |  |  | **×** | **×** |  |  |
| ECG^12^ | **×** |  | **×** |  | **×** | **×** |  |
| routine blood test^13^ | **×** | **×** | **×** |  | **×** | **×** |  |
| coagulation^14^ | **×** |  |  |  | **×** |  |  |
| blood biochemistry^15^ | **×** | **×** | **×** |  | **×** | **×** |  |
| Immunisation safety testing^16^ | **×** |  |  | **×** | **×** | **×** |  |
| HBsAg, HCV-Ab (or RNA), HIV antibodies^17^ | **×** |  |  |  |  |  |  |
| HBV-DNA (optional)^18^ | **×** |  |  | **×** |  |  |  |
| Urine and stool routine and occult blood^19^ | **×** | **×** | **×** |  | **×** | **×** |  |
| Survival follow-up |  |  |  |  |  | **×** | **×** |
| PD-L1 expression^20^ | **×** |  |  |  |  |  |  |
| Immunogenic blood collection^21^ | **×** |  |  |  |  |  |  |
| Quality of life score^22^ | **×** |  |  | **×** | **×** |  |  |
| Comorbidities, adverse events^23^ | **×** | | | | | |  |

1. Prior written informed consent must be obtained from the subject prior to the commencement of any clinical study procedure, except for screening such as tumour imaging that is available within a specified time frame prior to the first dose. Re-screening of previously unsuccessful subjects will be permitted, with re-signing of the informed consent form and re-enrolment with a new subject number.
2. Baseline data were recorded as the closest to the first dose of medication; if the screening period examination was no more than 1 week from the first dose of medication, the examination did not need to be repeated at baseline.
3. To confirm the safety of subjects at the end of treatment, an end-of-trial examination (EOT) is required within 7 days of their final treatment/confirmation of withdrawal from the study; assessments and examinations not administered and performed within the 7 days prior to confirmation of withdrawal from study treatment do not need to be repeated, and imaging should also be performed if 4 weeks have elapsed since the last imaging examination.
4. Safety should be followed up every 30 days ± 7 days for 90 days after the final dose, and the first time should be as close as possible to returning to the hospital for evaluation or an examination at a local hospital. If it is confirmed that more than one dosing cycle (3 weeks) has elapsed since the last dose at the time of withdrawal from the study, the end-of-trial check will be used as the first safety follow-up after the end of dosing. The next two follow-up visits may be by telephone to collect information on survival status, adverse events, concomitant medication and concomitant therapy only. Until death, loss to follow-up, initiation of other antineoplastic therapy, or completion of all three safety follow-up visits.
5. After termination of treatment and completion of safety follow-up, telephone follow-ups were conducted every 30 days (±7 days) to collect information on subject survival (date of death and cause of death) and information on the end of study treatment (including subsequent antitumour therapy received) until death or subject loss or termination of the study.
6. This includes the collection of information on past and current medical history, as well as previous/combined medications (between signing the ICF and the end of the screening period). For tumours, the specific regimen, start and end dates, and outcomes of all previous anti-tumour treatments (systemic or local, radiotherapy, chemotherapy, etc.) should be recorded.
7. Record the subject's histological diagnosis, time to first diagnosis, and time to progression or recurrence. Include specimen type and method of acquisition, pathological staging, stage II staging, and TNM staging.
8. Only for women of childbearing age who have not had an adnexectomy. A blood/urine HCG pregnancy test is required during the screening period, at the end of the trial.
9. Vital sign checks included temperature, pulse, respiration, and resting systolic and diastolic blood pressure. Vital sign checks were performed on the day of each dose, within 60 min before starting the drug infusion, and within 60 min after all therapeutic drug infusions were completed. Physical examination recorded abnormal findings, and new or worsening signs. A full set of physical examinations was completed prior to a single dose of drug and prior to the start of each cycle of infusion. Height measurements are performed only during the Screening Period. Weight measurements are taken during the Screening Period and prior to each cycle of treatment, but may be taken more frequently as needed.
10. PS scores were performed during the screening period and before each cycle of treatment, at the end-of-trial check, and at safety follow-up.
11. During the screening period, imaging should be performed every 6 weeks ± 7 days after treatment initiation, regardless of treatment delay or interruption, until progression or death. Each tumour imaging evaluation includes enhanced CT scans of the chest , abdomen, pelvis and sites with lesions. Enhanced CT was preferred, and only if there was a contraindication to enhanced CT (e.g., contrast allergy), a plain CT scan of the chest should be performed, and an enhanced MRI or CT scan of the other sites, except that magnetic resonance imaging (MRI) should be used preferentially for brain imaging. Imaging methods should be consistent throughout the trial period for the same subject (in terms of modality and use of contrast media, except for changing the method of examination due to contraindications to CT, and acceptance of the results of PET-CT for evaluation of bone lesions during the screening period and use of the results of bone scans at follow-up). A brain-enhanced MRI (preferred) or enhanced CT scan, as well as a bone scan, is also required during the screening period (if there is already a PET-CT-confirmed bone metastasis during this period, there is no need to repeat the bone scan). For unspecified sites with lesions, subsequent follow-up periods should be examined simultaneously.Receive baseline imaging performed at our centre within 28 days prior to treatment (brain and bone scans may be used with results from up to 42 days).
12. During the screening period, QTc will be recorded prior to each cycle of drug administration, and an echocardiogram may be added or performed in the presence of significant cardiac symptoms. Acceptance of the results at our centre within 28 days prior to treatment.
13. Routine blood counts including: white blood cell count, absolute neutrophil count, red blood cell count, haemoglobin, platelet count. Checked within 7 days prior to each ZKAB001 or placebo administration. Results within the first 7 days are accepted prior to the first dose.
14. Coagulation functions include: at a minimum, this should include International Normalised Ratio INR, Activated Partial Thromboplastin Time APTT, Fibrinogen FIB, Prothrombin Time TT. results within the first 7 days prior to acceptance of the first dose, examined during the Screening Period and at the time of withdrawal from the trial, and examined when clinically indicated during the study or according to the investigator's judgement.
15. Biochemical tests include: total bilirubin, direct bilirubin or indirect bilirubin, alkaline phosphatase, ghrelin, ghrelin, albumin, creatinine, urea or urea nitrogen, sodium, potassium, calcium, and glucose. Check within 7 days prior to each ZKAB001/placebo dose. Accept results within the first 7 days prior to the first dose.
16. Includes thyroid stimulating hormone, free T4 levels. Results within the first 7 days are accepted prior to the first dose and checked every 6 weeks.
17. Contains HBV, HCV, HIV tests, accepting results up to 28 days prior to first dose. Screening period tests with positive results for HIV/HCV were excluded based on exclusion criteria.
18. Patients who are positive for hepatitis B surface antigen need to complete HBV-DNA testing to rule out hepatitis B activity (unless the HBV-DNA titre is <500 IU/mL or the copy number is <1000 copies/ml after antiviral treatment), and patients who are positive need to have their HBV-DNA retested every 2 cycles, and antiviral treatment should be scheduled accordingly.
19. Routine urine tests include: urine specific gravity, urine pH, urine protein, urine glucose, urine leucocytes, urine red blood cells , within 7 days prior to each ZKAB001/placebo administration. Accept results within 7 days prior to first dose. Stool routine including: fecal occult blood.
20. No less than 5 white slices of specimen sections of tumour tissue (either previous or fresh), or less than 5 slices with the approval of the sponsor, are required for the detection of the expression level of PD-L1 in tumour tissue.
21. Depending on the order of enrolment, blood samples were collected once before ZKAB001/placebo administration for the first 100 subjects, once after dosing on the first day of the first cycle (C1D1), and once before dosing for C5D1 and C12D1. If a subject developed an infusion-related reaction, blood samples were retained as close as possible to the onset of the reaction, at the time of elimination, and approximately 30 days after the end of the reaction for comparative pre- and postimmunogenicity analyses. Blood collection from subjects experiencing a transfusion reaction was not limited to the first 100 cases. Blood samples were collected from venous blood at each of the above time points in serum separator tubes of approximately 3 mL, and the collected serum was divided equally into 2 cryostat tubes (1 for immunogenicity [ADA] and 1 for backup), which were placed in a cryogenic refrigerator and stored at -60 to -80°C for 6 months, and at -20°C in a refrigerator for 1 month, until transported to the central laboratory for testing.
22. Evaluation was performed using the EORTC QLQ-C30 and EORTC QLQ-LC13 scales: assessment was performed during the screening period, at each imaging assessment, and at the time of withdrawal from the trial, and a concurrent quality of life score was also required if unplanned imaging assessments were performed.
23. Adverse events that occur after signing informed consent and prior to administration of study drug should be documented in the medical history/current medical condition section of the CRF.
    - 1. Determination of Sample Size

In this study, OS was used as the primary endpoint, and the control group was tested for superiority. The parameters for calculating the sample size were as follows:

Efficacy assumptions:

- Median OS (months) = 10.5 months (control), HR = 0.73;

- Random ratio: 1:1;

- Enrolment and follow-up: 18 months for enrolment + 18 months for follow-up;

- Annual shedding rate: 5 per cent;

An interim analysis was planned to be done when the 3/5 event count (60% data maturity) was reached, using the O' Brien Fleming Class I error depletion function. If the OS test is 85% certainty, a total of 369 OS events are needed to reach the target event for both groups, approximately 498 subjects.

- - 1. Randomization and Blinding Plan

The study will be a randomised, double-blind design and screened subjects will be randomly assigned to either the treatment group or control group in a 1:1 ratio. Stratification factors include gender (male/female), PS score (0/1), and brain metastasis (yes/no).

1. Estimand
   1. Main Estimand

**The main clinical question of interest in this study:** the overall survival benefit of ZKAB001 in small cell lung cancer patients meeting Inclusion/exclusion criteria after treatment with ZKAB001 in combination with carboplatin and etoposide, in the context of the possibility of receiving other new antitumour therapies or the possibility of termination of treatment for any reason.

**Characteristics of the main estimand:**

1. **Target population:** The population as defined by the Inclusion/exclusion criteria.
2. **Target variable:** OS, defined as the time between the date of subject randomisation and death from any cause.
3. **Treatment:** treatment group: ZKAB001: 5 mg/kg/dose Q3W + etoposide: 100 mg/m^2^ D1,2,3 Q3W + carboplatin: AUC 5, D1 Q3W; control group: placebo 5 mg/kg/dose Q3W + etoposide: 100 mg/m^2^ D1,2,3 Q3W + carboplatin: AUC 5, D1 Q3W.
4. **Accompanying events and handling strategies:**
   1. Initiation of other new antineoplastic treatment: a therapeutic strategy is used, i.e. if a new antineoplastic treatment is initiated, follow-up is continued and the data collected is included in the analysis;
   2. Discontinuation of treatment for any reason: a therapeutic strategy is used, i.e., if treatment is discontinued, follow-up will continue and the data collected will be included in the analyses.
5. **Population-level summary:** risk ratios and 95% CIs for treatment and control groups.
   1. Secondary Estimand
      1. PFS (RECIST v1.1 criteria) as assessed by the investigator and independent review committee (IRC)

**CLINICAL QUESTION:** PFS benefit after treatment with ZKAB001 in combination with carboplatin and etoposide in patients with small-cell lung cancer who meet protocol entry criteria, assuming they are not receiving new antineoplastic therapy and may discontinue treatment for reasons other than disease progression.

**Characteristics of the estimand:**

1. **Target population:** same as main estimand.
2. **Target variable:** PFS as assessed by the investigator and IRC according to RECIST v1.1 criteria, defined as the time between the date of subject randomisation and any documented tumour progression or death from any cause, whichever occurs first.
3. **Treatment:** Same as main estimand.
4. **Accompanying events and handling strategies:**
   1. Initiation of new antineoplastic therapy: a hypothetical strategy was used, whereby the date of the last valid imaging examination up to the date of receipt of new antineoplastic therapy was censored if new antineoplastic therapy was initiated.
   2. Discontinuation of treatment for reasons other than disease progression: a therapeutic strategy is used, i.e., if treatment is discontinued for reasons other than disease progression, follow-up will continue and data collected will be included in the analysis.
5. **Population-level summary:** same as main estimand.
   - 1. ORR (RECIST v1.1 criteria) as assessed by the investigator and IRC

**CLINICAL QUESTION:** ORR benefit after treatment with ZKAB001 in combination with carboplatin and etoposide in patients with small-cell lung cancer who meet protocol entry criteria prior to receiving new antineoplastic therapy and/or termination of therapy due to disease progression, and who may be terminated for reasons other than disease progression.

**Characteristics of the estimand:**

1. **Target population:** same as main estimand.
2. **Target variable:** The investigator and IRC assessed whether the subject's BOR was CR or PR according to RECIST v1.1 criteria.
3. **Treatment:** Same as main estimand.
4. **Accompanying events and handling strategies:**
   1. Initiation of new antitumour therapy: using an on-treatment strategy, only data observed before the new antitumour therapy will be used for the analysis; data after the new antitumour therapy will not be used;
   2. Discontinuation of treatment due to disease progression: using an on-treatment strategy, only data observed prior to discontinuation of treatment due to disease progression are used in the analyses; data after discontinuation of treatment due to disease progression will not be used;
   3. Discontinuation of treatment for reasons other than disease progression: a therapeutic strategy is used, i.e., if treatment is discontinued for reasons other than disease progression, follow-up will continue and data collected will be included in the analysis.
5. **Population-level summary:** ORR and 95% CI for trial and control groups.
   - 1. DoR as assessed by the investigator and IRC

**CLINICAL QUESTION:** DoR benefit after treatment with ZKAB001 in combination with carboplatin and etoposide in patients with small cell lung cancer who meet protocol entry criteria, assuming that they are not receiving new antineoplastic therapy and may discontinue treatment for reasons other than disease progression.

**Characteristics of the estimand:**

1. **Target population:** same as main estimand.
2. **Target variable:** the DoR as assessed by the investigator and IRC according to the RECIST v1.1 criteria, defined as the time from the first evaluation of achieving objective remission (CR or PR) in subjects with a BOR of CR or PR to disease progression (PD) or death from any cause (calculated as the time when both occur first).
3. **Treatment:** same as main estimand.
4. **Accompanying Events and handling Strategies:** Same as PFS.
5. **Population-level summary:** same as main estimand.
   - 1. DCRs as assessed by the investigator and IRC

**CLINICAL QUESTION:** DCR benefit after treatment with ZKAB001 in combination with carboplatin and etoposide in patients with small-cell lung cancer who meet protocol entry criteria prior to receiving new antineoplastic therapy and/or termination of therapy due to disease progression, and who may be terminated for reasons other than disease progression.

**Characteristics of the main estimand:**

1. **Target population:** same as main estimand.
2. **OBJECTIVE VARIABLES:** The investigator and IRC assessed whether the subject's BOR was CR, PR, or SD according to RECIST v1.1 criteria.
3. **Treatment:** same as main estimand.
4. **Concomitant events and handling strategies:** same as ORR.
5. **Population-level summary:** DCR and 95% CI for trial and control groups.
   - 1. OS rate at 1 and 2 years

**CLINICAL QUESTION:** OS rate benefit after treatment with ZKAB001 in combination with carboplatin and etoposide in patients with small-cell lung cancer who meet the protocol entry criteria, in the context of the possibility of receiving other new antineoplastic treatments, or the possibility of discontinuation of treatment for any reason.

**Characteristics of the estimand:**

1. **Target population:** same as main estimand.
2. **Target variable:** whether subjects are alive at 1 and 2 years.
3. **Treatment:** same as main estimand.
4. **Accompanying events and handling strategies:** same as main estimand.
5. **Population-level summary:** 1- and 2-year OS rates and 95% CIs for trial and control groups estimated based on the Kaplan-Meier method.
6. Statistical Analyses General Consideration
   1. Basic Principle

All statistical analyses will be done using SAS 9.4.

Unless otherwise stated, all subjects enrolled and who used the trial medicinal product at least once were analysed by treatment group, and data tabulations are provided for all randomised subjects.

In general, continuous variables will be statistically described using the number of instances, mean, median, standard deviation (STD), minimum and maximum values; categorical and ranked variables will be statistically described using frequencies and percentages for each category or rank, and missing values will not be included in the computation of percentages, unless otherwise noted.

Statistical tests will be performed using a two-sided test with α = 0.05 and two-sided 95% confidence intervals (CIs) will be calculated if not otherwise specified.

- 1. Analysing Sets
- Full Analysis Set (FAS): all subjects randomised and receiving the trial drug at least once. The full analysis set will be used for analyses of subject distribution, demographic and baseline characteristics, and will also be used for the primary analysis of effectiveness.
- Per Protocol Set (PPS): a subset of subjects defined in the FAS set that excludes subjects with significant protocol deviations that are judged to have a significant impact on the outcome. The PPS will be used to support analyses of validity.
- Safety set (SS): all subjects who were randomised and received the trial drug at least once and for whom safety evaluation data are available. The safety analysis set is used for safety analyses.
- Immunogenicity analysis set: all subjects who were randomised and received the test drug at least once and had at least one immunogenicity evaluation. The immunogenicity analysis set is used for immunogenicity analysis.
  1. Multicentre Trial

Differences between centres will not be taken into account if not otherwise stated, data from all the different centres participating in this trial will be combined for analysis and centre will no longer be considered as a variable in the statistical analysis model.

- 1. Adjustments for Covariates

Unless otherwise stated, the corresponding baseline scores will be used as covariates in all models where quality of life analyses are conducted.

- 1. Multiple comparisons and multiplicity

This trial had only one primary endpoint, OS, and there were only two comparisons between treatment groups, so multiple comparisons were not necessary.

The test plan was to conduct an interim analysis when 3/5 OS events were reached, using the O'Brien Fleming class I error consumption function, and the actual number of OS events to calculate the validity bounds at a nominal test level of 0.0038, and a final analysis when 369 OS events were reached at a nominal test level of 0.0238. The final analysis will be adjusted appropriately if the final event count or interim analysis event count is slightly different from the plan (which may be caused by multiple events occurring on the same day as reaching the event count). analysis, if the final number of events or the number of events in the interim analysis differed slightly from the plan (possibly due to multiple events occurring on the same day that the number of events was reached), appropriate adjustments were made to the class I error bounds for the final analysis.

Analyses of secondary endpoints will all be treated as supportive/exploratory analyses and the statistical results of these analyses will be interpreted with caution.

- 1. Subgroup analysis

If the data permit, subgroup analyses of the primary efficacy indicator OS and secondary efficacy indicators PFS and ORR will be performed according to the following factors:

- Sex (male or female)
- Age (< 65 or ≥ 65)
- Disease Staging (Stage III or IV)
- Brain metastases (yes or no)
- Liver metastases (yes or no)
- Baseline tumour load (sum of target lesion diameters) assessed by investigator and IRC respectively (<10 cm, ≥10 cm)
- Number of baseline target lesions (<3, ≥3) assessed by investigator and IRC respectively
- Baseline PS score (0 or 1)
- Baseline PD-L1 expression level (<1%, 1%-10%, >10%)

If applicable, other demographic and baseline disease characteristic subgroups will also be analysed exploratively. Corresponding forest maps will be developed.

1. Data-processing Principles
   1. Derived Variable
      1. Baseline and change relative to baseline

For efficacy assessments, baseline was defined as the last non-missing assessment/inspection prior to randomisation; for safety assessments, baseline was defined as the last non-missing assessment/inspection prior to the first ZKAB001 or placebo administration. The value of change relative to baseline was calculated as the post-baseline assessment/examination value minus the baseline value.

- - 1. Time from first tumour diagnosis to enrolment

Time from first tumour diagnosis to randomisation (in months) = (date of randomisation - date of tumour diagnosis + 1) / 30.4375, retained to 1 decimal place.

- - 1. Therapy Day

Treatment day is defined as the number of days for efficacy assessment/safety relative to the reference date. For efficacy assessments, the date of randomisation will be used as the reference date; for safety assessments, the date of first ZKAB001 or placebo administration will be used as the reference date. The day of the reference date will be recorded as Day 1.

Treatment days are calculated as follows:

- Treatment day = date of assessment/examination - reference date + 1 if assessment/examination is on or after reference date maybe
- Treatment day = date of assessment/examination - reference date, if assessment/examination is before the reference date.
  1. Missing data

Unless otherwise noted, missing data are treated as missing and are not filled in with any assumed values.

- - 1. Missing efficacy data

The treatment of the efficacy analysis if the subject is lost to visit or the tumour assessment is missing, etc., is detailed in section 5.2 Efficacy analysis.

- - 1. Missing/incomplete dates

Unless otherwise noted, if the date is completely missing, it is not populated.

Date of tumour diagnosis

- If only the year of the date of diagnosis was recorded, 1 July was used to fill in the missing month and day of the date of diagnosis.
- If only the year and month of the date of diagnosis were recorded, 15 days were used to fill in the missing days in the date of diagnosis.
- If the post-populated metastasis date is earlier than the tumour diagnosis date, the tumour diagnosis date is used to populate the metastasis date.
- If the post-fill tumour diagnosis date is later than the informed consent signing date, the day before the informed consent signing date is used for the fill to ensure that the post-fill tumour diagnosis/metastasis date is no later than the informed consent signing date.

Adverse events/combinations

- If only the year and month are recorded for the adverse event/combination start date, the adverse event/combination start date will be populated as the date of the first ZKAB001/placebo infusion, assuming the year and month are the same as those of the first ZKAB001/placebo infusion date (if the populated adverse event/combination start date is later than the end date, the start date will be populated directly with the end date); otherwise the adverse event/combination start date will be populated as the first day (1st day) of the month in which the adverse event/combination started. directly); otherwise the Adverse Event/Combination Start Date will be populated as the first day (1st) of the month in which the Adverse Event/Combination started.
- If only the year is recorded for the adverse event/combination start date, the adverse event/combination start date will be populated as the date of the first ZKAB001/placebo infusion, assuming the year is the same as the year of the first ZKAB001/placebo infusion date (if the populated adverse event/combination start date is later than the end date, the start date will be populated directly with the end date); otherwise the adverse event/combination start date will be populated as the first day of the year in which the adverse event/combination started (1 January).
- If the adverse event/combination end date is incomplete, the adverse event/combination end date will be populated as the last day of the month in which the adverse event/combination ended or the last day of the year (31 December), but not later than the earliest of the study end date/date of death (if applicable).

Date of death

- If only the year and month are recorded for the date of death, the date of death will be populated with the last known survival date + 1 if the year and month are the same as the year and month of the last known survival date; otherwise the date of death will be populated with the first day of the month in which the date of death occurs (the 1st).
- If only the year is recorded for the date of death, the date of death will be populated with the last known date of survival + 1 if the year is the same as the year of the last known date of survival; otherwise, the date of death will be populated with the first day of the year in which the death occurred (1 January).
- If the date of death is completely missing, the date of death is filled in using the last known survival date + 1.

**Date of commencement of new anti-tumour therapy**

- If only days are missing
  - Year equal to minimum (maximum (date of disease progression + 1, date of end of treatment + 1, date of end of treatment imaging + 1), date of end of subsequent antineoplastic treatment)
    - Months less than the minimum (maximum (date of disease progression + 1, date of end of treatment + 1, date of end of treatment imaging + 1), date of end of subsequent antineoplastic treatment) were filled with the smaller of the last day of the month and the data cut-off date;
    - Months equal to the minimum (maximum (date of disease progression + 1, date of end of treatment + 1, date of end of treatment imaging + 1), date of end of subsequent antineoplastic therapy) are filled in with the minimum (maximum (date of disease progression + 1, date of last dose + 1, date of end of treatment imaging + 1), date of end of subsequent antineoplastic therapy);
    - Months greater than the minimum (maximum (date of disease progression + 1, date of end of treatment + 1, date of end of treatment imaging + 1), date of end of subsequent antineoplastic therapy) were filled with the smaller of the 1st of the month and the data cut-off date;
  - Years less than the minimum (maximum (date of disease progression + 1, date of end of treatment + 1, date of end of treatment imaging + 1), date of end of subsequent antitumour therapy) were filled with the smaller of the last day of the month and the data cut-off date;
  - Years greater than the minimum (maximum (date of disease progression + 1, date of end of treatment + 1, date of end of treatment imaging + 1), date of end of subsequent antineoplastic therapy) were filled with the smaller of the 1st of the month and the data cut-off date;
- If the month and day are missing
  - Years less than the minimum (maximum (date of disease progression + 1, date of end of treatment + 1, date of end of treatment imaging + 1), date of end of subsequent antineoplastic therapy) were filled with the smaller of 31 December and the data cut-off date;
  - Years equal to the minimum (maximum (date of disease progression + 1, date of end of treatment + 1, date of end of treatment imaging + 1), date of end of subsequent antineoplastic therapy) are filled in with the minimum (maximum (date of disease progression + 1, date of last dose + 1, date of end of treatment imaging + 1), date of end of subsequent antineoplastic therapy);
  - Years greater than the minimum (maximum (date of disease progression + 1, date of end of treatment + 1, date of end of treatment imaging + 1), date of end of subsequent antineoplastic therapy) were filled with the smaller of 1 January of the current year and the data cut-off date;
- For complete absence, the minimum value (maximum (date of disease progression + 1, date of end of treatment + 1, date of end of treatment imaging + 1), date of end of subsequent antitumour therapy) was used to fill in.
  1. Data cut-off principle

The interim and final analyses are planned to be carried out when the number of 222 and 369 events are known, respectively, and the data will be included in the analyses according to the following principles, based on the given "data cut-off date":

- Data collected on the basis of a visit: data collected under a visit with a date no later than the "data cut-off date";
- Adverse events: Adverse events with a start date no later than the "data cut-off date", if the end date is later than the "data cut-off date", the adverse event will be considered as "ongoing";
- Combination/non-pharmacological treatment: data on combination/non-pharmacological treatment with a start date no later than the "data cut-off date", if the end date is later than the "data cut-off date", the combination/non-pharmacological treatment will be considered as "Continuous";
- Subsequent antineoplastic therapy: Subsequent antineoplastic therapy with a start date no later than the "data cut-off date"; if the end date is later than the "data cut-off date", the new antineoplastic therapy is considered to be "continuous" after discontinuation. ";
- Tumour assessment: If at least one of the target/non-target/new lesion examination dates is not later than the "data cut-off date", all data for that assessment cycle are retained;
- Death records: Deaths with a date of death later than the "data cut-off date" are considered to be alive at the time of the data cut-off.
  1. Visit window

N/A. All analyses will be analysed as per the planned visit, without taking into account deviations from the visit window.

1. Statistical analysis Methods
   1. Research Target
      1. Patient Disposition

All subjects who sign informed consent will be included in the analysis.

If a subject withdrew from the study before randomisation, it was considered a screening failure. Report the total number of subjects who failed screening and the reason for screening failure, and tabulate the reasons for screening failure. Subjects with screening failures will not be included in additional analyses.

Summarise the number and percentage of cases where treatment ended and the reasons for treatment ending; similarly for the number of cases where the study ended and the reasons for study ending. Treatment closure and reasons for study closure will be presented in tables in the order they appear on the electronic case report form (eCRF). Tabular descriptions of study completion for all randomised subjects will include the date and reason for treatment end and the date and reason for study end.

In addition, the number of instances and percentages of subjects in each analysed dataset are summarised, using the number of instances of subjects randomised as the denominator in the calculation of percentages. The distribution of each analysed dataset and the reasons for exclusion from the analysed dataset are tabulated.

- - 1. Protocol Deviation

Programme deviations will be classified as minor or major. Programme deviations will be assessed and agreed upon by the researcher and the sponsor as "minor" or "major" during the data review prior to database lock. Final major programme deviations will be finalized and documented prior to database lock.

All randomised subjects were tabulated for major protocol deviations and all protocol deviations were analysed in a table.

- - 1. Demographics and Baseline Characteristics

The following demographic information and baseline disease characteristics were tabulated and summarised and tabulated separately.

- - - 1. Demographics
- Age (years)
- Age group (≥65, <65)
- distinguishing between the sexes
- nation
- Height (cm)
- Weight (kg)
- Body Mass Index (BMI) (kg/m^2^ )
- Body surface area (BSA) (m^2^ )
  - - 1. Baseline Disease Characteristics
- Time from first diagnosis to randomisation (months)
- Stage II at first diagnosis
- Second instalment at screening
- Current stage T, N, M staging
- Current clinical staging
- Progress or not
- Is there a recurrence
- Whether distant metastasis
- Site of metastasis (lungs, liver, kidneys, bones, brain, lymph nodes, etc.)
- Baseline PS score
- Baseline PD-L1 expression level (<1%, 1%-10%, >10%)
- Sum of total target lesion diameters (cm) assessed by the investigator and IRC respectively (mean, median, STD, minimum and maximum)
- Baseline tumour load (sum of target lesion diameters) assessed by investigator and IRC respectively (<10 cm, ≥10 cm)
- Number of target lesions at baseline (<3, ≥3) assessed by investigator and IRC respectively
  - - 1. Other Baseline Characteristics

Subjects' drug allergy history, history of previous non-oncological surgery and virological findings were tabulated according to treatment group.

- - 1. Previous history of tumour treatment

Summarise the number of cases and percentage of subjects for whom a history of previous antineoplastic treatment (medication, radiotherapy and surgical treatment history) exists. For drug treatment history, this will be further summarised by type of treatment regimen and will also be coded using the World Health Organisation Drug Dictionary (WHODRUG) Global 2021 Mar 1 or above, providing the preferred name (PN) and anatomical therapeutic chemistry (ATC) classification of the drug and summarised in terms of ATC Classification Level 2 and PN.

A tabular description of the history of previous antineoplastic therapy.

- - 1. Medical history

The history was coded using the International Medical Dictionary of Terms (MedDRA) version 24.0 or higher.

The histories were summarised by System Organ Classification (SOC) and Preferred Terminology (PT) and the number of cases and percentage of subjects were calculated.

A tabulation of the subject's medical history.

- - 1. Prior and co-medication

Previous/combined medications were coded using the World Health Organisation Drug Dictionary (WHODRUG) Global 2021 Mar 1 or above, providing the preferred pharmacological name (PN) and anatomical therapeutic chemical (ATC) classification of the drug. Previous and combined medications were summarised separately by ATC classification level 2 and PN, and the number of cases and percentage of subjects were calculated:

- Prior medication, i.e., medication used only prior to the start of the first ZKAB001/placebo infusion (i.e., medication had ended prior to administration);
- Co-administered medications, defined as medications that were not discontinued prior to the start of the first ZKAB001/placebo infusion or started after the start of the first ZKAB001/placebo infusion.

If the timing of the drug relative to the first ZKAB001/placebo infusion cannot be determined, the drug is considered to be coadministered.

Previous/combined medications of the subjects were tabulated separately.

- - 1. Previous and combined non-pharmacological treatment

Prior and combined non-pharmacological treatments were coded using MedDRA version 24.0 or above.

Previous, combined non-pharmacological treatments were summarised separately by System Organ Classification (SOC) and Preferred Terminology (PT), respectively, and the number of cases and percentage of subjects were calculated:

- Prior non-pharmacological treatment, i.e. non-pharmacological treatment used only prior to the start of the first ZKAB001/placebo infusion (i.e. treatment had ended prior to administration);
- Combined non-pharmacological treatments, i.e., non-pharmacological treatments that remain in use during the treatment period (medications not discontinued prior to the first ZKAB001/placebo infusion or initiated after the start of the first ZKAB001/placebo infusion).

Non-pharmacological treatments were considered to be combined if the timing of the non-pharmacological treatment relative to the time of the first ZKAB001/placebo infusion could not be determined.

Separate tabular descriptions of subjects' prior/combined non-pharmacological treatments.

- - 1. Follow-up anti-tumour therapy

The number of cases and percentage of subjects who have received subsequent antineoplastic therapy will be calculated by treatment group and the number of cases and percentage of subjects who have received each type of subsequent antineoplastic therapy will be summarised separately by type of treatment (radiotherapy, surgical treatment, chemotherapy, etc.). For subsequent antineoplastic drug treatments, this will also be coded using WHODRUG Global 2021 Mar 1 or above, providing the PN and ATC classification of the drug and summarised according to ATC Classification Level 2 and PN; for subsequent antineoplastic non-pharmacological treatments, this will also be coded using MedDRA Version 24.0 or above, providing the SOC and PT classification and summarised .

The median time to receive subsequent antineoplastic therapy was estimated for each treatment group using the Kaplan-Meier method, and a bilateral CI was constructed based on the Brookmeyer-Crowley method with a double logarithmic (Log-Log) transformation to report the rate of receiving subsequent antineoplastic therapy at different time points (1 year, 2 years, etc.), and calculated based on the Greenwood formula to calculate its corresponding Bilateral CIs. time to receipt of follow-up antineoplastic therapy was defined as the time from randomisation to initiation of follow-up antineoplastic therapy, and if subjects had not received follow-up antineoplastic therapy at the time of data cut-off, the date of the last visit was used as the censored date in the analyses. Survival functions for the two treatment groups will be compared using stratified Log-rank tests. In addition, a stratified Cox proportional risk model will be used to estimate the hazard ratio (HR) and its two-sided CI for comparisons between the two treatment groups and to provide a p-value for comparisons between the treatment groups; in the case of a tie (tie), this will be dealt with using the Efron method.

A detailed list of new anti-tumour treatments after the end of treatment.

- 1. Efficacy Analyses

Imaging data will be evaluated by the investigators at each study centre and by an independent imaging evaluation committee (IRC) according to RECIST v1.1 criteria. Imaging-related efficacy analyses will be analysed based on both investigator and IRC evaluations.

The analysis of efficacy involves stratification based factors that will be analysed based on stratification at randomisation in the IWRS .

- - 1. Primary Efficacy Endpoint

The primary efficacy endpoint was overall survival (OS), defined as the time between the date of subject randomisation and death from any cause. If the subject is alive at the time of data cut-off, the date the subject was last known to be alive will be used as the censored date in the OS analysis; if no survival information is provided for the subject after the baseline period, the date of randomisation will be used as the censored date in the OS analysis; and if the subject dies but the date of death is missing or incomplete, it will be populated by using the method of filling in the missing dates as defined in 3.2.2.

The trial will perform the main OS analysis when 369 OS events are reached.

The hypothesis testing for OS is as follows:

H_0_ : OS(t)_treatment group_ = OS(t)_control group_ ;

H_1_ : OS(t)_treatment group_ ≠ OS(t)_control group_ .

Here OS(t) denotes the survival function of OS at time t, t ≥ 0.

- - - 1. Master Analysis

Based on the FAS, the number of cases of subjects who died and censored and their percentages were described separately for each treatment group. Survival function estimates and first-, median-, and third-quartile OS estimates were obtained using the Kaplan-Meier method, and two-sided confidence intervals (CIs) corresponding to OS were constructed using the Brookmeyer-Crowley method with double logarithmic (Log-Log) transformation. The overall survival rates at different time points (6 months, 12 months, 18 months, 24 months, etc.) were also reported and their corresponding bilateral CIs were calculated based on Greenwood's formula.Kaplan-Meier survival curves were plotted.

Survival functions were compared between the two treatment groups using a stratified log-rank test, with stratification factors including sex (male or female), brain metastasis (presence or absence of brain metastasis), and PS status (0 or 1).

In addition, a stratified Cox proportional risk model will be used to estimate the hazard ratio (HR) and its two-sided CI for comparisons between the two treatment groups and to provide a p-value for comparisons between the treatment groups; if there is a tie (tie), this will be dealt with using the Efron method.

- - - 1. Sensitivity Analysis

Based on the FAS, the following analyses were performed following a similar approach to the main analysis:

- No stratification is considered;
- If the percentage of inconsistency between actual and random stratification is greater than 5 per cent, the analysis will be based on actual stratification;
- To avoid over-stratification, if the number of OS events in a stratum was less than 10, a stratification factor containing the minimum number of subjects was removed until the number of OS events in all strata was greater than 10.
  - - 1. Subgroup Analysis

Subgroup analyses will be performed to assess the consistency of treatment effects between different subgroups by subgrouping the OS ratings of subjects according to the subgroups defined in 3.6, and forest plots will be drawn.

- - - 1. Supplementary Analysis

Supplementary Analyses 1 and 2: The robustness of the results was interpreted based on all randomised subjects and PPS respectively following a similar methodology to the main analysis and compared with the results of the main analysis.

Supplementary analysis 3: For the concomitant event "receiving other new antineoplastic treatment", supplementary analyses will be conducted using a hypothetical strategy, in which subjects receiving other new antineoplastic treatment are censored to the date of the start of the new antineoplastic treatment and analyses are conducted in a similar way to the main analyses, adjusting for the effect of the other new antineoplastic treatment on OS. . If data were applicable, subjects receiving other new antineoplastic treatments were also considered to be treated as with Informative Censoring, e.g., using the Inverse Probability Censoring Weighting (IPCW) method.

Supplementary Analysis 4: If the proportional risk assumption does not hold, the results of analyses based on the Restricted Mean Survival Time (RMST) approach will also be provided.

- - 1. Secondary Efficacy Endpoint

Efficacy metrics will be evaluated by the investigators according to RECIST v1.1, and progression-free survival (PFS) and objective remission rate (ORR) will also be evaluated by independent imaging (IRC) according to RECIST v1.1.

- - - 1. Progression Free Survival (PFS )

PFS results based on IRC and investigator-assessed PFS were analysed separately.

PFS was defined as the time between the date of subject randomisation and any documented tumour progression or death from any cause, whichever occurred first.

PFS incident, deletion processing principles are shown in Table 2 . If the subject meets the Table 2 multiple conditions in Table 2, the earliest date will be used in the analyses.

**Table 2 Incident, deletion handling principles for PFS**

| **position** | **Dates used in the analysis** | **PFS ending** |
| --- | --- | --- |
| Baseline tumour evaluations not completed or missing | Random date | delete |
| Progress between planned visits | Date of progress | make headway |
| No progress | Date of last valid imaging | delete |
| Discontinuation of administration due to non-confirmatory progression | Date of last valid imaging | delete |
| Termination of administration due to toxicity or other reasons | Date of last valid imaging | delete |
| Initiation of new anti-tumour therapy | Date of last validated imaging before receiving new antineoplastic therapy | delete |
| Death before first progress assessment | Date of death | make headway |
| Deaths between full assessment visits | Date of death | make headway |
| Progression or death after 2 or more consecutive missing tumour assessments | Date of last validated imaging prior to progression | delete |

The PFS will be analysed based on the FAS using statistical methods similar to those used in the main OS analysis, with additional analyses based on all randomised subjects and the PPS.

Based on the FAS, IRC and investigator-assessed PFS outcomes (PD, censored) will be cross-tabulated according to treatment group, and the consistency of IRC and investigator-assessed PFS evaluation results will be analysed using the McNeamar test. In addition, the number and proportion of subjects with IRC PFS times earlier or later than the investigator-assessed PFS times will be summarised.

Subgroup analyses of IRC and investigator-assessed PFS outcomes for subjects in the FAS analysis set according to the subgroups defined in section 2.6 were performed separately to assess the consistency of treatment effects across subgroups, and forest plots were drawn.

- - - 1. Objective Response Rate (ORR)

The results of ORR evaluation based on IRC and researcher were analysed separately.

ORR was defined as the number of cases in which the best overall outcome during the study was complete remission (CR) and partial remission (PR) maintained for more than 4 weeks as a percentage of subjects with evaluable efficacy. The number of cases and percentage of subjects achieving objective remission in each treatment group was calculated and the 95% CI for ORR was calculated based on the Clopper Pearson method.The Cochran-Mantel-Haenszel (CMH) chi-square test was used to compare the ORR of the test group with that of the control group, and the p-value was calculated for the comparison of the two treatment groups.Stratification factors in the CMH test were considered the same as the randomised stratification factors. The difference in the proportion of ORR in the test group relative to the control group and its 95% CI (stratified Miettinen-Nurminen method) will be further calculated. The number and proportion of subjects achieving objective remission in FAS will also be calculated and the 95% CI of ORR will be calculated and compared between groups in a similar way as described above.

In addition, the number and proportion of cases with different best efficacy ratings (CR, PR, SD, PD and NE) and the corresponding 95% CI will be calculated.The concordance between IRC and investigator-rated best efficacy will be analysed by calculating the proportion of subjects with different best efficacy ratings by the IRC and the investigator and the Bowker's test will be used to explore whether the differences in best efficacy ratings are biased.

The ORR results of the IRC evaluations of the full analysis set of subjects were stratified separately according to the subgroups defined in section 2.5 to assess the consistency of the treatment effects between the different subgroups. Forest plotting.

- - - 1. During of Response (DoR)

The results of the DoR evaluation were analysed based on the IRC and the researcher.

DoR was defined as the time from the first evaluation of achieving objective remission (CR or PR) to disease progression (PD) or death from any cause (whichever occurs first) for subjects with a best efficacy rating of CR or PR. DoR was event and censored according to the principles of event, censored treatment for PFS.DoR was calculated only for subjects with a best efficacy rating of CR or PR.

DoR was analysed using statistical methods similar to those used in the main PFS analysis.

- - - 1. Disease Control Rate (DCR)

The results of the DCR evaluation were analysed based on IRC and the researcher.

DCR was defined as the proportion of subjects with a best efficacy rating of CR or PR who achieved a confirmed objective remission (CR or PR evaluated according to RECIST v1.1 criteria and maintained for more than 4 weeks) or stable disease (SD) maintained for more than 6 weeks during the course of the study.

The DCR will be analysed using statistical methods similar to those used for ORR analysis.

- 1. Safety Analyses
     1. Extent of Exposure

The total number of cycles of treatment in which subjects actually received the test drug (ZKAB001 or placebo) and the combination (carboplatin, etoposide) were analysed descriptively (mean, median, STD, minimum and maximum values), respectively, and the number of cases and percentage of subjects receiving at least a certain number of cycles of treatment (≥1, ≥2, ≥3, ≥4, ≥5, ≥6, etc.) was summarised.

Subjects were descriptively analysed for total drug exposure time, total actual dose administered, actual intensity of administration and relative intensity of administration:

ZKAB001 or placebo (5 mg/kg/dose IV every 3 weeks)

- Duration of drug exposure (weeks) = (date of last dose - date of first dose + 21) ÷ 7
- Total actual dose administered (mg/kg) = sum of all actual doses administered
- Actual strength of drug administered (mg/kg/week) = Total dose actually administered (mg) ÷ Duration of drug exposure (weeks)
- Scheduled strength of administration (mg/kg/week) = 5 mg/kg/dose ÷ 3 weeks
- Relative strength of administration (%) = Actual strength of administration (mg/kg/week) ÷ Scheduled strength of administration (mg/kg/week) × 100%.

Carboplatin (AUC 5, IV every 3 weeks)

- Drug exposure time (weeks) = (date of last dose - date of first dose + 21) ÷ 7
- Total actual dose administered (mg/ml/min) = sum of all actual doses administered
- Actual strength of drug administered (mg/ml/min/week) = total actual dose administered (mg/ml/min) ÷ duration of drug exposure (weeks)
- Scheduled dosing strength (mg/ml/min/week) = 5 mg/ml/min ÷ 3 weeks
- Relative strength of administration (%) = Actual strength of administration (mg/week) ÷ Scheduled strength of administration (mg/week) × 100%.

Etoposide (100mg/m^2^ , IV every 3 weeks)

- Duration of drug exposure (weeks) = (date of last dose - date of first dose + 21) ÷ 7
- Total actual dose administered (mg/m^2^ ) = sum of all actual doses administered
- Actual strength of drug administered (mg/m^2^ /week) = Total actual dose administered (mg/m^2^ ) ÷ Duration of drug exposure (weeks)
- Scheduled dosing strength (mg/m^2^ /week) = 100mg/m^2^  ÷ 3 weeks
- Relative dosing intensity (%) = Actual dosing intensity (mg/m^2^ /week) ÷ Planned dosing intensity (mg/m^2^ /week) × 100%.

In addition, the number and percentage of subjects in whom at least one adjustment (delayed dosing, permanent discontinuation, infusion interruption of dosing, etc.) occurred throughout the trial and the reasons for their occurrence will be analysed separately and in summary.

The administration of ZKAB001/placebo, carboplatin, and etoposide are tabulated separately.

- - 1. Adverse Events (AEs)

Adverse events (AEs) were coded according to MedDRA version 24.0 or higher.

A treatment-emergent or worsening adverse event (TEAE) was defined as an adverse event that occurred in a subject after initiation of ZKAB001/placebo up to 90 days after the last dose of ZKAB001/placebo or prior to initiation of a new antineoplastic therapy, whichever occurred first, or an exacerbation or worsening of a pre-dose adverse event or a pre-existing persistent medical condition. An adverse event is considered a TEAE if the timing of the adverse event relative to the start of ZKAB001/placebo administration cannot be determined.

Summarises only TEAEs, but provides a list of data for all AEs.

If the time of administration of AE relative to the first study drug could not be determined, it was considered a TEAE.

Only TEAEs were analysed on a pooled basis. Only separate data listings are provided for AEs defined as present before treatment.

- - - 1. TEAE Summary Sheet

A TEAE summary table is given that reports the number of cases, percentage of subjects who experienced at least 1 TEAE, and the number of cases of TEAE:

- All TEAE
- TEAE associated with any of the trial medicines (associated is defined as "definitely associated", "probably associated", "possibly associated", TEAE with missing association with ZKAB001/placebo/carboplatin/etoposide is also considered associated). TEAEs with missing relationships to ZKAB001/placebo/carboplatin/etoposide are also considered relevant)
- TEAEs related to ZKAB001/placebo (relatedness is defined as "definitely related", "probably related", "possibly related", and TEAEs with no relationship to ZKAB001/placebo are also considered related). (defined as "definitely related", "probably related", "probably related", and TEAEs with a missing relationship to ZKAB001/placebo are also considered related)
- TEAE at CTCAE level 3 or above
  - CTCAE level 3 or higher TEAE associated with any of the test medicines
  - TEAE with CTCAE grade 3 or higher associated with ZKAB001/placebo
  - Carboplatin ≥ 750mg CTCAE grade 3 or higher TEAE
  - Carboplatin <750mg CTCAE Grade 3 or higher TEAE
  - TEAE at CTCAE level 3 or above aged ≥65 years
  - TEAE at CTCAE level 3 or above at age <65 years
- Immune-related adverse events (irAE)
- CTCAE level 3 or higher irAE
- Infusion or anaphylactic reaction (AE with "Yes" for "Is infusion-related reaction" or "Hypersensitivity reaction" for PT in CRF)
- CTCAE grade 3 or higher infusion or allergic reaction
- TEAE leading to suspension of ZKAB001/placebo dosing
  - Related to any of the test drugs
  - Related to ZKAB001/placebo
- TEAE leading to termination of ZKAB001/placebo dosing
  - Related to any of the test drugs
  - Related to ZKAB001/placebo
- TEAE leading to reduced chemotherapy (carboplatin/etoposide) dosing
  - Related to any of the test drugs
  - Related to ZKAB001/placebo
- TEAE leading to suspension of chemotherapy (carboplatin/etoposide) administration
  - Related to any of the test drugs
  - Related to ZKAB001/placebo
- TEAE leading to termination of chemotherapy (carboplatin/etoposide) administration
  - Related to any of the test drugs
  - Related to ZKAB001/placebo
- Serious Adverse Events (SAEs)
- SAEs related to any of the test drugs
- SAEs related to ZKAB001/placebo
- TEAE leading to death
- TEAEs associated with either test drug that resulted in death.
- TEAE associated with ZKAB001/placebo that resulted in death.
  - - 1. TEAE analysed by SOC and PT

For the following TEAEs, report the number of cases, percentage of subjects who experienced a TEAE, and the number of cases of TEAE by MedDRA SOC and PT:

- All TEAE
- TEAE associated with any of the test medicines
- TEAE associated with ZKAB001/placebo.

The number of cases and percentage of subjects experiencing a TEAE will also be summarised for all TEAEs, TEAEs associated with ZKAB001/placebo, and TEAEs associated with any of the investigational medicines, according to the MedDRA SOC, PT, and severity (CTCAE grades 1-5). If multiple TEAEs with the same SOC or PT occur in the same subject, that subject will be counted only once at the corresponding SOC or PT level according to the highest CTCAE classification.

- - - 1. Adverse events of special concern

irAE, infusion, or allergic reactions were the AEs of special interest (AESIs) in this trial. The number and percentage of subjects who experienced the following AESIs and the number of instances of AESIs in each treatment group were reported according to MedDRA SOC, PT:

- irAE
- CTCAE level 3 or higher irAE
- Infusion or allergic reaction
- CTCAE Grade 3 or greater infusion or allergic reaction.

The time to presentation of the first AESI (any CTCAE grade, CTCAE grade 3 or higher) and the time to remission of the first AESI will be analysed descriptively. In addition, time to presentation of the first AESI and time to remission of the first AESI will be analysed using the Kaplan-Meier method to obtain the median time and its 95% CI, if the data allow.

Time to first AESI was defined as the time from the start of the first ZKAB001/placebo infusion to the first occurrence of an AESI. If a subject's AESI had not occurred by the time of data cut-off , the earliest of the four dates of the last ZKAB001/placebo infusion date + 90 days, data cut-off, time of loss to visit, or time of death was used as the censoring date in the analyses.

The time to remission of the first AESI was defined as the time from the presentation of the first AESI to remission (AE outcome collected in the CRF as "recovered/healed" or "recovered/healed with sequelae"). If the AESI was not in remission at the time of data cut-off, the earliest of the date of the last ZKAB001/placebo infusion + 90 days, the data cut-off, the time of loss to follow-up, or the time of death was used as the censored date in the analyses.

If there are multiple AESIs with the same start date when determining the first AESI and calculating the mitigation time, the one with the longer mitigation time is selected as the mitigation time for the first AESI.

- - - 1. Deaths, SAEs and other important TEAEs
         1. Deaths

A summary description of when the death occurred and the cause of the death (adverse event, disease progression, other, etc.):

- All dead;
- Deaths during treatment, defined as deaths between the start of the first ZKAB001/placebo infusion and the first safety follow-up visit (within 30 days ± 7 days after the last treatment);
- Deaths during safety follow-up were defined as deaths occurring from the first safety follow-up until 90 days after the last dose;
- Deaths during the survival follow-up period were defined as deaths during the survival follow-up period.

In addition, the number and percentage of subjects for whom a death occurred during treatment will be analysed by SOC and PT.

- - - - 1. SAE

Using a similar approach to the TEAE analysis, the following SAEs are described in summary by SOC and PT:

- SAE;
- SAEs related to any of the test drugs
- SAEs associated with ZKAB001/placebo.

Tabulate all SAEs that occurred during the trial.

- - - - 1. Other important TEAEs

Using a similar approach to the TEAE analysis, the following significant TEAEs are described in summary by SOC and PT:

- TEAE leading to suspension of ZKAB001/placebo dosing
- TEAE leading to termination of ZKAB001/placebo dosing
- TEAE leading to reduced chemotherapy (carboplatin/etoposide) administration
- TEAE leading to suspension of chemotherapy (carboplatin/etoposide) administration
- TEAE leading to termination of chemotherapy (carboplatin/etoposide) administration
  - 1. Clinical Laboratory Evaluations

Laboratory tests include routine blood, urine and stool and occult blood, blood biochemistry, coagulation, and immune safety tests.

Minimum and maximum values over the trial period were summarised for quantitative laboratory test indicators, and observations and changes relative to baseline were tabulated (number of subject cases, mean, STD, median, minimum and maximum values) were summarised.

Abnormal results and toxicity grades of applicable laboratory tests were judged according to NCI CTCAE version 5.0. A cross-tabulation of the change in CTCAE grade of the laboratory test from baseline to the time point with the most severe post-baseline toxicity grade, and the last post-baseline time point, is given. In addition, the number of cases and percentage of subjects with at least one post-baseline laboratory test CTCAE grade increase and post-baseline CTCAE grade increase to grade 3 or 4 will be summarised according to the toxicity designation given in CTCAE version 5.0.

The worst clinically significant time point from baseline to post-baseline is given using the categories "normal", "abnormal not clinically significant" and "abnormal clinically significant" according to the investigator's judgement, A cross-tabulation of the change in clinical significance of laboratory tests from baseline to the worst post-baseline clinical significance time point, and the last post-baseline time point.

All laboratory tests will be tabulated and all test values outside the normal laboratory value range will be labelled. In addition, laboratory tests with increased CTCAE grades from baseline will be tabulated separately.

- - 1. Vital Signs

Minimum and maximum values during the trial period were summarised for vital signs, and observations and changes relative to baseline were tabulated (number of subject cases, mean, STD, median, minimum and maximum values).

Vital Signs List Description.

- - 1. ECOG PS Score

A cross-tabulation of the change in ECOG scores from baseline to the worst (highest) post-baseline ECOG score at the time point, and the last post-baseline ECOG score at the time point, is given.

A description of the ECOG scoring list.

- - 1. Electrocardiogram (ECG)

Minimum and maximum values during the trial period were summarised for ECG parameters, and observations and changes relative to baseline were tabulated (number of subject cases, mean, STD, median, minimum and maximum values).

The worst clinically significant time point from baseline to post-baseline is given using the categories "normal", "abnormal not clinically significant" and "abnormal clinically significant" according to the investigator's judgement, The change in clinical significance from baseline to the last post-baseline time point is given.

A description of the ECG list.

- - 1. Physical Exam

Tabular description of physical examination findings with at least one new clinically significant abnormality during the treatment period.

- - 1. Other safety Exam

Tabular description of HBV-DNA, HCV-RNA, pregnancy test, echocardiography, etc.

- 1. Immunogenicity Analysis

Immunogenicity analysis will be based on the ADA analysis set.

Summarise the number of cases and percentage of subjects who were ADA positive in the following categories by treatment group:

- ADA positive at any visit during the trial (including baseline)
- Positive ADA at baseline
- Negative ADA at baseline but positive ADA at either post-baseline visit.

For ADA-positive subjects, further descriptive analyses of the results of their antibody titres, and possibly the time of onset and duration of ADA positivity, will be performed, and the number and percentage of subjects who are neutralising antibody (Nab)-positive will be calculated. If the incidence of ADA positivity is high, the impact of ADA positivity on the primary efficacy endpoints and primary safety outcomes will be further explored.

ADA and Nab results were tabulated for the subjects.

- 1. Quality of life analysis
     1. EORTC QLQ-C30 Scores

The EORTC QLQ-C30 has a total of 30 entries, which can be categorised into 15 areas, including:

- 5 domains of functioning (somatic, role, cognitive, emotional and social functioning)
- 3 symptom areas (fatigue, pain, nausea and vomiting)
- 1 total health status/quality of life domain
- 6 single entries (each as a field).

Specific raw scores (RS) are calculated as follows:

- Total health status (QL2) = (Q29+Q30)/2
- Functional areas:
- Domain of somatic functioning (PF2) = (Q1+Q2+Q3+Q4+Q5)/5
- Role Functional Area (RF2) = (Q6+Q7)/2
- Emotional Functioning Domain (EF) = (Q21+Q22+Q23+Q24)/4
- Domain of Cognitive Functioning (CF) = (Q20+Q25)/2
- Social Functioning Area (SF) = (Q26+Q27)/2
- Symptom domains and 6 single entries:
- Fatigue (FA) = (Q10+Q12+Q18)/3
- Nausea and vomiting (NV) = (Q14+Q15)/2
- Pain (PA) = (Q9 +Q19)/2
- Shortness of breath (DY) = Q8
- Insomnia (SL) = Q11
- Loss of appetite (AP) = Q13
- Constipation (CO) = Q16
- Diarrhoea (DI) = Q17
- Financial hardship (FI) = Q28

If missing data are encountered during the calculation of a raw score, the average of other non-missing questions may be used in place of the missing data, as long as the number of missing questions does not exceed half of the total number of questions required for that raw score. If the number of missing questions exceeds half of the total number of questions required for the raw score, the raw score is set as missing.

In order to make the domain scores comparable with each other, the raw scores will be further transformed linearly into standardised scores (SS) within 0 to 100:

- Overall health status: SS = (RS-1) /6 × 100.
- Functional area: SS = [1-(RS-1) /3] × 100
- Symptom domain: SS = (RS-1) /3 × 100.

Criteria scores for each domain were summarised by visit, observations and their change from baseline were tabulated (number of cases, mean, SD, median, minimum and maximum), and criterion scores for each domain were calculated as increasing (change from baseline ≥ 10 points), decreasing (change from baseline ≤ -10 points) and remaining stable (change from baseline between -10 and 10 points, excluding borders). The number of cases and percentage of subjects whose

Repeated-measures linear mixed-effects models (MMRM) were used to compare differences in total health status domain scores between the two treatment groups. The change from baseline in standardised scores at each time point after baseline was used as the dependent variable, with treatment group, random stratification factors, time point, and the interaction between treatment group and time point as independent variables, the corresponding baseline scores as covariates, and the within-subjects variance-covariance structure as an unstructured variance structure (UN). If the model did not converge when the covariance structure was UN, a covariance structure of CS could be chosen for the final model. Least squares means, standard errors of change from baseline at each time point after baseline, p-values for comparison of the test group relative to the control group, and the difference in change from baseline between the two groups (test-control), standard errors, and 95% two-sided CIs are reported for each treatment group.

- - 1. EORTC QLQ-LC13 Scores

EORTC QLQ-LC13 has a total of 13 entries, which can be categorised into 10 domains, and the specific raw scores (RS) for each domain are calculated as follows:

- Shortness of breath (LCDY) = (Q33+Q34+Q35)/3
- Cough (LCCO) = Q31
- Haemoptysis (LCHA) = Q32
- Oral pain (LCSM) = Q36
- Dysphagia (LCDS) = Q37
- Tingling in hands and feet (LCPN) = Q38
- Hair loss (LCHR) = Q39
- Chest pain (LCPC) = Q40
- Pain in arm or shoulder (LCPA) = Q41
- Pain in other areas (LCPO) = Q42

If missing data are encountered during the calculation of a raw score, the average of other non-missing questions may be used in place of the missing data, as long as the number of missing questions does not exceed half of the total number of questions required for that raw score. If the number of missing questions exceeds half of the total number of questions required for the raw score, the raw score is set as missing.

The raw scores were further converted linearly to standardised scores (SS) within 0-100 according to the following formula:

- SS = (RS-1) /3 × 100.

EORTC QLQ-LC13 was analysed using statistical methods similar to those used for the EORTC QLQ-C30 analysis.

1. Planned Analyses
   1. Interim Analysis

An interim analysis will be performed during the course of the trial when the number of 3/5 OS events (222) has been reached. The interim analysis will be performed by the Independent Data Monitoring Committee (IDMC) and the primary purpose of the interim analysis will be to assess whether the effectiveness endpoint has been reached earlier.

- The O'Brien Fleming one class error consumption function will be used to determine the one class error consumption based on the actual number of OS events that occur. It is expected that the one class error consumption at the time of the interim analysis will be approximately 0.0038 on one side.
- If the validity test for the primary endpoint has exceeded the O'Brien Fleming boundaries at the time of the interim analysis, it will be up to the IDMC to decide whether to recommend early termination of the trial due to superiority of validity.
- The Class I error boundary for the final analysis is approximately 0.0238 unilaterally, and the Class I error boundary for the final analysis will be adjusted appropriately if the final number of events or the number of events in the interim analysis varies slightly from the plan (which may be caused by multiple events occurring on the same day that the number of events is reached).
  1. Final Analysis

The trial will perform an OS master analysis when 369 OS events are reached.

1. Protocol Deviation

The following changes have been made to this statistical analysis plan relative to the analysis planned in the programme:

- The protocol defines DCR as "the percentage of patients with the best overall outcome of CR, PR, and SD that is maintained for more than 4 weeks." In the case of SD, measurements must have met the SD criteria at least once after study entry at a minimum interval (in general not less than 6-8 weeks), in accordance with RECIST 1.1, which generally requires that SD last for a minimum of 6 weeks ("In the case of SD, measurements must have met the SD criteria at least once after study entry at a minimum interval (in general not less than 6-8 weeks) that is defined in the study protocol."), the minimum duration of SD was therefore changed to 6 weeks; also as with ORR, the DCR will be calculated based on FAS .
- The protocol description for the quality of life scores "compares differences in overall survival domain scores between the two groups by analysis of covariance." Considering that the quality of life scores were assessed multiple times, to reflect the correlation between multiple scores for the same subjects, MMRM was used for the analyses.

1. Statistical analysis tables/lists/charts

Templates for statistical analysis tables/lists/charts will be provided separately in a separate document.

1. References

[1] Guideline on Statistical Design of Cancer Clinical Trials.

[2] New response evaluation criteria in solid tumours: Revised RECIST guideline (version 1.1).
