# Supplementary material for: A multicenter, randomized, double-blind, placebo-controlled phase 3 study of Socazolimab or placebo combined with carboplatin and etoposide in the first-line treatment of extensive-stage small cell lung cancer
Source: Signal Transduct Target Ther. 2025 Jan 13;10:28. doi: 10.1038/s41392-024-02115-5 (PMC11725569; doi:10.1038/s41392-024-02115-5)
Supplement: Supplementary file 2 — protocol [file 41392_2024_2115_MOESM2_ESM.docx]

**Protocol**

**A Randomized, Double-Blind, Placebo-Controlled, Multi-center Phase III Clinical Trial of Recombinant Fully Human Anti-PD-L1 Monoclonal Antibody Socazolimab (ZKAB001) Combined with Carboplatin and Etoposide for the Treatment of Extensive Stage Small Cell Lung Cancer**

**Protocol Number:** **ZKAB001-LEES-2020-07**

**Version Number:** 1.2

**Protocol Date:** 2022.04.27

**Zhaoke (Guangzhou) Oncology Pharmaceutical Ltd.**

Proprietary Statement: Zhaoke (Guangzhou) Oncology Pharmaceutical Ltd own the rights to the contents of this document.

It should not be disclosed to others without written permission.

**TABLE OF CONTENTS**

[LIST OF ABBREVIATIONS 6](#_Toc169248316)

[ABSTRACT 7](#_Toc169248317)

[1. INTRODUCTION 17](#_Toc169248318)

[1.1 Small Cell Lung Cancer (SCLC) 17](#_Toc169248319)

[1.2 The Treatment of SCLC 17](#_Toc169248320)

[1.3 Use of Immune Checkpoint Inhibitors in The Treatment of SCLC 18](#_Toc169248321)

[1.4 Use of Immune Checkpoint Inhibitors Combined with Chemotherapy in The Treatment of SCLC 18](#_Toc169248322)

[1.5 Recombinant Humanized PD-L1 Monoclonal Antibody Injection (ZKAB001) 20](#_Toc169248323)

[2. OBJECTIVES AND ENDPOINTS 22](#_Toc169248324)

[2.1 Primary Objective 22](#_Toc169248325)

[2.2 Secondary Objectives 22](#_Toc169248326)

[2.3 Endpoints 22](#_Toc169248327)

[2.3.1 Primary Endpoints 22](#_Toc169248328)

[2.3.2 Secondary Endpoints 22](#_Toc169248329)

[3. STUDY DESIGN 23](#_Toc169248330)

[3.1 Overall Design 23](#_Toc169248331)

[3.2 Sample Size Determination 24](#_Toc169248332)

[4. RESEARCH POPULATION 24](#_Toc169248333)

[4.1 Indication 24](#_Toc169248334)

[4.2 Inclusion Criteria 25](#_Toc169248335)

[4.3 Exclusion Criteria 26](#_Toc169248336)

[4.4 Criteria of Termination of Treatment 28](#_Toc169248337)

[4.5 Criteria of Withdrawal from the Study 29](#_Toc169248338)

[5. SUBJECT RECRUITMENT 29](#_Toc169248339)

[6. DOSAGE AND DOSING SCHEDULE 30](#_Toc169248340)

[6.1 Packaging and Labeling 30](#_Toc169248341)

[6.2 Study Drugs 30](#_Toc169248342)

[6.3 Drug Accountability 30](#_Toc169248343)

[6.3.1 Drug Supplying and Receiving 30](#_Toc169248344)

[6.3.2 Drug Management 31](#_Toc169248345)

[6.3.3 Drug Storage 31](#_Toc169248346)

[6.3.4 Preparation and Disposal of Study Drugs 31](#_Toc169248347)

[6.3.5 Drug Count 32](#_Toc169248348)

[6.3.6 Return and Destruction of Study Drug 33](#_Toc169248349)

[6.4 Study Drug Administration 33](#_Toc169248350)

[6.4.1 Pretreatment 33](#_Toc169248351)

[6.4.2 Administration of ZKAB001 and Placebo 33](#_Toc169248352)

[6.4.3 EC Administration Schedule 34](#_Toc169248353)

[6.4.4 Sequence of Administration of Study Drugs 34](#_Toc169248354)

[6.5 Dose Adjustment and Discontinuation 35](#_Toc169248355)

[6.5.1 Dose Adjustment and Discontinuation of ZKAB001 35](#_Toc169248356)

[6.5.2 Dose Adjustment and Discontinuation of EC combinations 37](#_Toc169248357)

[7. CONCOMITANT TREATMENTS 40](#_Toc169248358)

[7.1 Contraindicated Drugs and Treatments During the Study 40](#_Toc169248359)

[7.2 Permitted Drugs and Treatments During the Study 40](#_Toc169248360)

[8. STUDY PROCEDURES 42](#_Toc169248361)

[8.1 Informed Consent 42](#_Toc169248362)

[8.2 Treatment Assignment and Blinding 43](#_Toc169248363)

[8.2.1 Identification of Codes and Blinding 43](#_Toc169248364)

[8.2.2 Randomization 43](#_Toc169248365)

[8.2.3 Compliance Control 44](#_Toc169248366)

[8.2.4 Unblinding 44](#_Toc169248367)

[8.3 Independent Radiology Evaluation 45](#_Toc169248368)

[8.4. Data Monitoring Committee 45](#_Toc169248369)

[8.5 Observation Items 45](#_Toc169248370)

[8.6 Examinations at Screening Period and Baseline 48](#_Toc169248371)

[8.7 Treatment and Follow-Up Examinations 49](#_Toc169248372)

[9. CLINICAL EVALUATIONS 51](#_Toc169248373)

[9.1 Efficacy Evaluation 51](#_Toc169248374)

[9.2 Safety Evaluation 53](#_Toc169248375)

[9.3 Immunogenicity Analysis 53](#_Toc169248376)

[9.3.1 Time of Blood Sampling 53](#_Toc169248377)

[9.3.2. Processing and Preservation of Blood Samples 53](#_Toc169248378)

[9.3.3 Transportation of Blood Samples 54](#_Toc169248379)

[9.3.4 Testing of Blood Samples 54](#_Toc169248380)

[10. REPORTING OF ADVERSE EVENTS 54](#_Toc169248381)

[10.1 Adverse Event (AE) 54](#_Toc169248382)

[10.2 Serious Adverse Event (SAE) 54](#_Toc169248383)

[10.3 Adverse Event/Serious Adverse Event Relationship Assessment 56](#_Toc169248384)

[10.4 Evaluation of Severity of Adverse Events 57](#_Toc169248385)

[10.5 Suspected, Unexpected Serious Adverse Reactions（SUSAR） 57](#_Toc169248386)

[10.6 Reporting of Serious Adverse Events 58](#_Toc169248387)

[10.7 Follow-up of Adverse Events 60](#_Toc169248388)

[11. DATA MANAGEMENT 61](#_Toc169248389)

[11.1 Data Management Tools 61](#_Toc169248390)

[11.2 Electronic Case Report Form (eCRF) Construction 61](#_Toc169248391)

[11.3 Privilege Management 61](#_Toc169248392)

[11.4 Data Input 61](#_Toc169248393)

[11.5 Data Questions and Answers 62](#_Toc169248394)

[11.6 Data Locking and Output 62](#_Toc169248395)

[11.7 Provisions for Source Data 62](#_Toc169248396)

[12. STATISTICAL ANALYSIS 63](#_Toc169248397)

[12.1 General Considerations 63](#_Toc169248398)

[12.2 Analytic Populations 64](#_Toc169248399)

[12.3 Analysis of Efficacy 64](#_Toc169248400)

[12.4 Analysis of Safety 65](#_Toc169248401)

[12.5 Interim Analysis 65](#_Toc169248402)

[13. QUALITY CONTROL AND QUALITY ASSURANCE 65](#_Toc169248403)

[14. ETHICAL PRINCIPLES 66](#_Toc169248404)

[14.1 General Considerations 66](#_Toc169248405)

[14.2 Informed Consent Process and Signing of Informed Consent Form 66](#_Toc169248406)

[15. DATA REETENTION AND SUMMARIZATION 67](#_Toc169248407)

[16. RESPONSIBILITIES OF ALL PARTIES AND REGULATIONS ON PUBLICATIONS 67](#_Toc169248408)

[REFERENCES 69](#_Toc169248409)

[Appendix 1: Method of Staging Small Cell Lung Cancer 71](#_Toc169248410)

[Appendix 2: ECOG Performance Status Evaluation 72](#_Toc169248411)

[Appendix 3: Response Evaluation Criteria in Solid Tumors (RECIST1.1) (Revision) 73](#_Toc169248412)

[Appendix 4: Management of Immune Checkpoint Inhibitor-Related Toxicity 77](#_Toc169248413)

[Appendix 5: Study Diagram 83](#_Toc169248414)

[Appendix 6: EORTC QLQ-C30+LC13 Quality of Life Score 88](#_Toc169248415)

[Appendix 7: Body Surface Area Calculation 92](#_Toc169248416)

# LIST OF ABBREVIATIONS

| ADCC | Antibody-Dependent Cell-Mediated Cytotoxicity |
| --- | --- |
| ADR | Adverse Drug Reaction |
| AJCC | American Joint Committee on Cancer |
| AE | Adverse Event |
| AUC | Area Under Curve |
| BSC | Best Supportive Care |
| CR | Complete response |
| CRF | Case Report Form |
| CSR | Clinical Study Report |
| CT | Computed Tomography |
| CTCAE | Common Terminology Criteria for Adverse Events |
| CTLA-4 | Cytotoxic T Lymphocyte-Associated Antigen-4 |
| DCR | Disease Control Rate |
| DLT | Dose Limiting Toxicity |
| DOR | Duration of Response |
| DNA | Deoxyribonucleic Acid |
| ECG | Electrocardiography |
| EDC | Electronic Data Capture |
| ES-SCLC | Extensive Stage Small Cell Lung Cancer |
| GCP | Good Clinical Practice |
| HCG | Human Chorionic Gonadotropin |
| ICF | Informed Consent Form |
| ILD | Interstitial lung Disease |
| MRI | Magnetic Resonance Imaging |
| MTD | Maximal Tolerance Dose |
| ORR | Overall Response Rate |
| PD | Progressive Disease |
| PD-1 | Programmed Cell Death 1 |
| PD-L1 | Programmed Cell Death Ligand 1 |
| PS | Performance Score |
| PK | Pharmacokinetics |
| PR | Partial Response |
| RECIST | Response Evaluation Criteria in Solid Tumors |
| RNA | Ribonucleic Acid |
| SAE | Serious Adverse Event |
| SUSAR | Suspicious Unexpected Serious Adverse Reactions |
| t_1/2_ | Half-Life |
| ULN | Upper Limit of Normal |
| VALG | Veterans Administration Lung Study Group |
| VEGF | Vascular Endothelial Growth Factor |
| VEGFR | Vascular Endothelial Growth Factor Receptor |

# ABSTRACT

| **Study Title** | A Randomized, Double-Blind, Placebo-Controlled, Multi-center Phase III Clinical Trial of Recombinant Fully Human Anti-PD-L1 Monoclonal Antibody Socazolimab (ZKAB001) Combined with Carboplatin and Etoposide for the Treatment of Extensive Stage Small Cell Lung Cancer |
| --- | --- |
| **Indication** | Extensive-stage small cell lung cancer (ES-SCLC) without prior systemic treatment. |
| **Objectives** | **Primary Objectiv**e   - To evaluate the overall survival (OS) of ZKAB001 combined with carboplatin and etoposide compared with placebo combined with carboplatin and etoposide as first-line treatment of ES-SCLC.   **Secondary Objectives**   - To evaluate the progression-free survival (PFS), objective response rate (ORR), disease control rate (DCR), duration of response (DOR), and OS rate at 1 year and 2 years for ZKAB001 combined with carboplatin and etoposide versus placebo combined with carboplatin and etoposide as first-line treatment of ES-SCLC; - To evaluate the safety of treatment; - To evaluate the relationship between PD-L1 expression status and clinical effects - To evaluate the immunogenicity of ZKAB001 - To evaluate quality of life before and after treatment. |
| **Endpoints** | **Primary Endpoints**   - Overall survival (OS)   **Secondary Endpoints**   - PFS assessed by investigators and IRC (based on RECIST v1.1) - ORR assessed by investigators and IRC (based on RECIST v1.1) - DoR assessed by investigators - DCR assessed by investigators - OS rate at 1 year and 2 years - Incidence and severity of adverse events and severe adverse events, and abnormal laboratory measures (based on CTCAE 5.0) - PD-L1 expression status in tumor tissue - The positive rate of drug-resistant antibodies (ADA) - Quality of life score (EORTC QLQ-C30 and QLQ-LC13) |
| **Study Design** | This study is a randomized, double-blind, placebo-controlled, multicenter Phase III study. Eligible patients will be randomly assigned to the experimental group: ZKAB001+ carboplatin + etoposide, or the control group: placebo + carboplatin + etoposide at a ratio of 1:1, with every 3 weeks as one treatment cycle. Stratification factors included gender (male/female), PS (0/1), and brain metastases (yes/no).  The study is consisted of a screening period, a treatment period (subjects will receive study treatment until confirmed disease progression, or intolerable toxic effect, or the maximum duration of 2 years, or subjects voluntarily requested termination of study treatment), and a follow-up period (including both safety and survival follow-up).  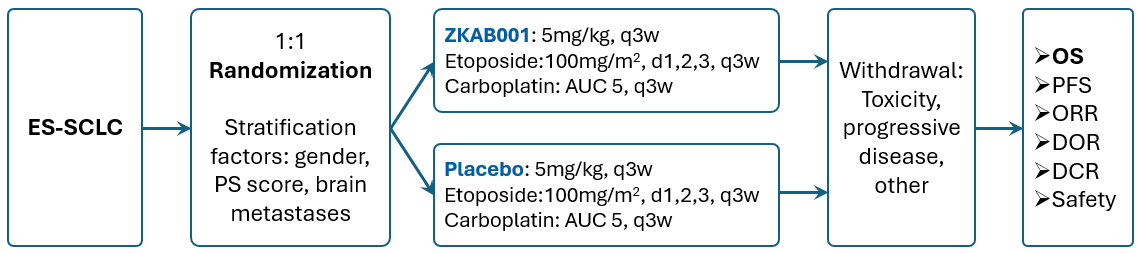  **Figure1. Study Design** |
| **Duration of Trial** | 24 months (estimated) |
| **Criteria of Subjects Selection** | **Inclusion Criteria**   1. Male or female, age ≥ 18 years. 2. Small Cell Lung Cancer confirmed by histology. 3. Extensive-stage SCLC (defined as AJCC 8th Edition IV (Tany, Nany, M1a/b/c), or T3-4 SCLC that cannot be included in a tolerable radiotherapy plan due to multiple pulmonary nodules or tumor/nodule size). 4. Have not received first-line systemic treatment for ES-SCLC in the past. 5. Have received surgery and adjuvant therapy for cure, such as radiotherapy and chemotherapy, and there was an interval of at least 6 months from the last chemotherapy or radiotherapy to the diagnosis of ES-SCLC. 6. ECOG performance status of 0-1. 7. Have a life expectancy ≥ 8 weeks. 8. Have at least one measurable lesion (according to CT or MRI scan, based on RECIST v1.1) within 28 days before administration of the first dose. 9. Partners of male subjects and female subjects with childbearing potential must be willing to conduct effective contraception from signing the informed consent till 6 months after the last dose of study drug. And the human chorionic gonadotropin in blood / urine (HCG) test of female subjects with childbearing potential must be negative 7 days before the first dose. 10. Adequate hematologic, liver, kidney and coagulation function indicated by the following laboratory values:  - Blood routine test (without blood transfusion and hematopoietic factor drugs within 14 days before screening): White blood cell count ((WBC) ≥ 3.0x10^9); absolute neutrophil count ((ANC)) ≥ 1.5x10^9; platelet (PLT) ≥ 100x10^9; hemoglobin content ((hGB) ≥ 90g). - Liver function: aspartate aminotransferase (AST) ≤ 2.5 ULN, alanine liver aminotransferase (ALT) ≤ 2.5 ULN; ALT and AST < 5 ULN; serum total bilirubin (TBIL) ≤ 1.5 ULN; albumin (ALB) ≥ 30 g L; - Renal function: serum creatinine ≤ 1.5 ULN or creatinine clearance rate (Ccr) ≥ 40 mL/min (Cockcroft/Gault formula). - Coagulation function: international standardized ratio (INR) ≤ 1.5 ULN, activated partial thromboplastin time (APTT) ≤ 1.5 ULN; - Alkaline phosphatase (ALP) ≤ 2.5 ULN, bone metastasis subjects, ALP ≤ 5 ULN.  1. Able to provide tumor tissue samples that can meet the requirements of PD-L1 expression detection from screening to 4 weeks after enrollment. 2. Voluntarily participation through written informed consent，with good compliance and cooperation with follow-up.   **Exclusion Criteria**   1. Have received any T cell costimulatory or immune checkpoint inhibitors, including, but not limited to, cytotoxic T lymphocyte associated antigen-4 (CTLA-4) inhibitors, PD-1 inhibitors, PD-L1/2 inhibitors or other drugs targeting T cells; anti-vascular endothelial growth factor (VEGF) or vascular endothelial growth factor receptor (VEGFR) therapy. 2. Active brain metastasis or meningeal metastasis. Patients with brain metastasis after treatment need to meet the following criteria to be enrolled: asymptomatic; no imaging evidence of progress for at least 4 weeks after treatment; completion of treatment for at least 7 days before the first dose of the study drug; do not need to receive systemic corticosteroids (> 10mg/ prednisone or equivalent) for 14 days before the first dose of the study drug. If a new asymptomatic brain metastasis is found during the screening period, radiotherapy and/or surgery will be required. If all other criteria are met after treatment, additional brain scans are not required before randomization. 3. Radiotherapy: the completion of radiotherapy for the brain or palliative radiotherapy for the focus of bone lesion is within 7 days before the first dose of the study drug. 4. Active, known or suspected autoimmune diseases, including, but not limited to, myasthenia gravis, myositis, autoimmune hepatitis, systemic lupus erythematosus, rheumatoid arthritis, inflammatory bowel disease, vascular thrombosis associated with antiphospholipid syndrome, Wegener's granuloma, Sjogren's syndrome, Guillain-Barre syndrome, multiple sclerosis, vasculitis or glomerulonephritis. Cases that can be included: residual hypothyroidism due to autoimmune thyroiditis that require hormone replacement therapy, well-controlled type I diabetes, or no recurrence expected in the absence of external stimulation; eczema, psoriasis, neurodermatitis or vitiligo (psoriatic arthritis patients will be excluded) with only topical presentation can be included if they meet the following criteria: the area covered by the rash must be less than 10% of the body surface area; the disease is well controlled at the baseline level, requiring only inefficient topical steroids, and with no acute exacerbation in the past 12 months. 5. Uncontrolled pleural effusion, pericardial effusion or ascites requiring repeated drainage (once a month or more frequently). Patients who use indwelling catheters are allowed to be included. 6. Corticosteroids (> 10 mg/ prednisone or equivalent dose) or other immunosuppressants were used within 14 days before the first dose. Inhalation or topical use of steroids and adrenal replacement steroids are allowed in the absence of active autoimmune disease; for patients receiving short-term, systemic immunosuppressive therapy, for example, glucocorticoids for nausea, vomiting, or allergic reaction management or preventive use can be enrolled after consultation with the sponsor. The use of mineralocorticoid in the treatment of postural hypotension and the use of low-dose glucocorticoid supplements in the treatment of adrenocortical insufficiency is allowed. 7. Patients who had been vaccinated within 4 weeks before first dose or planned to receive live vaccines. 8. Major surgery was performed within 4 weeks before first dose of study drug, or major surgery was scheduled during the study period. 9. Interstitial pneumonia (ILD) disease, drug-induced pneumonia, radiation pneumonia requiring steroid treatment or active pneumonia with clinical symptoms. 10. Active pulmonary tuberculosis or history of active pulmonary tuberculosis infection within 1 year before screening, whether treated or not. 11. Uncontrolled cardiovascular diseases, such as: (1) heart failure (New York Heart Association (NYHA) grade 2 or above) (2) unstable angina pectoris (3) myocardial infarction or cerebrovascular accident within 6 months (4) clinically significant supraventricular or ventricular arrhythmias that needs to be treated. 12. Uncontrolled active infections (e.g. those who need intravenous antibiotics, antifungal or antiviral therapy). 13. Active hepatitis B or C (HBV-DNA titer < 500IU/mL or copy number < 1000copies/ml, HCV-RNA negative after antiviral treatment can be included), HIV positive or known history of acquired immunodeficiency syndrome. 14. Known allergies to study drugs or excipients; known severe allergic reactions to any monoclonal antibody; allergic history of carboplatin or etoposide. 15. Patients who have previously received allogeneic bone marrow transplantation or solid organ transplantation. 16. Other malignancy diagnosed less than 5 years before the first dose, except for fully treated cervical carcinoma in situ, basal cell or squamous cell skin cancer, local prostate cancer after radical resection, and ductal carcinoma in situ after radical mastectomy. 17. Have been treated with any other experimental drugs or participated in another interventional clinical study within 4 weeks before signing ICF. 18. Pregnant or lactating women. 19. Known cases of mental illness, alcohol abuse, inability to quit smoking, drug use or substance abuse. 20. Other situations assessed by the investigators to be unsuitable for participation in this study.   **Criteria of Withdrawal from the Study**  1) The subjects are unwilling to continue to participate in the study and refuse further follow-up;  2) Any clinical adverse events, laboratory abnormalities, or co-morbidities making the investigator believe that continuing participation in the study is not in the subject's best interest;  3) Receiving new anti-tumor therapy;  4) Other circumstances in which the investigator considers it necessary to withdraw from the study;  5) Loss of follow-up  6) Death  7) The sponsor terminated the study |
| **Study Drug** | **Socazalimab (ZKAB001) / placebo**  Strength: 100 mg/4 mL/vial  Drug supplier: Zhaoke (Guangzhou) Oncology Pharmaceutical Ltd.  **Carboplatin injection**  Information seen drug labels.  **Etoposide injection**  Information seen drug labels. |
| **Dosage** | 1. Experimental group: Socazolimab + EC 2. Placebo group: placebo + EC   **Socazolimab / placebo**  5 mg/kg (intravenous injection) for 21 days (3 weeks) as a treatment cycle. Until an event occurs that meets the criteria for withdrawal from the trial.  **Carboplatin**  AUC5, intravenous injection, for 21 days (3 weeks) as a treatment cycle.  **Etoposide**  Continuous administration on day1, day 2, and day 3, 100mg/m^2^ (intravenous injection), 21 days (3 weeks) as a treatment cycle. |
| **Sample Size** | In this study, OS was the primary endpoint, and the superiority test was performed with the control group. The parameters for calculating the sample size are as follows:  Efficacy hypothesis:  • Median OS (month) = 10.5 months (control group), HR=0.73;  • Random ratio: 1:1;  • Enrollment and follow-up time: enrollment period of 18 months + follow-up period of 18 months;  • Annual dropout rate: 5%;  We plan to do an interim analysis at 3/5 event count (60% data maturity), using O 'Brien Fleming Type 1 error consumption function. With 85% confidence in the OS test, a total of 369 OS events and approximately **498** subjects would be required to meet the target event. |
| **Study Procedure** | **Screening period**: Participants are required to undergo screening tests or assessments (some of which may be based on local results prior to signing the informed consent form, see study schedule) within 28 days prior to first dose to determine whether they meet the inclusion criteria and do not meet the exclusion criteria. Tumor samples (wax blocks of archived or freshly obtained tumor tissue or pathological biopsy sections within 6 months before first dose) will be collected from subjects during screening until 4 weeks after enrollment.  **Treatment period**: Study drugs will be given in a specified order at the first day of each cycle (every 3 weeks (-3 ~ +7 days) as 1 cycle). During the first 4 cycles, subjects will receive experimental drug/placebo combined chemotherapy, followed with maintenance therapy of experimental drug/placebo only from the 5^th^ cycle. Within 7 days prior to each dose, subjects must complete imaging examination, vital signs, physical examination, laboratory examination, physical status score, etc. Imaging examinations will be performed every 6 weeks ±7 days until confirmed disease progression or death, loss of follow-up, subjects' unwillingness to continue in the trial, or initiation of new tumor therapy.  **Safety follow-up period**: Within 90 days after the last dose of medication, safety follow-up should be conducted every 30 days ±7 days, and patients should return to the hospital for first evaluation as far as possible or conduct examination in a local hospital. If it is confirmed that more than one cycle (3 weeks) has passed since the last dose of medication at the time of withdrawal from the study, the end-of-trial examination should be the first safety follow-up after the end of medication. Information on survival status, adverse events, concomitant medication, and concomitant therapy can be collected at two subsequent telephone follow-up visits, until death, loss of follow-up, initiation of new anti-tumor therapy, or completion of all three safety follow-up visits.  **Survival follow-up**: All subjects will be followed up by telephone every 30 days after termination of treatment and safety follow-up, and survival status will be collected until death, loss of follow-up, or trial termination. |
| **Statistical Analysis** | **Analysis of Efficacy**  The efficacy analysis will be based on FAS and PPS, with FAS as the primary and PPS as the secondary.  1）Primary efficacy  The primary endpoint of this study is OS. the Kaplan-Meier method was used to estimate the median OS and its corresponding confidence interval, to estimate the survival rate of OS at different time points (including but not limited to the OS rate at 1 year and 2 years), and to plot the corresponding survival curves. A stratified Log-Rank test will be used to compare OS between the two groups, with stratification factors considered as for randomized stratification factors. In addition, a COX proportional wind model will be used to estimate the hazard ratio (HR) of the test group relative to the control group and its corresponding confidence interval. The treatment group and randomization stratification factors will be considered as independent variables in the model.  2）Secondary efficacy  For the secondary endpoint objective remission rate (ORR), the number and proportion of subjects summarizing ORR by treatment group and their 95% confidence interval (based on the Clopper-Person method). A stratified Cochran-Mantel-Haenszel (CMH) method was used to compare the difference in ORR between the test group and the control group, and P values were calculated. The difference in ORR rate between the two groups based on the normal distribution approximation and its 95% confidence interval. Disease control rate (DCR) will be analyzed in the same way as ORR. PFS and DoR will be analyzed using similar analytical methods as for the primary study endpoint OS. For quality of life scores, descriptive statistics of the total and dimensional scores at baseline and at each evaluation time point, as well as the change values relative to baseline, will be used to compare the differences in overall survival domain scores between the two groups by analysis of covariance.  **Analysis of Safety**  Treatment Emergent Adverse Event (TEAE) will be coded using the MedDRA dictionary. The incidence of TEAEs will be summarized separately by treatment group according to system organ class and preferred terminology. The incidence of all TEAEs, adverse reactions, TEAEs leading to discontinuation and termination of therapy, TEAEs leading to death, and SAEs will be summarized. The severity and relevance to treatment of TEAEs were also further summarized according to the most severe criterion. Descriptive statistics were used to summarize other safety indicators. Baseline was defined as data from the most recent test prior to the first dose. Laboratory tests: descriptive summaries of laboratory test values and analysis of post-treatment abnormalities in the form of pre- and post-treatment cross-tabulations. Vital signs: Mean, maximum, minimum, median, and standard deviation were used to describe measurements and changes at each visit. Physical examination and lead ECG were analyzed descriptively. |

# 1. INTRODUCTION

## 1.1 Small Cell Lung Cancer (SCLC)

Small cell lung cancer (SCLC) accounts for about 14% of lung cancer [1,2], and is the most aggressive subtype of lung cancer. The most common site of SCLC is bronchial centered spread, and only 5% is isolated small peripheral lesions. Most patients were in the progressive stage and easy to metastasize early. The growth was rapid, and the doubling time (TD) was 75.9 days. 90% of SCLC was diagnosed with intra thoracic organ and tissue invasion, lymphatic tract and distant metastasis. About 30-40% of SCLC are limited stage, and 60-70% are extensive stage. The prognosis is poor, the average natural survival is only 3-6 months, and the average 5-year survival rate is about 5% [3].

SCLC usually presents as a large hilar mass and enlarged mediastinal lymph nodes, which can cause coughing and dyspnea. Patients often present with symptoms of widespread metastatic disease, such as weight loss, weakness, bone pain, and nerve damage. Many neurological and endocrine paraneoplastic syndromes are associated with SCLC neurological syndromes, including Lambert-Eaton myasthenia syndrome (LEMS), encephalomyelitis, and sensory neuropathy. SCLC cells sometimes produce polypeptide hormones, including antidiuretic hormone (ADH) and adrenocorticotropin (ACTH), which can lead to malignant hyponatremia (i.e., improper ADH secretion syndrome) and Cushing's syndrome, respectively.

The staging of SCLC has followed the two-stage staging method of the American Veterans Lung Cancer Association (VALG) [4], mainly based on the importance of radiotherapy in the treatment of SCLC. AJCC TNM staging system is applicable for selecting patients with stage T1-2N0 suitable for surgery, and the TNM staging system can assess prognosis and guide treatment more accurately [3].

## 1.2 The Treatment of SCLC

According to the staging, T1-2N0 (stage I) patients without mediastinal lymph node metastasis are generally considered to undergo surgical resection [5]. However, only less than 5% of the patients were actually stage I [6]. Chemotherapy is essential for all patients with SCLC. For patients with limited-stage SCLC whose stage exceeds T1-2 and N0 and PS is good (0-2), the chemotherapy regimen of synchronous chest radiotherapy is generally adopted [7]. Platinum combined with etoposide (EP or EC regimen) is the classic first-line treatment for limited-stage SCLC. The EP/EC regimen was also used for patients with PS-3-4 that was clearly caused by SCLC. For patients with extensive stages, the preferred regimen includes etoposide or irinotecan combined with cisplatin or caplatin (EP/EC/IP/IC), in addition to local radiotherapy for patients with significant local symptoms, such as superior vena cava syndrome, spinal cord compression symptoms, bone metastases, and obstructive atelectasis. For patients with brain metastases, whole brain radiotherapy should be combined [8]. Among them, EP regimen (etoposide combined with cisplatin) is the most commonly used chemotherapy regimen for initial treatment [9]. Carboplatin is also commonly used to replace cisplatin in the clinic to reduce vomiting, neurotoxicity and nephrotoxicity, but at the same time increases the risk of myelosuppression. A meta-analysis of 663 patients showed no difference between cisplatin and carboplatin in response rate (67% vs. 66%), PFS (5.5 vs. 5.3m), or OS (9.6 vs. 9.4m) for either limited-stage (32%) or extensive-stage (68%) [10]. In addition, maintenance or consolidation chemotherapy normally does not exceed 4-6 cycles, and extended chemotherapy only results in a slight extension of remission time without improving survival and increases the risk of cumulative toxicity [11].

Most SCLC patients will experience relapse and drug resistance after first-line treatment [12]. The median survival of these patients is only four to five months. Although the likelihood of remission largely depends on the time from initial treatment to relapse, many patients achieve significant remission on subsequent systemic therapy [13]. Most drugs or regimens have very poor efficacy (≤10%) if the duration of response is less than 3 months (refractory or resistant disease), the expected response rate will be about 25%. If a patient relapses more than 6 months after first-line therapy, the original treatment regimen is recommended.

## 1.3 Use of Immune Checkpoint Inhibitors in The Treatment of SCLC

In recent years, tumor immunotherapy, such as programmed cell death-1 (PD-1)/programmed cell ligand-1 (PD-L1) inhibitors, has become popular in the field of tumor treatment. Based on the results of CheckMate 032 trial, nivolumab with or without ipilimumab are approved for second-line treatment of SCLC. In 2017, ASCO reported the follow-up results of this study [14], the objective response rate (ORR) of the combination treatment group was 23%, the 2-year OS rate was 26%, and the median OS was 7.8 months; in the monotherapy group, the ORR was 11%, the 2-year OS rate was 14%, and the median OS was 4.1 months. CheckMate 032 study only compared the efficacy of different immunotherapy regimens for relapsed resistant SCLC and did not compare them with current standard second-line chemotherapy regimens.

## 1.4 Use of Immune Checkpoint Inhibitors Combined with Chemotherapy in The Treatment of SCLC

It is traditionally believed that chemotherapy leads to myelosuppression and thus kills immune cells against tumor immune response. However, in recent years, more and more evidence has shown that most chemotherapy drugs play a positive role in anti-tumor immune response, including immunogenicity, antigen presentation, inhibitory cells and effector response to tumor cells [15]. Cytotoxic drugs that damage DNA can activate immunogenic cell death, change tumor inflammatory microenvironment and stimulate the generation of neoantigens, thus activating anti-tumor immunity [16]. Immunogenic chemotherapy drugs (such as oxaliplatin and cyclophosphamide) can activate anti-tumor T cell immune response [17], and directly attack tumor cells to stimulate anti-tumor immune response, which depends on signaling of toll-like receptor 4 and anti-tumor immunity of CD8+T cells. This provides a theoretical basis for the combination of chemotherapeutic drugs with immune checkpoint inhibitors. Chemotherapy can also induce the production of neoantigens and increase tumor mutation, thus stimulating the immune response of T cells and enhancing the sensitivity of immune checkpoint inhibitors [18]. Remodeling the immune microenvironment, based on the tumor response to immune checkpoint inhibitors, some tumor microenvironments have many tumor-specific T cells or immune killer cells infiltrated the tumor. These tumors have a persistent immune response to programmed PD-1 inhibitors. In contrast, some tumors have little microenvironment for tumor-specific T cells or immune killer cells to infiltrate. The combination of chemotherapy and immune checkpoint inhibitors can increase such cell infiltration, resulting in a significant anti-tumor immune response by increasing the expression of tumor-specific lymphocytes and interferon-γ-induced genes in the microenvironment, thereby increasing the expression of PD-L1 [18].

Based on the above principles, the clinical trials of the combination of chemotherapy drugs and immune checkpoint inhibitors are entering a new period.

In the recent global phase I/III double-blind, randomized, placebo-controlled trial of Atezolizumab in ES-SCLC, 403 patients with extensive stage SCLC were randomly assigned to receive Atezolizumab + EC regimen for 4 cycles of induction chemotherapy and sequential Atezolizumab maintenance therapy (201 cases) or EC regimen plus placebo therapy and sequential placebo maintenance therapy (202 cases) and follow-up until disease progression or intolerable adverse events or no longer clinically beneficial. The results showed that Atezolizumab significantly prolonged OS at a median follow-up of 13.9 months (median OS, 12.3 m vs. 10.3 m, HR=0.70, 95%CI 0.54 to 0.91, P=0.007). The median PFS of the two groups were 5.2 m and 4.3 m, respectively (HR=0.77, 95%CI 0.62~0.96, P=0.02).

Another similar study, the CASPIAN Study, was designed to explore the efficacy of the anti-PD-L1 antibody Durvalumab and the anti-CTLA-4 antibody Tremelimumab in combination with chemotherapy in first-line treatment of patients with ES-SCLC. Patients were randomly assigned into three groups to receive Durvalumab ± Tremelimumab (CTLA4 inhibitor) combined with EP regimen (etoposide + cisplatin/carboplatin). In the Durvalumab group, patients received up to four cycles of chemotherapy, while the chemotherapy group allowed up to six cycles of chemotherapy and prophylactic intracranial irradiation. In the CASPIAN study, 268 patients were enrolled in the Durvalumab group and 269 in the chemotherapy group. OS in the Durvalumab combined chemotherapy group was 13.0 months compared with 10.3 months in the chemotherapy group (HR=0.73, p=0.0047). The safety and tolerability of the Durvalumab combination chemotherapy regimen are consistent with the known safety profile of the drug, indicating that the regimen is safe and feasible.

Based on the results of these two studies, both Atezolizumab and Durvalumab have been approved for first-line treatment of ES-SCLC.

## 1.5 Recombinant Humanized PD-L1 Monoclonal Antibody Injection (ZKAB001)

ZKAB001 was selected from the world's largest human G-MAB^TM^ antibody library, and has the significantly high specificity and affinity against human PD-L1. By binding to PD-L1, the PD-1/PD-L1 signaling pathway is blocked, then suppressing inhibition of T cells induced by the PD-L1/PD-1 pathway. Finally, T cells are activated, thereby exerting the anti-tumor growth effect via enhancing T cell-mediated autoimmunity. ZKAB001 also has an intact IgG1 Fc fragment that can be recognized by Fc receptors on NK cells. Once the Fc receptor binds to the Fc region of the IgG, NK cells release cytokines (such as IFN-γ) and cytotoxic particles (including perforin and granase), resulting in antibody-dependent cell-mediated cytotoxic (ADCC) effects. ZKAB001 binds to PD-L1 on the surface of tumor cells and guides NK cells to adsorb to the tumor region through its Fc end, achieving the effect of ADCC on tumor inhibition. ZKAB001 is intended for the treatment of recurrent or metastatic solid tumors.

Currently completed or ongoing clinical trials of ZKAB001 covers different indications including small cell lung cancer, cervical cancer, osteosarcoma, biliary system malignancies, malignant melanoma, and urothelial tumors at a therapeutic dose of 5/10/15 mg/kg. As of March 31, 2021, a total of 250 patients received at least one dose of ZKAB001 or placebo and had post-administration safety data (estimated cumulative exposure to ZKAB001 was 209). TRAE was reported in 113 (56.8%) subjects of the 199 patients receiving only Socazolimab. The most common TRAEs were Hypothyroidism (34(17.1%)), decreased white blood cell count (22(11.1%)), elevated alanine aminotransferase (14(7.0%)), hyperthyroidism (13(6.5%)), and decreased neutrophil count (12(6.0%)). TRAE was reported in 29 (56.9%) subjects of the 51 patients receiving Socazolimab combined with chemotherapy. The most common TRAEs were decreased platelet count (7(13.7%)), decreased white blood cell count and hyperglycemia (6(11.8%)), anemia and neutrophil count (5(9.8%)).

In order to evaluate the safety of ZKAB001 combined with EC in patients with ES-SCLC, a single-arm, Phase Ib trial (NCT04346914) was conducted, with a total of 20 patients enrolled. From April 15, 2020 to December 30, 2010, 20 patients with extensive stage small cell lung cancer were treated with ZKAB001, carboplatin, and etoposide. The ORR was 70.0% (95%CI: 45.72%, 88.11%), the median PFS was 5.65 months (95%CI: 4.14, 6.54), and the median DOR was 4.29 months (95%CI: 2.76, 5.85). The median OS was 14.88 months (95%CI: 10.09, NE). 95% of patients experienced grade 3 or higher treatment-related adverse events. There were no treatment-related deaths.

All patients experienced one or more drug-related adverse events (TRAEs), almost all of which occurred during the combination treatment phase, and the incidence of severe (grade 3 or 4) AEs was 19/20 (95%), of which 5/20 (25%) were related to the Socazolimab and 19/20 (95%) were related to chemotherapy. The incidence of AEs leading to drug reduction (chemotherapy) was 15/20 (75%), 5/20 (25%) leading to drug suspension, 2/20 (10%) leading to carboplatin suspension, and 4/20 (25%) leading to etoposide suspension. 1 in 20(5%) resulted in drug discontinuation (all experimental drugs). Most hematological toxicity occurred during combination therapy. Remission was achieved after symptomatic treatment or chemotherapy reduction.

4/20 (20%) patients had infusion reaction during treatment, with 1 patient withdrew from the study due to a grade 2 infusion reaction that recurred after preconditioning. All the others were relieved after symptomatic treatment and did not appear again with preconditioning. All infusion reactions occurred during combination therapy and did not recur after monotherapy. Possible explanation is the immunostimulatory activity of immune checkpoint inhibitors can lead to worsening of allergic reactions or inflammatory adverse events (e.g., dermatitis, infusion-related symptoms) associated with carboplatin and etoposide. The treatment can be continued after symptomatic treatment and adjustment of the dose of chemotherapy drugs. The combination treatment was generally safe and tolerable.

In accordance with the National Regulations on Drug Registration and Good Clinical Practice (GCP), as well as the chemical composition, pharmacokinetics, pharmacodynamics and toxicology research data of ZKAB001, the sponsor plans to conduct this Phase III trial to evaluate the efficacy and safety of ZKAB001 combined with EC versus placebo combined with EC for the treatment of extensive stage SCLC without prior systemic therapy.

# 2. OBJECTIVES AND ENDPOINTS

This trail is designed to evaluate the efficacy and safety of ZKAB001 combined with carboplatin and etoposide compared with placebo combined with carboplatin and etoposide for the treatment of ES-SCLC.

## 2.1 Primary Objective

To evaluate the overall survival (OS) of ZKAB001 combined with carboplatin and etoposide compared with placebo combined with carboplatin and etoposide as first-line treatment of ES-SCLC.

## 2.2 Secondary Objectives

- To evaluate the progression-free survival (PFS), objective response rate (ORR), disease control rate (DCR), duration of response (DOR), and OS rate at 1 year and 2 years for ZKAB001 combined with carboplatin and etoposide versus placebo combined with carboplatin and etoposide as first-line treatment of ES-SCLC;
- To evaluate the safety of treatment;
- To evaluate the relationship between PD-L1 expression status and clinical effects
- To evaluate the immunogenicity of ZKAB001
- To evaluate quality of life before and after treatment.

## 2.3 Endpoints

### 2.3.1 Primary Endpoints

Overall survival (OS)

### 2.3.2 Secondary Endpoints

- PFS assessed by investigators and IRC (based on RECIST v1.1)
- ORR assessed by investigators and IRC (based on RECIST v1.1)
- DoR assessed by investigators
- DCR assessed by investigators
- OS rate at 1 year and 2 years
- Incidence and severity of adverse events and severe adverse events, and abnormal laboratory measures (based on CTCAE 5.0)
- PD-L1 expression status in tumor tissue
- The positive rate of drug-resistant antibodies (ADA)
- Quality of life score (EORTC QLQ-C30 and QLQ-LC13)

# 3. STUDY DESIGN

## 3.1 Overall Design

This study is a randomized, double-blind, placebo-controlled, multicenter Phase III study. Eligible patients will be randomly assigned to the experimental group: ZKAB001+ carboplatin + etoposide, or the control group: placebo + carboplatin + etoposide at a ratio of 1:1, with every 3 weeks as one treatment cycle. Stratification factors included gender (male/female), PS score (0/1), and brain metastases (yes/no).

The study is consisted of a screening period, a treatment period (subjects will receive study treatment until confirmed disease progression, or intolerable toxic effect, or the maximum duration of 2 years, or subjects voluntarily requested termination of study treatment), and a follow-up period (including both safety and survival follow-up).


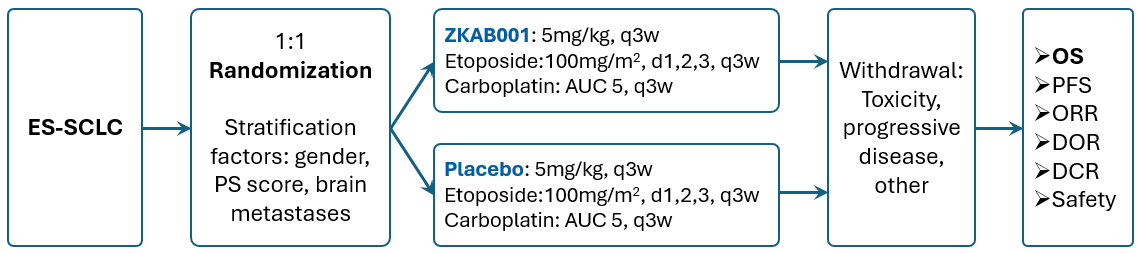


**Figure1. Study Design**

**Screening period**: Participants are required to undergo screening tests or assessments (some of which may be based on local results prior to signing the informed consent form, see study schedule) within 28 days prior to first dose to determine whether they meet the inclusion criteria and do not meet the exclusion criteria. Tumor samples (wax blocks of archived or freshly obtained tumor tissue or pathological biopsy sections within 6 months before first dose) will be collected from subjects during screening until 4 weeks after enrollment.

**Treatment period**: Study drugs will be given in a specified order at the first day of each cycle (every 3 weeks (-3 ~ +7 days) as 1 cycle). During the first 4 cycles, subjects will receive experimental drug/placebo combined chemotherapy, followed with maintenance therapy of experimental drug/placebo only from the 5^th^ cycle. Within 7 days prior to each dose, subjects must complete imaging examination, vital signs, physical examination, laboratory examination, physical status score, etc. Imaging examinations will be performed every 6 weeks ±7 days until confirmed disease progression or death, loss of follow-up, subjects' unwillingness to continue in the trial, or initiation of new tumor therapy.

**Safety follow-up period**: Within 90 days after the last dose of medication, safety follow-up should be conducted every 30 days ±7 days, and patients should return to the hospital for first evaluation as far as possible or conduct examination in a local hospital. If it is confirmed that more than one cycle (3 weeks) has passed since the last dose of medication at the time of withdrawal from the study, the end-of-trial examination should be the first safety follow-up after the end of medication. Information on survival status, adverse events, concomitant medication, and concomitant therapy can be collected at two subsequent telephone follow-up visits, until death, loss of follow-up, initiation of new anti-tumor therapy, or completion of all three safety follow-up visits.

**Survival follow-up**: All subjects will be followed up by telephone every 30 days after termination of treatment and safety follow-up, and survival status will be collected until death, loss of follow-up, or trial termination.

## 3.2 Sample Size Determination

In this study, OS was the primary endpoint, and the superiority test was performed with the control group. The parameters for calculating the sample size are as follows:

Efficacy hypothesis:

• Median OS (month) = 10.5 months (control group), HR=0.73;

• Random ratio: 1:1;

• Enrollment and follow-up time: enrollment period of 18 months + follow-up period of 18 months;

• Annual dropout rate: 5%;

We plan to do an interim analysis at 3/5 event count (60% data maturity), using O 'Brien Fleming Type 1 error consumption function. With 85% confidence in the OS test, a total of 369 OS events and approximately 498 subjects would be required to meet the target event.

# 4. RESEARCH POPULATION

## 4.1 Indication

Extensive-stage small cell lung cancer (ES-SCLC) without prior systemic treatment.

## 4.2 Inclusion Criteria

1. Male or female, age ≥ 18 years.
2. Small Cell Lung Cancer confirmed by histology.
3. Extensive-stage SCLC (defined as AJCC 8th Edition IV (Tany, Nany, M1a/b/c), or T3-4 SCLC that cannot be included in a tolerable radiotherapy plan due to multiple pulmonary nodules or tumor/nodule size).
4. Have not received first-line systemic treatment for ES-SCLC in the past.
5. Have received surgery and adjuvant therapy for cure, such as radiotherapy and chemotherapy, and there was an interval of at least 6 months from the last chemotherapy or radiotherapy to the diagnosis of ES-SCLC.
6. ECOG performance status of 0-1.
7. Have a life expectancy ≥ 8 weeks.
8. Have at least one measurable lesion (according to CT or MRI scan, based on RECIST v1.1) within 28 days before administration of the first dose.
9. Partners of male subjects and female subjects with childbearing potential must be willing to conduct effective contraception from signing the informed consent till 6 months after the last dose of study drug. And the human chorionic gonadotropin in blood / urine (HCG) test of female subjects with childbearing potential must be negative 7 days before the first dose.
10. Adequate hematologic, liver, kidney and coagulation function indicated by the following laboratory values:

- Blood routine test (without blood transfusion and hematopoietic factor drugs within 14 days before screening): White blood cell count ((WBC) ≥ 3.0x10^9); absolute neutrophil count ((ANC)) ≥ 1.5x10^9; platelet (PLT) ≥ 100x10^9; hemoglobin content ((hGB) ≥ 90g).
- Liver function: aspartate aminotransferase (AST) ≤ 2.5 ULN, alanine liver aminotransferase (ALT) ≤ 2.5 ULN; ALT and AST < 5 ULN; serum total bilirubin (TBIL) ≤ 1.5 ULN; albumin (ALB) ≥ 30 g L;
- Renal function: serum creatinine ≤ 1.5 ULN or creatinine clearance rate (Ccr) ≥ 40 mL/min (Cockcroft/Gault formula).
- Coagulation function: international standardized ratio (INR) ≤ 1.5 ULN, activated partial thromboplastin time (APTT) ≤ 1.5 ULN;
- Alkaline phosphatase (ALP) ≤ 2.5 ULN, bone metastasis subjects, ALP ≤ 5 ULN.

1. Able to provide tumor tissue samples that can meet the requirements of PD-L1 expression detection from screening to 4 weeks after enrollment.
2. Voluntarily participation through written informed consent，with good compliance and cooperation with follow-up.

## 4.3 Exclusion Criteria

1. Have received any T cell costimulatory or immune checkpoint inhibitors, including, but not limited to, cytotoxic T lymphocyte associated antigen-4 (CTLA-4) inhibitors, PD-1 inhibitors, PD-L1/2 inhibitors or other drugs targeting T cells; anti-vascular endothelial growth factor (VEGF) or vascular endothelial growth factor receptor (VEGFR) therapy.
2. Active brain metastasis or meningeal metastasis. Patients with brain metastasis after treatment need to meet the following criteria to be enrolled: asymptomatic; no imaging evidence of progress for at least 4 weeks after treatment; completion of treatment for at least 7 days before the first dose of the study drug; do not need to receive systemic corticosteroids (> 10mg/ prednisone or equivalent) for 14 days before the first dose of the study drug. If a new asymptomatic brain metastasis is found during the screening period, radiotherapy and/or surgery will be required. If all other criteria are met after treatment, additional brain scans are not required before randomization.
3. Radiotherapy: the completion of radiotherapy for the brain or palliative radiotherapy for the focus of bone lesion is within 7 days before the first dose of the study drug.
4. Active, known or suspected autoimmune diseases, including, but not limited to, myasthenia gravis, myositis, autoimmune hepatitis, systemic lupus erythematosus, rheumatoid arthritis, inflammatory bowel disease, vascular thrombosis associated with antiphospholipid syndrome, Wegener's granuloma, Sjogren's syndrome, Guillain-Barre syndrome, multiple sclerosis, vasculitis or glomerulonephritis. Cases that can be included: residual hypothyroidism due to autoimmune thyroiditis that require hormone replacement therapy, well-controlled type I diabetes, or no recurrence expected in the absence of external stimulation; eczema, psoriasis, neurodermatitis or vitiligo (psoriatic arthritis patients will be excluded) with only topical presentation can be included if they meet the following criteria: the area covered by the rash must be less than 10% of the body surface area; the disease is well controlled at the baseline level, requiring only inefficient topical steroids, and with no acute exacerbation in the past 12 months.
5. Uncontrolled pleural effusion, pericardial effusion or ascites requiring repeated drainage (once a month or more frequently). Patients who use indwelling catheters are allowed to be included.
6. Corticosteroids (> 10 mg/ prednisone or equivalent dose) or other immunosuppressants were used within 14 days before the first dose. Inhalation or topical use of steroids and adrenal replacement steroids are allowed in the absence of active autoimmune disease; for patients receiving short-term, systemic immunosuppressive therapy, for example, glucocorticoids for nausea, vomiting, or allergic reaction management or preventive use can be enrolled after consultation with the sponsor. The use of mineralocorticoid in the treatment of postural hypotension and the use of low-dose glucocorticoid supplements in the treatment of adrenocortical insufficiency is allowed.
7. Patients who had been vaccinated within 4 weeks before first dose or planned to receive live vaccines.
8. Major surgery was performed within 4 weeks before first dose of study drug, or major surgery was scheduled during the study period.
9. Interstitial pneumonia (ILD) disease, drug-induced pneumonia, radiation pneumonia requiring steroid treatment or active pneumonia with clinical symptoms.
10. Active pulmonary tuberculosis or history of active pulmonary tuberculosis infection within 1 year before screening, whether treated or not.
11. Uncontrolled cardiovascular diseases, such as: (1) heart failure (New York Heart Association (NYHA) grade 2 or above) (2) unstable angina pectoris (3) myocardial infarction or cerebrovascular accident within 6 months (4) clinically significant supraventricular or ventricular arrhythmias that needs to be treated.
12. Uncontrolled active infections (e.g. those who need intravenous antibiotics, antifungal or antiviral therapy).
13. Active hepatitis B or C (HBV-DNA titer < 500IU/mL or copy number < 1000copies/ml, HCV-RNA negative after antiviral treatment can be included), HIV positive or known history of acquired immunodeficiency syndrome.
14. Known allergies to study drugs or excipients; known severe allergic reactions to any monoclonal antibody; allergic history of carboplatin or etoposide.
15. Patients who have previously received allogeneic bone marrow transplantation or solid organ transplantation.
16. Other malignancy diagnosed less than 5 years before the first dose, except for fully treated cervical carcinoma in situ, basal cell or squamous cell skin cancer, local prostate cancer after radical resection, and ductal carcinoma in situ after radical mastectomy.
17. Have been treated with any other experimental drugs or participated in another interventional clinical study within 4 weeks before signing ICF.
18. Pregnant or lactating women.
19. Known cases of mental illness, alcohol abuse, inability to quit smoking, drug use or substance abuse.
20. Other situations assessed by the investigators to be unsuitable for participation in this study.

## 4.4 Criteria of Termination of Treatment

In some cases, termination of treatment does not mean withdrawal from the study. Patients can discontinue treatment at any time and for any reason. In addition, the investigator or sponsor may terminate a patient's treatment if the patient is not suitable for treatment, violates the study protocol, or for administrative and/or other safety reasons.

Patients must discontinue treatment for any of the following reasons, but may continue to be monitored:

1) The patient or his legal representative requests the termination of treatment;

2) The maximum duration of use (2 years) was reached;

3) Confirmed disease progression or clinical deterioration;

4) Intolerable adverse reactions, which meet the standards of permanent withdrawal;

5) pregnancy;

6) Laboratory Abnormalities or other medical conditions that make it no longer beneficial to continue the treatment;

7) Other circumstances assessed by the investigator and/or sponsor to be necessary to discontinue the treatment.

## 4.5 Criteria of Withdrawal from the Study

Patients have the right to withdraw from the study at any time for any reason and should withdraw from the study if any of the following conditions are met.

1) The subjects are unwilling to continue to participate in the study and refuse further follow-up;

2) Any clinical adverse events, laboratory abnormalities, or co-morbidities making the investigator believe that continuing participation in the study is not in the subject's best interest;

3) Receiving new anti-tumor therapy;

4) Other circumstances in which the investigator considers it necessary to withdraw from the study;

5) Loss of follow-up

6) Death

7) The sponsor terminated the study

In any case, after termination of treatment or withdrawal from the study, the subject's final state should be recorded as far as possible. Efforts should be made to complete efficacy and safety assessments at the time of exit as specified in the protocol, as well as to complete the safety follow-up period, and to fully document adverse events (AEs) and outcomes. According to the actual situation of the subject, the investigator may suggest or offer new or alternative treatments to the subject. Subjects without disease progression should be followed for imaging evaluation until subjects begin new antitumor therapy or disease progression.

# 5. SUBJECT RECRUITMENT

Vulnerable subjects are not allowed to be enrolled, including but not limited to those who are unable to complete informed consent. Subjects may be inpatient, outpatient, or from the public. Recruitment advertisements approved by the Ethics Committee can be placed on the official website of the clinical research institution of each research center or the publicity column of the department. All reasonable efforts should be made to locate the subject to determine and report his or her current status, including those authorized by the subject to provide information about him or her (family members or their representatives).

Prospective subjects will attend a screening visit to determine eligibility for enrollment. In addition to the results of previous examinations recognized by some protocols, adequate informed consent with the subject is required before any protocol-related procedures are initiated (i.e., prior to screening procedures), and the subject's signed written informed consent is obtained before implementation. At the screening visit, each subject will be assigned a unique screening number by the study center for identification. Record the results of each screening in the subject screening form.

# 6. DOSAGE AND DOSING SCHEDULE

## 6.1 Packaging and Labeling

The investigational drugs of ZKAB001 or placebo and study drugs of carboplatin injection or etoposide injection, are provided by sponsor. The original packaging of the drug remains unchanged and ‘For Clinical Trial Use Only’ must be labelled at each outer packaging box of drug.

The label information is listed as follows: drug name, strength, manufacture date or expiry date, lot number, name and address of sponsor, and storage condition.

## 6.2 Study Drugs

1. Investigational drug: Socazalimab

Drug Code：ZKAB001

Strength: 100 mg/4 mL/vial

Storage：stored in temperature 2 - 8℃

Shelf life: 36 months (temporarily)

Drug supplier: Zhaoke (Guangzhou) Oncology Pharmaceutical Ltd.

2. Placebo

Same information as above.

3. Carboplatin injection

Information seen drug labels.

4. Etoposide injection

Information seen drug labels.

## 6.3 Drug Accountability

### 6.3.1 Drug Supplying and Receiving

Prior to study initiation, sponsor will deliver one batch of study drugs (placebo included) to study center in accordance with the number of patients enrolled in each center, and the drug number is produced by randomization and trial supply management (RTSM). Drugs for clinical trial use will be received by designated personnels in each study center, thereby establishing complete recording procedures for receiving drugs and preservation under study drug storage conditions.

During the trial, when the drugs kept by the center reach the RTSM-prescribed alert line, the sponsor will dispatch a new batch of drugs of which number will be generated by RTSM, depending on the actual enrollment progress of each center.

### 6.3.2 Drug Management

The designated personnels are responsible for management on dispensing drugs to each study center as follows:

- Dispense and return of the study drugs must be recorded;
- All study drugs must be safely and properly stored and disposed；
- All the investigational drugs must be distributed according to the clinical trial protocol;
- Study drugs should be dispensed to the subjects in accordance with the randomization information；
- All the unused study drugs (including drugs returned by patients) with their outer packages will be returned to the sponsor；
- The application of study drugs in whole clinical trial must be recorded in the corresponding drug inventory form；
- Maintain a detailed inventory of investigational drugs, including details of drugs received, times and exact record of drug dispense；
- The record of drug dispensing must be consistently corresponding to used and unused drugs, in which any deviation must be further explained and any relevant form regarding dispensing and return must be signed by personnels who are authorized for study drug management.

### 6.3.3 Drug Storage

The study drugs will be stored according to requirements on the label with regular temperature record.

- The study drugs are not allowed to provide anyone except for patients enrolled in this study.
- The study drugs should be stored at pharmacy or other locked and secure storage facilities that contain the controllable storing conditions, where are merely open to personnels who are authorized for study drug management.

### 6.3.4 Preparation and Disposal of Study Drugs

If the vial is broken, the study drug is discolored or other damages are found, the study drug must not be used, and the sponsor must be contacted immediately.

Dilute 25mg/ml of ZKAB001 injection with 0.9% sodium chloride to 250ml final volume. It cannot be administered as an intravenous bolus. Intravenous infusion time must be at least 60 minutes. The infusion time can be extended due to infusion reactions. When the infusion is completed, flush the infusion catheter with saline.

The specific steps are as follows:

1. Before preparation, take out the required number of ZKAB001 injection vials and let them stand at room temperature for 5 minutes to confirm that the injection is clear, colorless, and visually free of particulate matter.
2. Under aseptic operation, use a syringe to extract the required amount of ZKAB001 injection, and inject it into the 250ml normal saline infusion bag (Note: Do not draw the ZKAB001 injection multiple times from the vial. Do not use the glass syringe to extract the ZKAB001 injection) .
3. Invert the infusion bag several times and mix gently. Do not shake.
4. Visually inspect the prepared solution. If the prepared solution is turbid, or there is a suspected deposit, please change the study drug and record it in the study drug count table.
5. Indicate the preparation time and dosage of ZKAB001 on the infusion bag.
6. Connect the prepared ZKAB001 solution to the infusion catheter.
7. intravenous infusion time ≥ 60 minutes.
8. When the infusion is completed, flush the infusion catheter with adequate saline.

It is recommended to use it within 4 hours (including intravenous infusion time) under normal temperature and static state after preparation, so as to avoid excessive exposure at room temperature. If the drug needs to be delayed due to special circumstances, the diluted drug can be stored at 2-8℃ for up to 12 hours.

### 6.3.5 Drug Count

The research center receives the research drugs and stores them in a safe place, and only the researcher and designated personnel can get these drugs. After receiving the medicine, it needs to be stored according to the instructions of ZKAB001.

The investigator needs to accurately record the transportation and distribution quantity of the study drug in the drug record. The CRA in charge records the drug quantity during each center visit and when the study is completed.

At the end of the study or during the study period, the investigator shall return all used and unused study drugs, packaging, drug labels and copies of drug quantity records to Zhaoke (Guangzhou) Oncology Pharmaceutical Ltd.

### 6.3.6 Return and Destruction of Study Drug

All unused and/or partially used study drugs must be collected and destroyed by the sponsor. Each time when the study drug is destroyed, a detailed drug destruction record form must be completed and signed by the site certifier to confirm the destruction.

## 6.4 Study Drug Administration

### 6.4.1 Pretreatment

- During combination therapy: oral nonsteroidal antipyretic and analgesic drugs (celecoxib, meloxicam, etc.) and antihistamines (cetirizine, loratadine, etc.) are recommended prior to combination therapy. After ZKAB001 infusion is completed and before administration of chemotherapy drugs, small doses of intravenous or oral corticoids (e.g., intravenous 5 mg dexamethasone) are administered.
- During maintenance therapy of ZKAB001/placebo: continuous administration of oral nonsteroidal antipyretic and analgesic drugs and antihistamines prior to use of study drugs depending on treatment practice or investigator’s judgment of each center.

### 6.4.2 Administration of ZKAB001 and Placebo

The dose was 5 mg/kg/dose for 21 days (3 weeks) as a treatment cycle. Until an event occurs that meets the criteria for withdrawal from the trial. Details can be seen in study diagram (Appendix 5).

Patients who fulfill the following conditions can proceed to the next cycle of treatment：

1. Subjects with a best composite outcome of CR, PR, or SD may continue to be treated with study drugs until the first occurrence of: 1) achievement of confirmed PD; 2) clinical deterioration suggesting that further treatment is likely not to result in additional clinical benefit; 3) meeting the criteria for discontinuing the study; 4) intolerance of the study drugs.
2. Despite of confirmed disease progression, there is no clinical deterioration and a stable or improved clinical status is demonstrated, therefore, treatment with the study drug should be continued until further progression or clinical deterioration occurs. Criteria for continuous treatment are as follows：

1. Continuous treatment is suitable for the best interest of patients from investigators’ judgement;
2. Patients could stay with stable disease status without immediate initiation of other antitumor therapy；
3. Patients can tolerate continuous treatment；
4. Patients have no significant decrease in physical status and no significant worsening of tumor-related symptoms；
5. Continuous treatment will not interfere with interventions that are urgently needed to prevent serious complications of disease progression (e.g. brain metastases).

The assessment of clinical benefit shall be considered whether the subjects appear clinical deterioration and benefit from continuous treatment. If subjects continue to be treated, examination and evaluation according to the study process are needed. Since it is not possible to absolutely differentiate pseudo-progression and true tumor progression, drug administration still continues and further progression may subsequently occur despite of ineffective treatment. It is recommended that investigators conduct tumor assessments at least every 4 weeks after the first disease progression confirmed by RECIST v1.1, until further disease progression on the next tumor assessment and the achievement of other criteria for discontinuation of the drug, all of which the patients should withdraw from the study.

### 6.4.3 EC Administration Schedule

A. Carboplatin injection; Strength: 10ml: 100mg; Dosage: administration on day 1, AUC=5, 21 days (3 weeks) as a treatment cycle. Mode of administration: intravenous drip.

Calculation of carboplatin: Calvert formula: dose of carboplatin (mg)=5 AUC (mg/ml/min) × [creatinine clearance rate (ml/min) +25].

Preparation and drip methods should be referred to carboplatin injection instructions and local medical practices.

B. Etoposide injection; Strength: 0.1g/5ml; Dosage: continuous administration on day1, day 2, and day 3, 100mg/m^2^, 21 days (3 weeks) as a treatment cycle. Mode of administration: intravenous drip.

Preparation and drip methods should be referred to etoposide injection instructions and local medical practices.

Patients with a best composite effect of PR or SD may continue treatment until 4 cycles have been achieved; the remaining criteria of termination are the same in section 6.4.1.

### 6.4.4 Sequence of Administration of Study Drugs

The medications were given sequentially on day 1 of each cycle with ZKAB001 given firstly with the intravenous infusion time ≥60 minutes. When the infusion is completed, flush the infusion catheter with adequate saline. Carboplatin is given secondly with the intravenous infusion time within 30-60 minutes. Etoposide is given last with the intravenous infusion time ＞60 minutes or adjusted according to medical practice standards of each sites.

The time window for subsequent dosing is 3 weeks (-3 to +7 days), and the drug should be administered as close as possible to the time specified. Delays in dosing due to drug-related adverse events are not restricted to the time window. Within 7 days prior to each dose, patients must complete required clinical examinations to assess the tolerability of continued dosing.

## 6.5 Dose Adjustment and Discontinuation

### 6.5.1 Dose Adjustment and Discontinuation of ZKAB001

1. No dose adjustment.
2. The time window for each dose is calculated from the date of the first administration. If the delay is more than 7 days but no more than 14 days, it is recommended that the time window be recalculated for the next cycle based on the actual date of current cycle. If there is a delay more than 14 days, it is recommended that the dose not be replenished in the current cycle and the dose of next cycle is still based on the time window calculated from the date of the first administration. Toxicities which leads to delayed infusion/discontinuation are as follows:
   - Immune-mediated pneumonia: grade 1, discontinuation is not necessary, but suspension of medication may be considered in the condition of clinical indications and diagnostic examinations for other etiologies; grade 2: suspend the medication, re-administer the medication when return to grade 0-1. Permanent discontinuation is needed for recovery time ≥14 days，grade 3-4 immune-mediated pneumonia or grade 2 or higher recurrent immune-mediated pneumonia.
   - Immune-mediated endocrine disease: including but not limited to hypophysitis, hypothyroidism/thyroiditis or hyperthyroidism, and type 1 diabetes，continuous treatment is permitted when clinical stability can be reached with hormone replacement therapy.
   - Other immune-mediated adverse events: including but not limited to immune-mediated myositis, immune-mediated enteritis, immune-mediated hepatitis, immune-mediated nephritis and renal insufficiency, immune-mediated adverse skin reactions and immune-mediated encephalitis. No dose adjustment for grade 1. Permanent discontinuation is needed for grade 2-3 immune- mediated adverse reactions fail to return to grade 0-1 within 12 weeks after last dose (except endocrine diseases), and corticosteroids fail to decrease by ≤10 mg/day prednisone equivalent dose within 12 weeks of last dose.
   - Infusion-related adverse reaction: Signs and symptoms of an infusion reaction include, but not limited to, fever, chills, headache, rash, itching, joint pain, hypotension/hypertension, bronchospasm, and other symptoms. Grade 1: Reduce the drug infusion rate to 50% and monitor closely for further deterioration. If the infusion reaction still exists at this infusion rate, it is necessary to further reduce the infusion rate. Grade 2: Stop the study drug infusion. According to medical indications, administer adrenaline, glucocorticoids, intravenous antihistamines, bronchodilators, and / or oxygen. If the infusion reaction is relieved or the severity is reduced to grade 1, the infusion can be restarted at 50% of the original infusion rate, and at the same time, whether any deterioration has occurred is closely monitored. If the infusion reaction occurs again at this infusion rate, the infusion rate can be further reduced. The longest infusion time is 3 hours. Grade 3 or 4: Discontinue treatment of study drug permanently and take treatment measures above. Prophylactic treatment (antihistamines, NSAIDs, etc.) should be given prior to subsequent infusions to patients with any grade of infusion reaction. The decision to terminate treatment should be discussed with the sponsor for patients with recurrence of any infusion reactions after prophylactic treatment.
   - Study drug must be permanently terminated in the following situations: Abnormal grade 3 drug-related laboratory tests do not require termination of treatment. However, if grade 3 thrombocytopenia occurs> 7 days or related to bleeding, the study medication must be terminated. Any grade 4 drug-related AE or laboratory abnormality, except for the following: grade 4 neutropenia < 7 days; grade 4 lymphopenia or leukopenia; grade 4 alkaline phosphatase (AKP) elevation; Isolated grade 4 electrolyte imbalance / abnormality that is not accompanied by clinical sequelae and can be corrected by supplementation/appropriate treatment within 72 hours after its occurrence.
   - Unspecified criteria of adverse events for delayed infusion/permanent discontinuation: As for the occurrence of clinical adverse events, laboratory abnormality, or concomitant disease, discontinuation of study drugs should be judged by investigators if continuous administration of study drugs leads to significant risks to the patients.

Management of immune-mediated adverse events due to ZKAB001 should be in compliance with clinical practice of each site or guidelines of Chinese Society of Clinical Oncology (CSCO): Management of Immune Checkpoint inhibitor-related toxicity, 2019.

### 6.5.2 Dose Adjustment and Discontinuation of EC combinations

**Dose adjustment of carboplatin:**

Dose adjustment of carboplatin is permitted according to prescription information and adverse reactions. Dosage adjustment recommendations are provided below. The dose should not be increased back to the original dose after reduction except for specified regulations. The carboplatin treatment should be terminated if there are any grade 3 or higher hematological/non-hematological toxicities (except for fever with exact causes such as infection or neoplasia, grade 3/4 AKP elevation) with 2 times of dose reduction due to toxicities or 6 weeks after delayed treatment.

1）Hematological toxicities

At the beginning of each cycle, ANC ≥1.5×10^9^/L and PLT ≥100×10^9^/L are required. To provide adequate recovery time, treatment can be delayed for up to 6 weeks. After recovery of hematological toxicities, at the beginning of subsequent cycles, the dose of subsequent cycles will be adjusted based on the lowest values of PLT and ANC in the previous cycle (see Table 1). Patients who need to adjust their dose due to both ANC and PLT will be treated with a lower dose. If the dose calculated according to AUC in subsequent cycles is still lower than the dose after reduction, the dose calculated this time should be used.

**Table 1 Dose adjustment for hematologic toxicity**

| Toxicity^a^ | Dose of carboplatin (% of previous dose) |
| --- | --- |
| ANC<0.5×10^9^/L and PLT≥25×10^9^/L | 80% |
| PLT< 25×10^9^/L, without considering ANC | 80% |
| PLT<50×10^9^/L with ≥ grade 2 bleeding or need for blood transfusion, without considering ANC | 50% |
| ANC<1.0×10^9^/L and fever (T ≥ 38.5℃) | 80% |

a: The nadir of the previous treatment cycle.

Reduce dose permanently when neutropenia with fever or thrombocytopenia (PLT <25×10^9^/L or <50×10^9^/L with bleeding or need for blood transfusion) occurs for the first time. If neutropenia with fever or thrombocytopenia occurs for the second time, the investigators decide on the range of dose reduction。

If carboplatin is discontinued due to hematologic toxicity, complete blood count should be monitored weekly until the end of the study or return to the lower limit. Subsequent normal treatment process is followed.

Anemia can be treated with appropriate support therapies without dose reduction.

2）Non-hematological toxicities

If non-hematologic toxicities occur (see Table 2), treatment should be delayed (up to 6 weeks) until test values return to ≤ grade 1 or the baseline status. At the beginning of subsequent cycles, the dose is reduced based on the non-hematologic toxicity of the dose administered in the previous cycle (see Table 2).

**Table 2 Dose adjustment or treatment discontinuation for non-hematologic toxicities**

| Toxicities | | Modified carboplatin dosage (% of the previous dose)^a^ |
| --- | --- | --- |
| diarrhea | Grade 3/4^b^ | 80% |
| Nausea/vomiting | Grade 3/4^c^ | 80% |
| Neurotoxicity | Grade 2 | 80% |
|  | Grade 3/4 | 50% or permanent discontinuation |
| Hepatic transaminase elevation | Grade 3 | 80% |
|  | Grade 4 | discontinuation |
| others | Grade 3/4 | 80% |

a. If deemed appropriate by the investigators, the dose of carboplatin will be adjusted by the specific percentage of the original AUC.

b. The use of antidiarrheal medications is recommended for diarrhea of grade 3/4 or any grade cases requiring hospitalization.

c. Including antiemetics used.

Nausea and/or vomiting can be controlled with appropriate antiemetics. If grade 3/4 nausea/vomiting occurs, the dose can be reduced by 20% in the next cycle. The dose should be increased to 100% as soon as possible if the antiemetics can be tolerated.

**Dose adjustment of etoposide:**

The dose of etoposide should be adjusted in the same proportion as carboplatin. Once reduced, it cannot be increased back to 100% of the original dose. Treatment with etoposide should be discontinued if the subject has any of grade 3 or higher hematologic or non-hematologic toxicities (except for fever with definite causes such as infections or tumor, grade 3/4 AKP elevation) after two dose reduction or treatment delays more than 6 weeks due to toxicities. In addition, recommendations on dose adjustments of etoposide due to renal impairment are shown in Table 3.

**Table 3 Dose adjustments of etoposide due to renal impairment**

| Creatinine clearance rate(mL/min) | Dose of etoposide |
| --- | --- |
| > 50 | 100% |
| 15 – 40 | 80% of previous dose |

The actual dosage of each drug must be recorded, as well as the start time and end time.

Once discontinuation occurs due to adverse events, all the study drugs should be resumed synchronously until level of medication available is reached.

After discontinuation of carboplatin or etoposide due to toxicities, ZKAB001/placebo could be continued if the participants recover to meet the administration requirements for ZKAB001/placebo.

Superimposed toxicities: Theoretically, the immunostimulatory activity of PD-1/L1 inhibitors could lead to exacerbation of chemotherapy-related allergic reactions or inflammatory adverse events (e.g., dermatitis, infusion-related symptoms). Treatment discontinuation and dose adjustments is suitable for actual conditions (if applicable). Oral nonsteroidal antipyretic and analgesic drugs and antihistamines prior to each combination medications and small doses of glucocorticoids after ZKAB001 infusion but prior to chemotherapeutic drug administration are recommended.

As for such adverse events that continue to occur on re-administration after treatment and pretreatment, the investigator need to assess the superimposed toxicities of combination medications and discuss with the sponsor whether to continue the combination treatment, or to continue with chemotherapy and administration of ZKAB001/placebo maintenance after completing chemotherapy, or to continue with maintenance with ZKAB001/placebo only. Other additional tests, such as autoimmune-related laboratory tests or other examinations, may be performed to determine the possible immunologic etiology of the adverse events above. It is recommended for discontinuation of ZKAB001/placebo and relevant treatment if necessary when ZKAB001/placebo is considered to be a potentially relevant factor.

The investigator shall determine the relationship between adverse events and study drugs and chemotherapeutic drugs and decide whether to discontinue the administration of the drug based on the subjects’ conditions if any adverse events occur during the administration of the drug. The dosage and the basis for the decision to discontinue and dispositions shall be documented in the medical record and CRF

# 7. CONCOMITANT TREATMENTS

All drugs used by the patients in the 28 days before the start of the study and during the study (except for solvents, such as saline, glucose injection, etc.) will be recorded in the relevant part of the eCRF table, and the reason for use and details of use must be indicated.

## 7.1 Contraindicated Drugs and Treatments During the Study

- NMPA-approved Chinese medicine formulations possessing antitumor efficacy and immunomodulators (e.g., interleukin-2, interferon, etc.) are prohibited during study period.
- Localized treatment for primary diseases and systemic antitumor therapy are prohibited (Palliative pain-relieving radiotherapy for bone metastasis is permitted).
- Concomitant immunosuppressive therapies are not permitted (except for dealing with drug-related adverse events).
- Participating in other clinical trials is not allowed.

## 7.2 Permitted Drugs and Treatments During the Study

- Antiemetics

Prophylactic antiemetics should be applied appropriately in treatment of EC therapy and prophylactic antiemetics with chemotherapy of ZKAB001 alone is at the discretion of the investigators’ judgment of symptoms. Prophylactic antiemetics regimens applied in EC therapy can be seen in Table 4. Prophylactic use of dexamethasone is not recommended, and the dose should be reduced to the lowest clinically acceptable dose for therapeutic purposes.

**Table 4 Single-day intravenous vomiting prophylaxis program for high emetic risk**

| **Classification of drugs** | **Day 1** | **Day 2** | **Day 3** | **Day 4** |
| --- | --- | --- | --- | --- |
| 5-HT_3_RA（choose one of them） | - Palonosetron: 0.25mg IV/0.5mg PO, once - granisetron: 3mg IV/2mg PO, once or 3.1mg/24h (1 dose of transdermal patch, applied 24-48h in advance) - tropisetron：5mg IV/PO, once - ondansetron：8-16mgIV/16-24mg PO, once - azasetron：10mg IV/PO, once - ramosetron: 0.3mg IV, once |  |  |  |
| NK-1RA (choose one of them) | - aprepitant：125mg PO, once - fosaprepitant：150mgIV, once - NEPA capsules：one pill PO, once | aprepitant 80mg PO, once (If Day 1 is aprepitant) | aprepitant 80mg PO, once (If Day 1 is aprepitant) |  |
| dexamethasone | 6-12mg IV/PO, once | 3.75~8mg IV/PO, once | 3.75~8mg IV/PO, once | 3.75~8mg IV/PO, once |

Antiemetics need to be used before treatment of antineoplastic drugs each time. Highly emetogenic regimens (carboplatin AUC ≥4 is classified as highly emetogenic) continue for at least 3 days after the end of antineoplastic treatment. The choice of antiemetic regimens is based on the highest emitting risk drugs for multidrug combination regimens. Be careful to screen other potential causes of vomiting, such as brain metastasis (from Guidelines of Chinese Society of Clinical Oncology （CSCO）: Prevention & treatment of nausea and vomiting caused by antitumor therapies 2019).

- Treatment to prevent allergic reactions

It is recommended to take oral non-steroidal antipyretic and analgesic drugs and antihistamines before treatment of study drugs each time during the period of combination therapy. Before single-agent maintenance therapy, two drugs above are allowed to be taken orally in advance according to diagnosis and treatment routine of each research center and the judgment of investigators.

- Antiviral therapy

Patients with HBV and HCV infections should receive antiviral therapy according to local standards, and recommendations are as follows:

Patients with HBV infection, such as positive for HBV-DNA, need to receive antiviral therapy with HBV-DNA < 500 IU/ml before enrollment.

Patients with HCV infection who are HCV-RNA positive should receive antiviral therapy.

- Steroids

Topical use of steroids, such as topical external use, inhalation, etc., is permitted. The use of corticosteroids (e.g., thyroxine) for replacement therapy is permitted. Low-dose of intravenous or oral Corticoids for the treatment of adverse effects are permitted after the completion of the ZKAB001 infusion and before the use of chemotherapy drugs during combination chemotherapy period. Temporary use of corticosteroids (cumulative time not more than 1 week) will be allowed if clinically indicated and deemed necessary by the investigators to manage patients’ non-immunotherapy-related events (e.g., chronic obstructive pulmonary disease, prevention of contrast allergy, etc.). The condition that the patients need to prolong the duration of corticosteroids due to illness should be discussed with the sponsor.

- Vaccines

The inactivated vaccines to prevent infectious diseases can be used.

- Other systemic treatments

During treatment, optimal supportive care is allowed

- Surgery (non-anti-tumor purposes)

Any surgery performed during the study period should be with rationales and necessity. The decision to re-administer the drug postoperatively depends on the clinical assessment of wound healing and postoperative recovery.

- Palliative care or therapy

For example, local radiation therapy for pain relief and thoracentesis to reduce discomfort can be performed at the discretion of the investigator. Palliative local therapies (including palliative radiotherapy and palliative surgical excision of symptomatic non-target bone lesions, skin lesions, or brain lesions, drainage of symptomatic pleural effusions, pericardial effusions, and ascites effusions) are allowed. Palliative radiotherapies are allowed if the following conditions are met: treatment of focal lesions causing significant symptoms and these lesions are known to be present when enrolled and are not target lesions; palliative radiotherapies are initiated and discussed with the sponsor for confirmation. During maintenance therapy, prophylactic brain radiation may be administered according to protocols of sites, but curative thoracic radiation therapy to treat or eliminate residual disease is not allowed with the exception of palliative chest radiotherapy.

Patients are required to use the same concomitant medications. Addition of new concomitant medications during the study period, or adjustments to the dose or dosing regimens of the current combination therapies should be documented in the appropriate section of the eCRF.

# 8. STUDY PROCEDURES

## 8.1 Informed Consent

Prior to initiation of any procedure of the study, informed consent form (ICF) approved by Investigational Review Board (IRB) is required. The investigator must explain the properties, purposes, underlying risks and benefits, and requirements of study included in the informed consent form to each potential patient. The investigator needs to provide chances to each potential patient for raising questions regarding the study until he or she is satisfied with the answers, and meanwhile the investigator will also explain the properties and purposes of ICF to each potential patient. If the potential patient consents, he will be asked to sign and date a written ICF. No study procedure will be implemented until the ICF is signed except for previous examinations recognized in the study. The investigator or designated person who obtained informed consent from the patients can also sign on the ICF to confirm that this informed consent is obtained as required. Patients will sign the informed consent form and be screened within 28 days prior to enrollment, and a copy of the signed ICF will be handed to the patients.

In addition to the tumor imaging and other examinations established within the specified time limit before the first dose, the written informed consent must be obtained before the start of any clinical study procedure.

Subjects who have failed previous screening are allowed to be screened again, and at the time of re-screening, they must re-sign the informed consent form and re-register to obtain a new subject number, and a maximum of two screenings will be conducted for a subject.

## 8.2 Treatment Assignment and Blinding

### 8.2.1 Identification of Codes and Blinding

After the subject signs the informed consent form, the investigator will input the subject's basic information into the RTSM system, which will automatically assign an identification code (subject screening number) to the subject. The identification code consists of 5 digits, of which the first two represent the central serial number and the last three represent the subject's serial number. The investigator/clinical coordinator (CRC) will fill in the identification code assigned by the RTSM into the “Subject Identification Code List” and save it.

Participants will be randomly assigned to receive either ZKAB001 or placebo in a double-blind approach in the study. The investigator assigns a random number to the subject based on the information obtained from the RTSM. ZKAB001 and placebo will be in the same package in order to remain blind. Neither The subjects, the investigators, the person involved in the subjects’ treatment, and the sponsor or their designees are all blind to the grouping.

### 8.2.2 Randomization

In this study, a stratified block randomization method will be adopted, and the qualified subjects will be randomly assigned to the experimental group or the control group in a 1:1 ratio. Randomization of participants is stratified by sex (male or female), brain metastasis (presence or absence of brain metastasis), and PS status (0 or 1).

The drug should be administered as soon as possible within the time limit for meeting laboratory and other tests or assessments after randomization.

### 8.2.3 Compliance Control

Subjects are required to return to the hospital according to the requirements of the protocol to complete all treatment cycles and related operation requirement.

The number of treatment cycles of the subjects, the actual amount and duration of the study drugs received in each cycle, and the reasons for violating the treatment plan should be recorded in the original medical records and CRF to judge the compliance of the subjects.

The clinical research coordinator and the investigator should maintain close communication with the participants, try to explain the questions of the subjects about any medical procedure and medical event during the study, and inform the participants that poor compliance may lead to withdrawing from the study to improve the compliance.

### 8.2.4 Unblinding

This is a double-blind trial. It is necessary to avoid unblinding the treatment status of the subject as much as possible, and unblinding is generally only performed after consultation between the investigator and the sponsor. The situations involved in unblinding are as follows:

- Investigator-side unblinding (emergent unblinding)
- Unblinding for SUSAR reporting
- Unblinding after the end of the clinical study

Emergent unblinding should be the last option for the condition that it is necessary to know the distribution of treatment in order to make a treatment decision or other medical need. When the investigator or agent need to confirm the drug used by the subject, the principal investigator of the center shall submit an application, then the sponsor and the principal investigator of the team leader shall codetermine whether to unblind or not. The investigator or surrogate must record the toxicity grade of the observed adverse event, its relevance to the study drug, and the cause in documents such as medical records prior to unblinding.

Generally, a serious adverse event does not require immediate unblinding. If the tumor recurs or progresses, the subject's data will not be unblinded, and/or treatment will not be discontinued. Unblinding is not required in the following cases:

- Non-serious adverse events
- Serious adverse events related to the clinical trial procedure or concomitant medications and irrelevant to the study drug
- Serious adverse events are clinical study endpoint events
- Subjects withdraw from the study for personal reason

Once unblinded, the subject must discontinue the study drug and continue to complete follow-up evaluation specified for the protocol.

The investigator must document the reason for unblinding in CRF. It is strongly recommended that the central investigator aware of the blinding results does not disclose the subject's treatment grouping to any individual who is not directly involved in the management of a medical emergency.

## 8.3 Independent Radiology Evaluation

Radiology evaluation will be performed at each study center, and independent imaging evaluations will be performed using an Independent Radiology Review Committee (IRC) for efficacy endpoints, namely PFS, ORR, DCR and DoR.
 Imaging evaluations are performed by an experienced and qualified physician designated by each center. At the same time, each center should burn all imaging examination data related to efficacy evaluation into a CD-ROM for archiving, and regularly send or upload it to IRC for evaluation.

All experts from study centers and IRC evaluate the efficacy based on tumor imaging according to the RECIST v1.1.

## 8.4. Data Monitoring Committee

An Independent Data Monitoring Committee (IDMC) will be established to conduct regular evaluations of the safety and efficacy data of the study in accordance with the provisions of the IDMC regulations. The specific composition of personnel, responsibilities and related regulations are detailed in the IDMC regulations.

## 8.5 Observation Items

These assessments include demographic characteristics, medical history, physical examination, vital signs, 12-lead ECG, laboratory tests, imaging examinations, etc.

- Demographic characteristics

Gender, height, age, and weight are recorded during the screening period. Weight should be measured within 7 days prior to each treatment cycle and the body surface area should be calculated.

- Medical history

Medical history including clinically significant past and concomitant disease (or signs and symptoms) prior to signing the ICF will be recorded in the eCRF at the screening visit. These include information collection of past and current medical history, as well as previous/concomitant medications (between signing the ICF and the end of the screening period). The specific regimens, start and end times, and outcomes of all previous antitumor therapies (systemic or local, radiotherapy, chemotherapy, etc.) should be recorded.

- Physical examination and vital signs

Complete a comprehensive physical examination before dosing and at follow-up. Abnormal findings and new or worsening signs of physical examination should be recorded. A full physical examination is required for a single dose and prior to the start of the infusion for each cycle.

Vital signs examinations, including blood pressure, temperature, pulse rate and respiration, should be performed during the screening period, within 7 days prior to per dosing cycle, at the end of treatment, and at safety follow-up. Vital signs are evaluated within 60 minutes before the infusion of each administration and within 60 minutes after the completion of all therapeutic drugs. Repeat it if necessary.

- PS scoring

PS scoring is performed during the screening period, prior to each treatment cycle, at the end of the trial, and at the safety follow-up.

- Quality of life scoring (Annex VI)

EORTC QLQ-C30 and EORTC QLQ-LC13 scales are used for evaluation: EORTC QLQ-C30 is a core scale for all cancer patients, including functional dimensions (somatic, role, cognitive, emotional, and social functioning), symptom dimensions (fatigue, pain, nausea and vomiting), and general health/quality of life dimensions. EORTC QLQ-LC13. a subscale for lung cancer patients, evaluates disease-related symptoms (dyspnea, cough, hemoptysis, chest pain, arm/shoulder pain, and other pain). The two scales are assessed during the screening period, at each imaging assessment, at the time of withdrawal from the trial, and under the condition of unscheduled imaging assessments.

- 12-lead ECG

QTc is recorded during the screening period and before each cycle of dosing. Echocardiography will be examined when there are significant abnormalities in ECG or obvious cardiac symptoms.

- Laboratory tests

Laboratory tests include blood routine, urine routine, fecal routine and fecal occult blood test, blood biochemistry, immune safety, immune panel, blood/urine human chorionic gonadotropin (HCG), of which the specific details are listed in the plan table of the trial. The tests were checked within 7 days before each dose of ZKAB001/placebo except for virology. HCG is only examined during the screening period and at the time of withdrawal from the trial. The tests are allowed for retesting during screening period and only retested once.

- Immunogenicity test

See section 9.3.

- Biomarker test

No less than 5 white pieces of tumor tissue specimens are provided within 4 weeks prior to or after the first dose, acceptable for archived or freshly obtained within 6 months before the first dose, and less than 5 pieces need to be approved by the sponsor, all of which are used for PD-L1 expression detection and analysis

- Imaging examination

Imaging examinations should be performed every 6 weeks ± 7 days until progression or death during the screening period, after the start of treatment, regardless of the treatment is delayed or interrupted. Computed tomography (CT) is preferred for scans of the chest, abdomen, pelvis and lesions. Only if contrast-enhanced CT is contraindicated (e.g., contrast allergy), chest CT without contrast, with contrast-enhanced MRI or non-contrast CT for other sites. Magnetic resonance imaging (MRI) is preferred for brain imaging. During the screening period, a contrast-enhanced MRI of the brain (preferred) or contrast-enhanced CT scan as well as whole-body bone scan (ECT) are also performed, of which ECT must be performed within 42 days prior to the first dose. If bone metastases have been confirmed by PET-CT, ECT should not be repeated. ECT should be repeated only when CR is confirmed in the efficacy evaluation of the target lesions or there is suspicion of bone progression. The imaging examination methods of the same subject should be consistent throughout the trial period (except for change of examination methods due to contraindication to CT, acceptance of the results of PET-CT during the screening period whereas accepting the results of ECT at follow-up). A brain examination is required during the treatment period according to indication of a tumor in the brain in the screening period, or to confirm the occurrence of CR or PR due to clinical indications. Evaluation should also be performed when disease progression is suspected (e.g., worsening symptoms) and when participants withdraw from treatment (if the assessment has not been completed in the previous 4 weeks). Baseline imaging and efficacy assessment of imaging should be performed at the same study institute. Results of tests conducted at the same center within 28 days prior to receiving the first dose (brain results within 42 days) are acceptable.

Subjects with first documented remission (CR or PR) and initial documented radiographic progression requires an additional imaging confirmation at least 4 weeks later. If progression is not confirmed, treatment with study drug is continued and imaging evaluation is performed until progression again. Subjects who progress again must discontinue treatment. For those who discontinue treatment for reasons other than imaging progression, imaging evaluation can be performed according to the imaging evaluation timepoints until any of the following events occurs: initiation of new anti-tumor therapy, subjects’ unwillingness to continue the trial, loss to follow-up, and death.

## 8.6 Examinations at Screening Period and Baseline

Screening period is from day -28 to day 0, during which objectives of the study will be informed to potential patients. All the screening-related procedures will only be completed after the potential patients who are willing to participate in the study sign the ICF. No matter how the patients will be distributed for the subsequent treatments, the screening-related procedures are all the same. The following assessments and procedures may be completed during the screening period. Baseline data are recorded as the non-missing test value closest to the first dose. There is no need to repeat it at baseline for the examination during screening period within 1 week before the first dose:

1. Informed consent form.
2. Inclusion/exclusion criteria.
3. Demographics data: gender, height, age, weight.
4. Medical history.
5. Initial diagnosis and prior medications.
6. Vital signs, physical examination, PS scoring.
7. Blood/urine human chorionic gonadotropin (HCG), indicated only for childbearing potential women without hysterectomy surgeries, with a confirmed negative pregnancy test prior to dosing.
8. The blood routine includes white blood cell count, absolute neutrophil count, red blood cell count, hemoglobin, platelet count. Results within 7 days before the first administration of study drugs are accepted. Urine/fecal routine and fecal occult blood test include urine specific gravity, urine pH, urine protein, urine glucose, urine white blood cell, urine red blood cell. Results within 7 days before the first administration of study drugs are accepted.
9. Coagulation function at least includes international normalized ratio (INR), activated partial thromboplastin time (APTT), fibrinogen (FIB), thrombin time (TT). Results within 7 days before the first administration of study drugs are accepted. Examinations are performed during the screening period, at the time of withdrawal from the trial, and when clinically indicated during the study or at the discretion of the investigator.
10. Blood biochemistry includes total bilirubin, direct bilirubin or indirect bilirubin, alkaline phosphatase, aspartate aminotransferase, alanine aminotransferase, albumin, creatinine, urea/urea nitrogen, sodium, potassium, calcium, glucose. Results within 7 days before the first administration of study drugs are accepted.
11. Immunization panels include HBV, HCV, HIV tests. The results of the examination within 28 days before the first dose are accepted.
12. Immune safety analysis: thyroid-stimulating hormone, free T4 level.
13. ECG, results of the same site within 28 days prior to the first dose are accepted.
14. During the screening period and within 4 weeks after enrollment, no less than 5 white slices of tumor tissue specimens (old or fresh) should be provided, and less than 5 pieces must be approved by the sponsor to detect the PD-L1 expression level of tumor cells.
15. Imaging contrast CT or MRI results within 28 days prior to the first dose (brain and ECT within 42 days may be accepted).
16. Concomitant diseases and treatments.
17. Quality of life scoring.

## 8.7 Treatment and Follow-Up Examinations

The time window for each dose is calculated from the date of the first dose (3 weeks (-3~+7 days)), and the delayed injection management due to toxicity is described in Section 6.5.

Unless specified, the following examinations are performed before each treatment, and the baseline examination is used as the first pre-treatment examination. For multiple sets of data (e.g., vital signs, etc.), the results of closest time to medication should be recorded. The time window for all examinations should be within 7 days before the use of each cycle of medication, and re-examination should be carried out at any time when the investigator deems it necessary:

1. Vital signs, physical examination, physical condition (PS score);
2. Body weight, calculating body surface area;
3. Complete blood count；
4. Biochemical tests；
5. Immune safety analysis, checked every 6 weeks；
6. Urine/fecal routine and fecal occult blood test；
7. ECG；
8. Imaging examinations, every 6 weeks；
9. Concomitant diseases and treatments；
10. Quality of life scoring, frequently as same as imaging examinations;
11. Adverse events documents.

The drug can be administered after completing the pre-treatment examination, and the dosing schedule is described in Section 6.4 Dosing Schedule.

To confirm the safety of subjects at the end of treatment, an examination at end-of-trial (EOT) should be performed within 7 days after their last treatment/confirmed withdrawal from the study. No medication should be taken within 7 days prior to confirmation of termination of study treatment. Previous evaluations and examinations should not be repeated, and imaging should be performed if 4 weeks have passed since the last screening.

Subjects who withdraw from the study due to toxicity should have examinations including imaging every 6 weeks until disease progression or death is confirmed.

Safety follow-up period: within 90 days after the last dose, safety should be followed up every 30 ± 7 days. The first time for evaluation or examination should be returned to the hospital or in a local hospital. The examination at the end of the trial will be the first safety follow-up after the end of medication if more than one drug cycle (3 weeks) has passed since the last dose at the time of withdrawal from the study. Subsequent two telephone follow-up visits are conducted to collect information on survival status, adverse events, concomitant medications, and concomitant treatments. The follow-up will end until death, loss to follow-up, initiation of other anti-tumor therapy, or completion of all three times of safety follow-up.

Survival follow-up: After the termination of treatment and the end of safety follow-up, telephone follow-up is conducted every 30 days (±7 days) to collect the subject's survival information (date of death and cause of death) and information after the end of study treatment (including subsequent anti-tumor therapy) until death or lost to follow-up or the termination of study.

Please refer to the research plan table for the study diagram (appendix 5).

# 9. CLINICAL EVALUATIONS

Different from traditional chemotherapy or targeted drugs, the clinical efficacy (objective response) of drugs that activate the anti-tumor immune response has a delayed effect, typically occurring weeks or months after administration. During this time, some lesions appear to become larger (either due to true tumor growth or increased inflammatory infiltration), which is often interpreted as disease progression, and others appear to shrink or stabilize. However, with continuous treatment, the anti-tumor immune response matures, and all lesions may shrink or stabilize. In other words, it is possible to detect the initial significant radiographic progression, or the appearance of new lesions, or the shrinking of some lesions mixing with enlargement of some lesions (known as "mixed efficacy"). Therefore, in the case that subjects exhibit early tumor volume increase and significant early progression, if the clinical status is stable or improved, treatment with the study drug may be continued for one cycle until the next protocol-specified imaging evaluation timepoint and confirmed to be progressive. In this clinical study, if the subjects develop PD but clinically stable, they can continue study drug therapy.

In the absence of clinical deterioration, continuous administration of the study drug is allowed even if radiographic progression is detected until confirmed imaging deterioration of disease and further confirmed deterioration at the next successive imaging evaluation. The investigator should make a comprehensive clinical judgment to consider whether the subject is experiencing clinical deterioration and is unlikely to continue to benefit from treatment.

## 9.1 Efficacy Evaluation

Imaging evaluations of this study will be performed at each study center. IRC independently evaluates the efficacy endpoint such as PFS. Imaging evaluation of the study center is performed by the investigator. Each center should burn all imaging examination data related to efficacy evaluation into a CD-ROM archive and transmit it to IRC for evaluation.

Imaging experts from all centers and IRC evaluate the efficacy based on tumor imaging according to the RECIST v1.1.

1. Progression-free survival (PFS):

PFS is defined as the time from randomization until any documented progressive disease or death from any cause.

Evaluated according to RECIST v1.1, the analysis of the indicator includes the results of tumor evaluation during the study treatment and follow-up periods. The indicator appearing firstly is used for PFS analysis when patient has several indicators that can be judged as PD. Recurrence, new lesions, or death are considered to meet the endpoints. Disease is considered to progress if patient adopts other systemic anti-tumor therapies or therapies for the observed target lesions. The last time that the patient has not experienced disease progression will be used as censored data if disease progression or death doesn’t occur at the end of the study.

1. Overall survival (OS):

OS is defined as the time from randomization to death from any cause.

OS is recorded as data censor at the time of the last follow-up if participant is still alive at the last follow-up. For participants lost to follow-up, OS is recorded as data censor at the last time of confirmed survival before loss-to-follow if participant is lost to follow-up.

1. Disease control rate (DCR):

DCR is defined as the proportion of patients whose best overall response is CR, PR, and SD maintained for more than 4 weeks in patients with evaluable efficacy.

Best overall response refers to the best efficacy recorded by the investigator about the date of enrollment to disease progression objectively documented or to the initiation of subsequent anti-tumor therapy (whichever occurs first) in accordance with RECIST v1.1. For subjects who do not have disease progression or subsequent anti-tumor therapy, the best overall response will be determined based on all efficacy evaluations.

1. Objective response rate (ORR):

ORR is defined as the proportion of patients whose best overall response is CR and PR maintained for more than 4 weeks in patients with evaluable efficacy

1. Duration of response (DOR):

DOR is defined as the time from the first tumor evaluation as CR or PR to the first evaluation as PD or death from any cause for patients whose best overall response is CR or PR.

## 9.2 Safety Evaluation

Hematology and blood chemistry tests are performed during treatment and the results are reviewed before each dose (within 7 days). Any grade 3 or higher laboratory abnormalities or changes associated with possible immune-related adverse events (relative to disease progression) should be assessed risk for continuous treatment.

In addition, immune safety refers to the detection of changes in laboratory tests due to autoimmunity or other due to ZKAB001 stimulating the immune system. The generation of new reactivity is not necessarily related to clinical results, but safety monitoring is required in accordance with the protocol.

Safety evaluation includes all adverse events that occur throughout the trial from the first administration of the investigational drug to the end of safety follow-up, regardless of causal relationship with the investigational drugs. All adverse events (AE) and serious adverse events (SAE) will be classified and recorded according to the Common Terminology Criteria for Adverse Events (CTCAE) v5.0. Clinically significant symptoms, signs, and examinations must be documented in the EDC about the timing of occurrence, severity, relevance to the investigational drug, measures taken, prognosis, etc., for statistical analysis.

## 9.3 Immunogenicity Analysis

### 9.3.1 Time of Blood Sampling

According to the enrollment order, the first 100 subjects were collected for blood sample once before the administration of ZKAB001/placebo, after the administration of the first day of the first cycle (C1D1), and before the administration of C5D1 and C12D1, respectively. Blood samples are collected as close as possible to the initiation of reaction, the time of elimination, and approximately 30 days after the end of the reaction for pre- and post-immunogenicity analysis in case of infusion-related reaction. Subjects for blood sampling with transfusion reactions cannot be limited to the first 100 subjects.

### 9.3.2. Processing and Preservation of Blood Samples

At each of the timepoints above, about 3mL of venous blood is collected in the serum separation tube. The serum is evenly divided into 2 cryopreservation tubes (1 for immunogenicity [ADA] and 1 for backup), all of which are placed in a cryogenic freezer and stored at -60~-80 °C for 6 months and -20 °C for 1 month until they are transported to the central laboratory for testing. The specimens sent to the central laboratory will be destroyed by the central laboratory in accordance with the medical waste SOP with approval of the sponsor after the end of the test. The destruction report will be submitted to the research institution promptly.

### 9.3.3 Transportation of Blood Samples

Samples for testing are firstly sent out in a dry ice storage state. The back-up samples will be sent out after the bioanalytical laboratory confirms receipt of the samples for testing.

### 9.3.4 Testing of Blood Samples

Anti-drug antibody (ADA) will be tested for blood samples from subjects before and after dosing in this study.

# 10. REPORTING OF ADVERSE EVENTS

All AEs/SAEs occurring during the study period from the time of receipt of the investigational product to the end of the safety follow-up period , whether or not related to the investigational product, must be documented in detail in the medical history and entered on the AE/SAE page of the eCRF.

## 10.1 Adverse Event (AE)

"Adverse events" (AE) refers to all adverse medical events that occur after clinical study patients receive the investigational product. It can be manifested as symptoms, signs, diseases, or abnormal laboratory tests, but it may not be inferred to be related to the investigational product. Clear causality. Therefore, an adverse event may be any discomfort and unconscious signs (for example: abnormal laboratory test results), symptoms or diseases. According to the NCI CTCAE v5.0 criteria, all adverse events are to be followed up until the end of the safety follow-up period or until the event resolves, or returns to baseline levels, or until ≤ Grade 1 (CTCAEv5.0), or the subject is lost to follow-up or dies (or other plausible explanation).”

## 10.2 Serious Adverse Event (SAE)

“Serious adverse event” refers to an adverse medical event such as patient died, life-threatening, involved persistent or significant disability or incapacity, involved or prolonged inpatient hospitalization, congenital anomalies or birth defects, and other significant medical events that occur after the subject has received the investigational product.

1. A serious adverse event refers to an AE that causes one of following conditions:

- Patient died；
- Life threatening；
- Involved or prolonged inpatient hospitalization；
- Involved persistent or significant disability or incapacity；
- Birth defects / Congenital anomaly。
- Other: Medically important/significant: Medical and scientific judgment must be used to determine whether to expedite reporting of other conditions, such as significant medical events that may not be immediately life-threatening, fatal, or hospitalizing, but are also generally considered serious if medical measures are needed to prevent one of the above conditions from occurring. Examples include critical treatment in an emergency room or allergic bronchospasm at home, malaise or convulsions without hospitalization, development of drug dependence or addiction, etc.。

1. Hospitalization

Adverse events reported during the clinical study period that resulted in hospitalization or prolonged hospitalization were serious adverse events. Any initial hospitalization in a health care facility (even if the length of stay was less than 24 hours) met this criterion. In-hospital transfers to an acute/intensive care unit were also included。

Hospitalization does not include the following institutions：

- Rehabilitation center；
- Shelters；
- Respite care (e.g., giving a caretaker a temporary break)；
- Nursing facilities；
- Sanatorium；
- General emergency Hospitalization

1. A hospitalization or prolonged hospital stay is not a serious adverse event if it is not accompanied by an urgent clinical adverse event. Examples:

- Admission for a pre-existing condition not associated with a new adverse event and no worsening of the condition (e.g. a condition check due to persistent abnormalities in pre-treatment laboratory tests);
- Admission for social reasons (e.g. patient became homeless);
- Administrative reasons for admission (e.g., routine annual physical examination, convenience medication);
- Admissions designated or permitted by the protocol during the study period (e.g., hospitalization required or permitted by the study protocol);
- Self-hospitalization unrelated to an urgent adverse event (e.g., elective plastic surgery);
- Pre-planned treatments or surgical procedures noted in the baseline record for the benefit of the protocol as a whole and/or the individual subject;
- Admission solely for transfusion of blood products.

1. Disease Progression

Disease progression is defined as a worsening of the patient's condition caused by the primary tumor targeted by the study drug. Disease progression may be an increase in the severity of the studied disease and/or an increase in the symptoms of the disease; the presence of a new lesion relative to the primary tumor and/or the progression of a pre-existing lesion are considered to be disease progression. Disease progression is not reported as an AE; deaths, life-threatening, requiring hospitalization or prolonged hospitalization, resulting in permanent or severe disability/incapacity, resulting in congenital anomalies/birth defects, and other medically significant events due to signs and symptoms of disease progression are not reported on an accelerated basis as SAEs.

## 10.3 Adverse Event/Serious Adverse Event Relationship Assessment

The relationship of the adverse event to the investigational product will be assessed by the investigator and, to the extent possible, the basis for the judgment, in addition to a determination of causality to the investigational product, will be described as outlined below:

1. (Definitely) Related

There is evidence of administration of the test drug; the chronology of the adverse event in relation to the administration of the test drug is plausible; the occurrence of the adverse event is more plausibly explained by the test drug than by other causes; there is a positive withdrawal response; there is a positive repeat medication test; the pattern of the adverse event is consistent with prior knowledge of the medication or medications; and the influence of other confounding factors, such as pre-existing conditions, has been ruled out.

1. Probably related

There is no history of repeat drug use, the remainder is “definite”, or although there is a combination of drugs, the possibility that the combination of drugs led to the adverse reaction can be largely ruled out.

1. Possibly Related

There is evidence of administration of the test drug; the chronology of the adverse event in relation to the administration of the test drug is plausible; the adverse event may have been caused by the test drug or by other reasons, or the progression of the original disease cannot be ruled out.

1. Unlikely Related

Evidence of administration of test drug; adverse event is more likely to be due to other causes; withdrawal reaction is negative or equivocal; repeat drug test is negative or equivocal.

1. Not Related

The subject did not take the test drug; or the chronology of the occurrence of the adverse event in relation to the taking of the test drug is not plausible; or there is some other significant cause that could have contributed to the adverse event.

The study should also judge the occurrence of an AE/SAE to determine whether it is an immune-related adverse event.

## 10.4 Evaluation of Severity of Adverse Events

The intensity or severity of adverse events is categorized into grades 1 to 5:

- Grade 1 Mild: asymptomatic or mild; seen only clinically or diagnostically and requires no treatment;
- Grade 2 Moderate: requires minor, localized or non-invasive treatment; age-appropriate limitation of instrumental activities of daily living;
- Grade 3 Severe or medically significant but not immediately life-threatening; resulting in hospitalization or prolonged hospitalization; disabling; limitation of spontaneous activities of daily living;
- Grade 4 Life-threatening, requiring urgent treatment;
- Grade 5 AE-related death.

## 10.5 Suspected, Unexpected Serious Adverse Reactions（SUSAR）

SUSAR is an adverse event that simultaneously satisfies the criteria of relevant, serious, and unanticipated. Unintended, in which case, for a trial drug, means that the event is not listed in the investigator's brochure or that its nature or severity is not consistent with that described in the investigator's brochure. In the absence of an Investigator's Brochure, the event is inconsistent with the risk information described in the study plan or application materials.

Any SAE report received by the sponsor should be immediately analyzed and evaluated, including severity, relevance to the test drug, and whether it is an anticipated event. The sponsor should rapidly report the suspected and unanticipated serious adverse reactions (SUSAR) to all investigators participating in the clinical trial as well as to the clinical trial organization and the ethics committee; and the sponsor should rapidly report the suspected and unanticipated serious adverse reactions to the drug regulatory authority and the competent authority in charge of health care.

For all SUSARs occurring during clinical trials, rapid reporting should be made to the national drug review organization within the specified time limit. Rapid reporting starts on the date of clinical trial approval/start date of implied license from the National Drug Review Agency and ends on the date of the end of the last subject follow-up in the country. The occurrence of SUSAR after the end of the clinical trial or the end of the follow-up until the conclusion of the review and approval is obtained should also be subject to rapid reporting.

Duration of Rapid Reporting: For fatal or life-threatening SUSAR, the sponsor shall report as soon as possible after the first notification, but not more than 7 days, and report and complete the follow-up information within the following 8 days. For non-fatal or life-threatening SUSARs, sponsors should report as soon as possible, but no later than 15 days, after first notification (Note: the day the applicant is first notified is Day 0).

In the event of a SUSAR, to facilitate the determination of the relevance of the serious adverse event to the test drug, the sponsor will only “blind” individual cases. During the blinding process, only individual specialists (center principal investigator, group leader principal investigator, and sponsor PV) should blind individual cases, while the person who analyzes and describes the efficacy results should remain “blinded”. Through proper clinical trial design and management, the blinding of individual cases does not usually affect the conduct of the trial or the analysis of the final results.

## 10.6 Reporting of Serious Adverse Events

Except for serious adverse events that are not required to be reported immediately as specified in this trial protocol or other documents (e.g., Investigator's Brochure), the investigator should report all serious adverse events in writing to the Pharmacovigilance department of **Zhaoke (Guangzhou) Oncology Pharmaceutical Ltd.** via email (zhaokeoncologypv@leespharm.com) and send the SAE upon receipt of any updated information on the follow-up form to the department. If the SAE is judged to be causally related to the test drug, then SAEs occurring more than 90 days after the last dose are also subject to information collection and reporting. Serious adverse event reports and follow-up reports should indicate the subject's identification code from the clinical trial, rather than identifying information such as the subject's real name, citizenship number, and address.

For reports involving fatal events, the investigator should provide the sponsor and the ethics committee with other required information such as autopsy reports and final medical reports.

The investigator should sign and read the relevant safety information of the clinical trial provided by the sponsor in a timely manner after receiving it, and consider the treatment of the subjects, whether to adjust it accordingly, and communicate with the subjects as early as possible if necessary and should report the suspected and unintended serious adverse reactions provided by the sponsor to the ethics committee.

**Management of pregnancy events**

1）In the event that a pregnancy event occurs in a subject during the trial, the blinding should be urgently broken to obtain information about the subject's medication during the trial. The investigator should communicate scientifically and critically with the subject based on the dosing information to inform her/him of the possible effects and risks of the investigational drug on the pregnant woman and the fetus, and to leave it to the subject's discretion whether to terminate the pregnancy or to continue the pregnancy.

2）In the case of a female subject who becomes pregnant during the trial, the investigator should first terminate the clinical trial for that subject upon notification of the pregnancy event. If termination of pregnancy is required by the protocol, the subject should be notified at the earliest opportunity and the termination should be negotiated. If there is no scientific support for the necessity of termination, then the pregnancy needs to be followed closely until termination (induced abortion, spontaneous abortion, induced labor) or 28 days after delivery.

3）For male subjects whose sexual partner conceives during the trial, they will continue to participate in the clinical trial but will be required to report the pregnancy event and follow up with their partner to terminate the pregnancy or follow their partner's pregnancy through 28 days after delivery.

4）Within 24 hours of confirmation of a pregnancy event in the subject (or the subject's sexual partner), the investigator shall complete the Pregnancy Report Form Initial Report, as well as the Pregnancy Report Form Follow-Up Report that is required to be completed within 24 hours of each follow-up pregnancy, with the Pregnancy Report Form Initial/Follow-Up Report reported in accordance with the SAE Reporting process.

5）Upon receipt of the pregnancy report form, the sponsor's Pharmacovigilance Specialist maintains the report on a long-term basis in the case of an uneventful pregnancy; in the case of congenital anomalies or malformations of the fetus/newborn (SAEs), spontaneous abortion (SAEs), and termination of pregnancy for medical reasons (SAEs), the report is processed as an expedited report.

## 10.7 Follow-up of Adverse Events

All adverse events (AE/SAE) are to be followed up until the end of the safety follow-up period or until the event is resolved, or until it returns to baseline levels, or until it is ≤ Grade 1 (CTCAEv5.0), or until the subject is lost to a visit or dies (or other plausible explanations), in accordance with the NCI CTCAE v5.0 criteria.

The investigator should ask at each visit about adverse events that have occurred since the last visit and provide timely follow-up information based on the sponsor's challenge request. The principles for the collection and follow-up period for AEs/SAEs occurring after the subject's last dose of medication during the end phase of the study are as follows:
1）non-study drug-related AE

Recorded until the end of the safety follow-up period or the initiation of new antitumor therapy (whichever is reached first); follow-up until the end of the safety follow-up period.
2）drug-related AE

Records up to the end of the safety follow-up period; follow-up until disappearance, remission, or to baseline level, or ≤ grade 1, or reaching steady state, or with reasonable explanation (e.g., loss to follow-up, death) (whichever is reached first).
3）non-study drug-related SAE

Recorded up to the end of the safety follow-up period or initiation of new antitumor therapy (whichever is reached first) up to the end of the safety follow-up period
4）drug-related SAE

Recorded and followed up until disappearance, remission, or to baseline level, or ≤ grade 1, or reaching steady state, or with reasonable explanation (e.g., lost to visit, death).

# 11. DATA MANAGEMENT

## 11.1 Data Management Tools

Electronic Data Capture (EDC) system for drug clinical trials was used in this study for study data collection and management.

## 11.2 Electronic Case Report Form (eCRF) Construction

Before the start of the study, the data manager and relevant technicians created an eCRF on the EDC platform based on the paper case report form that had been developed for the investigators to fill in. The data administrator and the project leader carefully check the eCRF and the paper case report form and confirm the consistency. The entire EDC platform, including the database, eCRF, and the instant logic verification program, should be tested several times to make sure there are no errors before use.

At the same time, the data administrator should work with the research team to formulate the data range checking and logic checking contents according to the range and interrelationship of each index value in the case report form. The corresponding computer program should be written on the EDC platform to achieve the purpose of immediate logical checking when filling in the eCRF and controlling the filling errors of the eCRF. At the same time, it also prepares the background logic verification program, so as to carry out the background logic verification on the export line of the filled data.

## 11.3 Privilege Management

The system account administrator creates accounts and grants different privileges to access the EDC system according to the different roles of researcher, monitor, data entry personnel, data management personnel, auditor, etc. For example, the researcher of each center can only see the content of their own center. Data entry personnel have both data entry, modification, and challenge resolution privileges; researchers have modification, browsing, challenge resolution, and auditing privileges; principal investigators have electronic signature privileges in addition to researcher privileges; CRAs have browsing, sending/closing of challenges, and SDV verification privileges; and data managers have browsing, sending/closing of challenges, data freezing, locking, and DM verification privileges.

## 11.4 Data Input

The EDC system was used in this study, eliminating the need for a paper CRF to database data entry process. Prior to the start of the study, the data manager should fully understand the contents of the protocol and case report form, standardize the coding of the individual fill-in items and set up the EDC backend database. Once the study begins, the investigator, or assistant investigator, loads the source data into the eCRF in a timely, complete, correct, and authentic manner.

## 11.5 Data Questions and Answers

After the data are entered into the EDC database, the system will verify the data according to the Edit Check built in the Data Verification Planner, and any questionable data will be automatically sent out for system questioning; the Supervisor confirms on the EDC platform that all case report forms are filled in correctly and completely and are in agreement with the original information. If there are errors and omissions, queries can be raised online at any time, and the investigator or entry personnel will be asked to confirm and answer in eCRF in a timely manner, and the erroneous data will be modified if necessary until the challenge is resolved. the EDC platform keeps traces of all data changes, including the person who made the changes and the time of the changes. The data administrator verifies the data online or after exporting it through the EDC platform, and when a question is found, the researcher or entrant is asked to answer it. When the answer fails to resolve the query, the data manager and the supervisor can re-question the data point, and all records are kept in the EDC database.

## 11.6 Data Locking and Output

After each subject has completed the trial and all data have been reviewed for accuracy, the data will be locked by the data manager according to the database locking protocol. Any modification after database locking should be requested, discussed and signed by the sponsor, investigator, data manager and statistician before execution. After all data are locked, the data manager will output them to the statistician for statistical analysis. At the end of the study, the electronic data modification trace form on the eCRF and EDC platform is printed or archived as needed. The investigator shall keep the clinical trial data until two years after the trial drug is approved for marketing. The sponsor should keep the clinical trial data until five years after the approval of the trial drug for marketing. However, this information should be retained for a longer period of time if required by current regulations or the agreement with the sponsor. The sponsor will notify the investigator in writing when the data are no longer required to be maintained.

## 11.7 Provisions for Source Data

In accordance with (ICH) E6, related regulations, and the requirements of the research organization for the protection of subjects' personal information, each research center is required to maintain appropriate treatment and scientific records related to this study. As part of Sponsor-funded or participating research, each Research Center shall allow inspection (and, if permitted by law, copying) of clinical records by Sponsor-authorized representatives and regulatory agencies for the purpose of conducting quality reviews, audits, and evaluations of safety, progress, and data validity. Who is authorized to access these records should also be described in this section.

Raw data are all the information necessary to reconstruct and evaluate a clinical study and are the original records of clinical findings, observations, or other activities. Examples of these original documents and data records include, but are not limited to: hospital records, clinical and Office software charts, laboratory records, memoranda, subject questionnaires or evaluation checklists, pharmacy dispensing records, audio recordings of counseling sessions, recorded data from automated instruments, photocopied or transcribed records that are verified and determined to be accurate and complete, microfilm, photographic negatives, microfiche, or diskettes, x-ray films, CT or MRI, ECG records, subject diary cards, and subject documents and records maintained in participating pharmacies, laboratories, and medical technology departments.

All such documents must be labeled with at least the subject number and the date the procedure was performed. If possible, the medical evaluation of these records should be documented as necessary with the documents signed by the investigator with name and date. The investigator is responsible for ensuring that source data are accurate, legible, contemporaneous, original, and attributable, regardless of whether the data are handwritten on paper or entered electronically.

# 12. STATISTICAL ANALYSIS

## 12.1 General Considerations

The development of a Statistical Analysis Plan (SAP), which comprehensively indicates the statistical methodology (including tables, graphs, and tabular forms) for all aspects of the planned analyses, will be conducted by the sponsor's assignor. This SAP will be developed as part of the clinical study pre-reporting of results and approved prior to the final lock-up of data. Additional unplanned analyses may be required after all planned analyses have been completed. Any unplanned analyses will be identified in the CSR.

If not otherwise indicated, all data will be summarized with descriptive statistics according to random groups and using appropriate statistics based on the type of data: mean, standard deviation, median, minimum and maximum values for continuous variables, and frequency and percentage for categorical variables.

## 12.2 Analytic Populations

The following analytic populations will be used as the analysis of the study data:

Full Analysis Set (FAS): all subjects who are randomized and receive the trial drug at least once.

Per Protocol Set (PPS): a subset of subjects defined in the FAS set, excluding subjects with significant protocol deviations that are judged to have a significant impact on the outcome.

Safety Analysis Set (SS): all subjects who were randomized and received the trial drug at least once and for whom safety evaluation data were available.

## 12.3 Analysis of Efficacy

The efficacy analysis will be based on FAS and PPS, with FAS as the primary and PPS as the secondary.

1）Primary efficacy

The primary endpoint of this study is OS. the Kaplan-Meier method was used to estimate the median OS and its corresponding confidence interval, to estimate the survival rate of OS at different time points (including but not limited to the OS rate at 1 year and 2 years), and to plot the corresponding survival curves. A stratified Log-Rank test will be used to compare OS between the two groups, with stratification factors considered as for randomized stratification factors. In addition, a COX proportional wind model will be used to estimate the hazard ratio (HR) of the test group relative to the control group and its corresponding confidence interval. The treatment group and randomization stratification factors will be considered as independent variables in the model.

2）Secondary efficacy

For the secondary endpoint objective remission rate (ORR), the number and proportion of subjects summarizing ORR by treatment group and their 95% confidence interval (based on the Clopper-Person method). A stratified Cochran-Mantel-Haenszel (CMH) method was used to compare the difference in ORR between the test group and the control group, and P values were calculated. The difference in ORR rate between the two groups based on the normal distribution approximation and its 95% confidence interval. Disease control rate (DCR) will be analyzed in the same way as ORR. PFS and DoR will be analyzed using similar analytical methods as for the primary study endpoint OS. For quality of life scores, descriptive statistics of the total and dimensional scores at baseline and at each evaluation time point, as well as the change values relative to baseline, will be used to compare the differences in overall survival domain scores between the two groups by analysis of covariance.

## 12.4 Analysis of Safety

Treatment Emergent Adverse Event (TEAE) will be coded using the MedDRA dictionary. The incidence of TEAEs will be summarized separately by treatment group according to system organ class and preferred terminology. The incidence of all TEAEs, adverse reactions, TEAEs leading to discontinuation and termination of therapy, TEAEs leading to death, and SAEs will be summarized. The severity and relevance to treatment of TEAEs were also further summarized according to the most severe criterion. Descriptive statistics were used to summarize other safety indicators. Baseline was defined as data from the most recent test prior to the first dose. Laboratory tests: descriptive summaries of laboratory test values and analysis of post-treatment abnormalities in the form of pre- and post-treatment cross-tabulations. Vital signs: Mean, maximum, minimum, median, and standard deviation were used to describe measurements and changes at each visit. Physical examination and lead ECG were analyzed descriptively.

## 12.5 Interim Analysis

The interim analysis of this trial will be performed by the Independent Data Monitoring Committee (IDMC). Details will be specified in the IDMC charter. Interim analyses are planned to be conducted at 60% data maturity, with validity thresholds calculated using the O'Brien Fleming Type 1 error consumption function and the actual number of OS events at a nominal test level of 0.0038, and the number of OS events reaching 369 OS events for the final analysis at a nominal test level of 0.0238. The final analysis will be conducted if the final event count or interim analysis event count is slightly different from that planned. analysis, if the final event count or the interim analysis event count differs slightly from the plan (possibly caused by multiple events occurring on the same day that the event count is reached), appropriate adjustments will be made to the category I error boundaries for the final analysis. The study reaches the OS primary endpoint if the OS rejects the original hypothesis at the interim analysis, or if the original hypothesis is not rejected at the interim analysis but is rejected at the final analysis.

The detailed timing of the interim analyses and the details will be specified or adjusted in the interim analysis plan.

# 13. QUALITY CONTROL AND QUALITY ASSURANCE

During this study, regular on-site monitoring visits to the study hospitals will be conducted by clinical supervisors assigned by the sponsor to ensure that all elements of the study protocol are strictly adhered to and that the study data are correctly completed.

1. Participants in the study must undergo uniform training and standardization of record-keeping and judgment criteria.
2. The entire clinical research process should be carried out in strict accordance with the protocol. 3. the investigator should follow the case study protocol.
3. The investigator should record the contents of the CRF truthfully, in detail and carefully according to the requirements for filling in the case report form, so as to ensure that the contents of the case report form are true and reliable.
4. The abnormal judgment standard of laboratory tests shall be based on the normal reference range of the examination unit.
5. All observations and findings in the clinical study should be verified to ensure the reliability of the data, and to ensure that the conclusions in the clinical study are derived from the original data. There are appropriate data management measures in both the clinical study and data processing stages.
6. Active measures shall be taken against possible fallout, and the rate of case fallout shall be controlled within 20%.

# 14. ETHICAL PRINCIPLES

## 14.1 General Considerations

In compliance with the requirements of the Declaration of Helsinki, 《Provisions of Drug Registration》, the ICH guidelines and other regulations and guidelines, the trial protocol, the drawn up informed consent and other information given to the patients must be submitted to the Ethics Committee (EC) of the unit in charge of the clinical study for review before this trial is conducted. The sponsor must obtain a signed and dated written approval from the EC prior to the start of the trial, and any amendments to the protocol and informed consent form must be approved by this committee.

The trial must obtain informed consent from the subjects and sign the informed consent form before proceeding, the rights and interests of the subjects should be ensured during the trial, and the confidentiality of the subjects' information should be observed.

## 14.2 Informed Consent Process and Signing of Informed Consent Form

Informed consent begins before the patient agrees to participate in the trial and continues throughout the trial until withdrawal. The risks and possible benefits of participating in the trial will be discussed in detail and fully with the subject and his/her family. Subjects should review the written informed consent form carefully and ask questions before signing. The investigator will explain the clinical trial to the subject and answer any questions the subject may have. Subjects may discuss this trial with their proxies and think thoroughly before agreeing to participate in the trial. Subjects may begin participation in the trial only after they have signed the informed consent form. A copy of the informed consent form will be retained by the subject. Subjects may withdraw their consent at any time during the course of the clinical trial and should be protected that the quality of their medical care will not be affected in any way.

Amendments to the protocol involving trial eligibility (inclusion exclusion criteria), or where there are significant safety information additions and revisions, the informed consent form should be updated and informed consent should be given to investigators who are participating in the trial after review and approval by the Ethics Committee.

# 15. DATA REETENTION AND SUMMARIZATION

The investigator must keep a transcript of the original data for each subject. (Primary data include: a copy of the informed consent form signed by the subject with the trial number and trial name, laboratory data, ECG, etc.) Any information that appears on the case report form should be able to be sourced in these primary sources.

All raw data information related to this study should be kept by the research unit in accordance with the requirements of China's “Code of Practice for the Quality Management of Drug Clinical Trials” until 5 years after the drug has been approved for marketing, at which time the sponsor will notify the investigator/research organization that it will no longer keep records related to the trial. The basic information includes: (1) Ethics Committee's approval of the trial protocol and all protocol revisions; (2) all original data; (3) Case Report Form (CRF); (4) Informed Consent Form; (5) any other trial-related documents.

# 16. RESPONSIBILITIES OF ALL PARTIES AND REGULATIONS ON PUBLICATIONS

The investigator shall keep all information provided by the sponsor in strict confidence, and also require other trial participants and the ethics committee to take the same confidentiality measures. The information provided by the sponsor to the investigator shall not be disclosed to others without the written permission of the company.

All information and results of the trial will remain the property of the investigator and the sponsor. Any article that wishes to be published should be submitted to the sponsor prior to dispatch, who will review it for accuracy, confirm that the confidential information has not been compromised, and add any relevant information.

# REFERENCES

- 1. Howlader N, Noone AM, Krapcho M, et al. SEER Cancer Statistics Review, 1975-2014, based on November 2016 SEER data submission, posted to the SEER web site, April 2017. Bethesda, MD: National Cancer Institute; 2017.
  2. Govindan R, Page N, Morgensztern D, et al. Changing epidemiology of small-cell lung cancer in the United States over the last 30 years:analysis of the surveillance, epidemiologic, and end results database. J Clin Oncol 2006;24:4539-4544.
  3. Jett JR, Schild SE, Kesler KA, Kalemkerian GP. Treatment of small cell lung cancer: Diagnosis and management of lung cancer, 3rd ed: American College of Chest Physicians evidence-based clinical practice guidelines. Chest 2013;143:e400S-419S.
  4. Micke P, Faldum A, Metz T, et al. Staging small cell lung cancer: veterans administration lung study group versus international association for the study of lung cancer-what limits limited disease? Lung Cancer, 2002, 37(3): 271-276.
  5. Schneider BJ, Saxena A, Downey RJ. Surgery for early-stage small cell lung cancer. J Natl Compr Canc Netw 2011;9:1132-1139.
  6. Ignatius Ou SH, Zell JA. The applicability of the proposed IASLC staging revisions to small cell lung cancer (SCLC) with comparison to the current UICC 6th TNM Edition. J Thorac Oncol 2009;4:300-310.
  7. Stinchcombe TE, Gore EM. Limited-stage small cell lung cancer: current chemoradiotherapy treatment paradigms. Oncologist 2010;15:187-195.
  8. People's Medical Publishing House. Chinese Society of Clinical Oncology, Primary Lung Cancer Diagnosis and Treatment Guidelines, 2018 edition.
  9. Evans WK, Shepherd FA, Feld R, et al. VP-16 and cisplatin as first-line therapy for small-cell lung cancer. J Clin Oncol 1985;3:1471-1477.
  10. Rossi A, Di Maio M, Chiodini P, et al. Carboplatin- or cisplatin-based chemotherapy in first-line treatment of small-cell lung cancer: the COCIS meta-analysis of individual patient data. J Clin Oncol 2012;30:1692-1698.
  11. Schiller JH, Adak S, Cella D, et al. Topotecan versus observation after cisplatin plus etoposide in extensive-stage small-cell lung cancer:E7593--a phase III trial of the Eastern Cooperative Oncology Group. J Clin Oncol 2001;19:2114-2122.
  12. von Pawel J, Jotte R, Spigel DR, et al. Randomized phase III trial of amrubicin versus topotecan as second-line treatment for patients with small-cell lung cancer. J Clin Oncol 2014;32:4012-4019.
  13. Ettinger DS, Jotte R, Lorigan P, et al: Phase II study of amrubicin as second-line therapy in patients with platinum-refractory small-cell lung cancer. J Clin Oncol 28:2598-2603, 2010.
  14. Hellmann MD, Ott PA, Zugazagoitia J, et al. Nivolumab (nivo) ±ipilimumab (ipi) in advanced small-cell lung cancer (SCLC): First report of a randomized expansion cohort from CheckMate 032. J Clin Oncol, 2016, 35(suppl): abstr 8503.
  15. Lesterhuis WJ, Nowak AK, Lake RA. Immune stimulatory features of classical chemotherapy. In: Cancer Immunotherapy – Immune Suppression and Tumor Growth. Prendergast GC, Jaffee EM (Eds). Academic Press, UK, 395–416 (2013 ).
  16. Brown JS, et al. Combining DNA damaging therapeutics with immunotherapy: more haste，less speed. J Br J Cancer, 2018,118（3）：312-324.
  17. Pfirschkec, et al. Iimmunogenic chemotherapy sensitize tumors to checkpoint blockade therapy. J Immunity. 2016，44（2）: 343-354.
  18. McGranahanN, et al. Clonal neoantigens elicit T Cell immunoreactivity and sensitivity to immune checkpoint blockade. J Science, 2016,351(6280):1463-1469.

# Appendix 1: Method of Staging Small Cell Lung Cancer

——《Chinese Society of Clinical Oncology (CSCO) Small Cell Lung Cancer Diagnosis and Treatment Guidelines 2020》

**AJCC (8th edition) TNM staging method combined with the American Veterans Lung Cancer Association (VALG) Stage II staging method:**

Limited stage: Stage I to III (any T, any N, M0), can be safely treated with a defined radiation dose. T3-4 that cannot be included in a tolerable radiotherapy schedule due to multiple nodules in the lungs or the tumor/nodule volume being too large is excluded.

Extensive stage: AJCC (8th edition) Stage IV (any T, any N, M1a/b), or T3-4 that cannot be included in a tolerable radiotherapy schedule due to multiple nodules in the lungs or the tumor/nodule volume being too large.

# Appendix 2: ECOG Performance Status Evaluation

| Grade | ECOG |
| --- | --- |
| 0 | Fully active, able to carry on all pre-disease performance without restriction |
| 1 | Restricted in physically strenuous activity but ambulatory and able to carry out work of a light or sedentary nature, e.g., light housework, office work |
| 2 | Ambulatory and capable of all selfcare, confined to bed or chair more than 50% of walking hours |
| 3 | Capable of only limited selfcare, confined to bed or hair more than 50%of waking hours |
| 4 | Completely disabled. Cannot carry on any selfcare. Totally confined to bed or chair |
| 5 | Dead |

# Appendix 3: Response Evaluation Criteria in Solid Tumors (RECIST1.1) (Revision)

**Definitions**

| Measurable lesions | Lesions that can be accurately measured in at least one dimension, the longest diameter to be recorded as ≥20 mm, or spiral CT ≥10mm by clinical exam |
| --- | --- |
| Non-measurable lesions | All other lesions (including small lesions, i.e., the longest diameter< 20 mm or spiral CT <10 mm by clinical exam). Bone lesions, leptomeningeal disease, ascites, pleural/pericardial effusions, lymphangitis cutis/pulmonitis, inflammatory breast disease, and abdominal masses and cystic lesions that cannot be diagnosed by imaging are considered as non-measurable. |
| Target  Lesions | >1 Measurable lesions under baseline assessment, all lesions should be recorded and measured, and up to a maximum of 5 lesions total (not more than 2 per organ). Target lesions should be selected on the basis of size and measurable repeatable assessment.  The sum of the lengths of all target lesions is used as the reference baseline for effective remission records. Lymph nodes with a short diameter ≥ 15 mm in CT scans can be used as pathologically significant measurable target lesions, and the total number of target lesions can be included in the evaluation of curative effect.  Criteria for mitigation:   - Complete response（CR）：the disappearance of all targets lesions, the short axis value of any pathological lymph node (including target lesion or non target lesion) must be less than 10mm. - Partial response（PR）：using the sum of diameter as a reference, the total diameter of target lesions is reduced by at least 30%. - Progressive disease（PD）：Based on the minimum value of the total diameter of all target lesions measured during the experimental study, the total diameter should be increased by at least 20% (take the baseline as the reference if the baseline measurement value is the smallest value), and the absolute value of the total diameter increase must be greater than 5mm , or the appearance of new lesions - Stable disease（SD）：Taking the minimum value of the sum of the diameters of the target lesions measured in the study as a reference, the reduction did not reach PR or increased but did not reach PD. PD needs to be confirmed in this study, unless the patient has a rapidly progressing clinical deterioration. |
| Non-target Leision | Except target leision, any other lesions, including pathological lymph nodes is considered as a non-target lesions without further assessments, but it should be recorded in the baseline assessment. For example, "existence", "absence" or in rare cases "clear progress". Extensive target lesions can be recorded with target organs (such as a large number of enlarged pelvic lymph nodes or large-scale liver metastases)  Complete response（CR）：the disappearance of all non target lesions, and the level of tumor markers returned to normal levels. All lymph nodes are non-pathological (ie, the short axis value is less than 10 mm).  Non-complete response/ non-progressive disease: the continuous existence of >1 non-target lesions, and/or the level of tumor markers continues to be higher than normal.  Progressive disease（PD）：One or more new lesions and/or existing non-target lesions have clearly progressed. PD needs to be confirmed in this study, unless the patient has a rapidly progressing clinical deterioration. |
| Best Overall Response Rate | From the beginning of treatment to disease progression or recurrence, the smallest measurement value measured, and the valus is verified at least 4 weeks later, CR or PR is the best confirmed therapeutic effect. The measurement is carried out with a ruler or a measuring instrument and recorded in international units. |

- Total Evaluation

| Target Lesion | Non-target Lesion | New lesion | Total evaluation |
| --- | --- | --- | --- |
| CR | CR | No | CR |
| CR | Non- CR/non- PD | No | PR |
| CR | Cannot be assessd | No | PR |
| PR | Non- PD or cannot be fully assessed | No | PR |
| SD | Non- PD or cannot be fully assessed | No | SD |
| Cannot be fully assessed | Non- PD | No | NE |
| PD | any | Yes/ No | PD |
| any | PD | Yes/ No | PD |
| any | any | Yes | PD |

Remarks：CR，complete response；PR，compete response；SD，stable disease；PD，progressive disease；NE，not evaluated

- Efficacy Evaluation

Patients evaluated as CR or PR should repeat the evaluation again at least 4 weeks later. In this study, the time interval for evaluating tumor efficacy meets this standard. Patients who are evaluated as SD should repeat the evaluation again after a specific interval written in the protocol (generally no less than 6-8 weeks).

| Total efficacy at the first time point | Total efficacy at subsequent time points | Best Overall Response Rate |
| --- | --- | --- |
| CR | CR | CR |
| CR | PR | SD, PD或PR^a^ |
| CR | SD | If SD lasts for sufficient time, it considered as SD. Otherwise, considered as PD |
| CR | PD | If SD lasts for sufficient time, it considered as SD. Otherwise, considered as PD |
| CR | NE | If SD lasts for sufficient time, it considered as SD. Otherwise, considered as NE |
| PR | CR | PR |
| PR | PR | PR |
| PR | SD | SD |
| PR | PD | If SD lasts for sufficient time, it considered as SD. Otherwise, considered as PD |
| PR | NE | If SD lasts for sufficient time, it considered as SD. Otherwise, considered as NE |
| NE | NE | NE |

Remarks：CR，complete response；PR，partial response；SD，stable disease；PD，progressive disease；NE，not evaluated

1. If CR is reached at the first time point, any diseases observed at subsequent time points, even if it meets the PR criteria relative to the baseline, it will be assessed as PD (because the disease reappears after reaching CR). The Best Overall Response Rate depends on the time that a SD lasts for. Sometimes, it is evaluated as CR during assessment at the first time point, but in fact there are small lesions. Therefore, it should be evaluated as the a PR instead of CR at the first time point. In this cicumstance, the originally evaluated CR will be changed to PR and the best response will be PR.

# Appendix 4: Management of Immune Checkpoint Inhibitor-Related Toxicity

-- From the 2019 Guidelines of Chinese Society of Clinical Oncology (CSCO) “Management of Immune Checkpoint Inhibitor-Related Toxicity”

Principles of Toxicity Grading Management

| Grading a, b | Hospitalization level | Glucocorticoid c-h | Other immunosuppressive agents | ICI treatment |
| --- | --- | --- | --- | --- |
| G1 | No need for hospitalization | Not recommended | Not recommended | Continue |
| G2 | No need for hospitalization | Topical or systemic glucocorticoids, oral prednisone, 0.5~1mg/ (kg·d) | Not recommended | Suspend k |
| G3 | Hospitalization required | Systemic glucocorticoid therapy, oral prednisone or intravenous methylprednisolone 1~2 mg/(kg·d) | Consider administration under the guidance of a specialist for patients whose symptoms have not been alleviated after 3 to 5 days of glucocorticoid treatment. | Discontinue and discuss resumption of ICIs based on the patient's risk/benefit ratio |
| G4 | Hospitalization required, consider admission to the intensive care unit (ICU) | Systemic glucocorticoid therapy, intravenous methylprednisolone 1~2mg/(kg-d) for 3 consecutive days, gradually reduce to 1mg/(kg-d) if the symptoms are relieved, then gradually reduce to discontinuation over about 6 weeks. | Consider administration under the guidance of a specialist for patients whose symptoms have not been alleviated after 3 to 5 days of glucocorticoid treatment. | Permanently discontinue |

1. All patients should be informed of the potential toxicity of ICIs prior to initiating treatment. In the event of toxicity, patients should report suspicious symptoms to the treatment team (medical staffs) in time and seek prompt medical attention for evaluation, examination, and diagnosis on an outpatient or inpatient basis, so that the medical staffs can take prompt action to prevent further deterioration of the toxicity. At present, ICIs have just been approved for anti-tumor therapy in China, and the majority of medical professionals specialized in oncology have insufficient experience in understanding and dealing with toxicity. In addition, some patients may receive infusion in non-specialized institutions, so it is also necessary to raise the awareness of toxicity among emergency physicians and community physicians.
2. Clinical management of toxicity is based on the principle of grading. The Common Terminology Criteria for Adverse Events (CTCAE_4.03) developed by the National Cancer Institute (NCI) at the National Institutes of Health (NIH) grades the terminology and severity of adverse events; however, the use of CTCAE for grading toxicity has certain limitations, sometimes underestimating or overestimating the incidence and severity of toxicity [18]. The guidelines classify toxicity into five grades: G1, mild toxicity; G2, moderate toxic; G3, severe toxicity; G4, life-threatening toxicity; G5, toxicity-related death; basically corresponding to CTCAE_4.03 classification of adverse reactions.
3. Toxicity management relies heavily on the use of glucocorticoids, which are commonly used immunosuppressive agents. The use of glucocorticoids, as well as the dose and dosage form of the hormone used, should be judged clinically based on the toxicity grade. Oral glucocorticoid preparations are generally preferred for G1 to G2 toxicity. However, sometimes high-dose intravenous glucocorticoids are preferred due to the aggressive nature of severe toxicity, such as cardiac, pulmonary, hepatic, and neurologic toxicity [19]. The glucocorticoids should be used timely, and delays in their use (>5 days) can affect the final management of some ICIs-related toxicities, such as diarrhea/colitis [20]. To prevent recurrence of toxicity, glucocorticoids should be tapered (>4 weeks, sometimes 6-8 weeks or longer), especially in the treatment of immune-related pneumonia and hepatitis.
4. There is a lack of definitive clinical evidence on whether the use of glucocorticoids for toxicity management has adverse effects on ICIs efficacy, but prolonged, higher-dose use of glucocorticoids may have negative effects on treatment. In general, the use of glucocorticoids prior to ICI therapy to prevent infusion reactions is not recommended.
5. Long-term use of glucocorticoids may increase the risk of opportunistic infections. Targeted prophylaxis against pneumocystis pneumonia is recommended for patients on long-term glucocorticoid therapy (prednisone >20 mg/day for more than 4 weeks). Antifungal agents (e.g., fluconazole) should also be considered for the prevention of mycotic pneumonia in patients using glucocorticoids for longer periods of time (prednisone >20 mg/day for more than 6-8 weeks).
6. Concomitant treatment with proton pump inhibitors or H2 receptor blockers is recommended for patients on long-term glucocorticoid therapy who are taking non-steroid anti-inflammatory drugs (NSAIDs) or anticoagulants.
7. Patients on long-term glucocorticoid therapy are at risk of developing osteoporosis, and oral supplementation with vitamin D and calcium tablets is recommended to prevent osteoporosis.
8. It should be noted that hypothyroidism and other endocrine toxicities (e.g., diabetes mellitus) do not require glucocorticoid therapy, but alternative hormone therapy is recommended.
9. Other immunosuppressive agents may be considered in cases where glucocorticoids are ineffective, including TNF-α inhibitors (e.g., infliximab), mycophenolate, tacrolimus, and biological immune agents such as antithymocyte globulin (ATG).
10. Short-term use of high-potency topical glucocorticoids is recommended for rashes, rather than long-term use of low-potency glucocorticoids.
11. If only cutaneous or endocrine symptoms are present, ICIs therapy may be continued.

**Management of common toxicities**

Cutaneous toxicity

| Grade | Description | | Class I recommendation |
| --- | --- | --- | --- |
| Maculopapular rash/dermatitis | | | |
| G1 | Macules/papules covering <10% body surface area(BSA) with or without symptoms (e.g., pruritus, burning, tightness) | | Continue ICI  Topical emollients  Oral antihistamines  Medium-potency topical glucocorticoids |
| G2 | Macules/papules covering 10%-30% BSA with or without symptoms (e.g., pruritus, burning. tightness); limiting instrumental ADL | | Topical emollients  Oral antihistamines  High-potency topical glucocorticoids and/or prednisone, 0.5~1mg/(kg·d) |
| G3 | Macules/papules covering >30% BSA with or without associated symptoms (e.g, erythema, purpura, excoriation); limiting self-care ADL | | Hold ICI  High-potency topical glucocorticoids, prednisone, 0.5~1mg/(kg·d) (If no improvement, increase dose to 2 mg/(kg·d)) |
| Pruritus | | | |
| G1 | Mild or localized | | Continue ICI  Oral antihistamines  Medium-potency topical glucocorticoids |
| G2 | Intense or widespread: intermittent; skin changes from scratching (e.g, edema, papulation, excoriation, lichenification, oozing/crusts); limiting instrumental ADL | | Continue ICI with intensive antipruritic therapy  High-potency topical glucocorticoids  Oral antihistamines  Consider discontinuation in some severe patients |
| G3 | Intense or widespread: constant; limiting self-care ADL or sleep | | Hold ICI  Prednisone/methylprednisolone, 0.5~1mg/(kg·d)  Oral antihistamines  GABA agonist (gabapentin, pregabalin)  Consider aprepitant or omalizumab for refractory pruritis (e.g. elevated blood IgE levels) |
| Bullous dermatitis /Stevens-Johnson syndrome (SJS)/toxic epidermal necrolysis (TEN) | | | |
| G1 | | Asymptomatic, blisters covering <10% BSA | Hold ICI  High-potency topical glucocorticoids |
| G2 | | Blisters covering 10%-30% BSA with pain; limiting instrumental ADL | Hold ICI until toxicity < grade 1  Prednisone/methylprednisolone, 0.5~1mg/(kg·d)  Blood routine, hepatic and renal function, electrolyte, CRP tests |
| G3 | | Blisters covering >30% BSA; limiting self-care ADL; SJS or TEN | Permanently discontinue ICI  Prednisone/methylprednisolone, 1~2mg/(kg·d)  Hospitalize, ICU or burn ward indicated  Dermatology, ophthalmology, urology consultation  Blood routine, hepatic and renal function, electrolyte, CRP, complement and other relevant inflammatory factor tests |
| G4 | | Blisters covering >30% BSA; disturbance of water and electrolyte; fatal SJS or TEN |  |

Endocrine toxicity

| Grade | Description | | Class I recommendation |
| --- | --- | --- | --- |
| Hypothyroidism | | | |
| G1 | Asymptomatic; clinical or diagnostic observations only; intervention not indicated | | Continue ICI |
| G2 | Symptomatic; thyroid replacement indicated; limiting instrumental ADL | | Continue ICI  Supplement thyroxine if TSH is elevated (>10μIU/ml) |
| G3 | Severe symptoms; limiting self-care ADL; hospitalization indicated | |  |
| G4 | Life-threatening consequences; urgent intervention indicated | |  |
| Hyperthyroidism | | | |
| G1 | Asymptomatic; clinical or diagnostic observations only; intervention not indicated | | Continue ICI, oral propranolol, metipranolol, or atenolol for symptomatic relief if symptomatic.  Review TFTs after 4-6 weeks; no further treatment is needed if relieved; Consider 4-hour or 24-hour iodine uptake tests to identify hyperthyroidism or toxic diffuse goiter (Graves' disease) if TSH remains below normal levels and free T4/total T3 is elevated. |
| G2 | Symptomatic; thyroid suppression therapy indicated; limiting instrumental ADL | |  |
| G3 | Severe symptoms; limiting self-care ADL; hospitalization indicated | |  |
| G4 | Life-threatening consequences; urgent intervention indicated | |  |
| Hypophysitis | | | Hold ICI until relief of acute symptoms  Start methylprednisolone/prednisone 1~2mg/(kg·d) if symptomatic  Consider appropriate hormone replacement therapy according to clinical indications |
| Primary adrenal insufficiency | | | Hold ICI  Administer corticosteroids to avoid adrenal crisis before initiating other hormone replacement therapy  Steroid replacement therapy: hydrocortisone, 20mg am, 10mg pm, then titrate slowly according to symptoms; or prednisone at an initial dose of 7.5mg or 10mg, then reduced to 5mg once/day as appropriate, and fludrocortisone at a dose of 0.1mg once every other day; then increase or decrease the dosage based on blood pressure, symptoms, lower extremity edema and laboratory test results; hospitalize and start high/stress doses of steroids if hemodynamically unstable; supply a large amount of fluid for patients with severe symptoms (hypotension) (e.g., normal saline usually needs to be >2L). |
| Hyperglycemia (preferred fasting glucose) | | | |
| G1 | | Fasting glucose <8.9mmol/L | New-onset hyperglycemia <11.1 mmol/L and/or history of type 2 diabetes without diabetic ketoacidosis (DKA): continue ICI, monitor blood glucose dynamically during treatment, adjust diet and lifestyle, and administer medications according to the corresponding guidelines.  New-onset fasting glucose >11.1 mmol/L or random glucose >13.9 mmol/L or history of type 2 diabetes with fasting/random glucose >13.9 mmol/L:  (1) Improve blood pH, basic metabolic panel, urine or plasma ketone bodies, β-hydroxybutyric acid, etc.  (2) Measure C-peptide, anti-glutamic acid decarboxylase (GAD) antibodies, and anti-islet cell antibodies, if urine or blood ketone body/anion gap is positive.  (3) Negative DKA: manage as “new-onset hyperglycemia <11.1 mmol/L”  (4) Positive DKA: hold ICI, hospitalize, seek endocrinology consultation and manage DKA according to institutional guidelines, and administer insulin under the direction of the inpatient care team and/or an endocrinologist. |
| G2 | | Fasting glucose 8.9~13.9mmol/L |  |
| G3 | | Fasting glucose 13.9~27.8mmol/L, hospitalization indicated |  |
| G4 | | Fasting glucose>27.8mmol/L; life-threatening consequences |  |

Liver toxicity

| Grade | Description | Class I recommendation |
| --- | --- | --- |
| G1 | AST, ALT<3×ULN; total bilirubin <1.5×ULN | Continue ICI |
| G2 | AST, ALT 3~5 ×ULN  total bilirubin 1.5~3 ×ULN | Hold ICI therapy  Oral prednisone 0.5~1mg/kg with 4 week taper if liver function improves  Resume ICI (Class II recommendation) when prednisone is tapered to ≤10 mg/day (toxicity grade ≤1) |
| G3 | AST, ALT 5~20×ULN; total bilirubin 3~10×ULN | G3: discontinue ICI  Resume ICI when prednisone is tapered to ≤10 mg/day (toxicity grade ≤1) |
| G4 | AST, ALT >20×ULN; total bilirubin >10×ULN | G4: permanently discontinue ICI therapy; start intravenous methylprednisolone 1~2mg/kg, or equivalent dose of oral prednisone after the hepatic toxicity is reduced to grade 2, and taper slowly over at least 4 weeks; consider mycophenolate mofetil (500~1,000mg, 2 times/day) if no improvement in hepatic function after 3 days; infliximab is not recommended. |

Gastrointestinal toxicity (diarrhea/colitis)

| Grade | | Description | Class I recommendation |
| --- | --- | --- | --- |
| G1 | | Asymntomatic; clinical or diagnostic observations only (Grade 1 diarrhea frequency ≤ 4/day) | Laboratory tests: blood routine, hepatic and renal function, electrolyte, thyroid function  Fecal tests: microscopy for leukocytes, eggs, parasites, cultures, viruses, clostridium difficile, cryptosporidium, and cultures of drug-resistant pathogens  Continue ICI  Oral rehydration and use antidiarrheal drugs for symptomatic management if necessary  Avoid high fiber/lactose diets |
| G2 | | Abdominal pain; mucus or blood in stool (Grade 2 diarrhea frequency 4-6/day) | Laboratory and fecal tests as above  Gastrointestinal X-ray for signs of colitis  Schedule a colonoscopy and biopsy  Hold ICI  Start hormone therapy without waiting for a colonoscopy  Oral prednisone, 1mg/( kg·d)  Increase dose to 2 mg/(kg·d) if no improvement or exacerbation with 48-72 hours of hormone therapy; consider infliximab |
| G3~G4 | G3: Severe abdominal pain; change in bowel habits; medical intervention indicated; peritoneal signs (Grade 3 diarrhea frequency ≥ 7x/day)  G4: Life-threatening consequences; urgent intervention indicated | | Laboratory and fecal tests as above  Abdominal and pelvic enhanced CT is recommended for signs of colitis  Schedule a colonoscopy and biopsy  Daily reexamination of blood routine, hepatic and renal function, electrolytes, and CRP  Dietary guidance (fasting, liquid diet, total parenteral nutrition)  G3: hold ICI; G4: permanently discontinue ICI  Intravenous methylprednisolone 2mg/( kg·d)  Start hormone therapy without waiting for a colonoscopy  Consider infliximab while continuing hormone therapy if no improvement or exacerbation with 48 hours of hormone therapy  Consider vedolizumab if infliximab resistant |

Pulmonary toxicity (pneumonia)

| Grade | Description | Class I recommendation |
| --- | --- | --- |
| G1 | Asymptomatic; confined to a single lobe or <25% of lung parenchyma | Baseline examination: chest CT, blood oxygen saturation, blood routine, hepatic and renal function, electrolytes, TFTs, ESR, lung function  Consider reexamination of chest CT and lung function in 3~4 weeks  Follow up closely and resume treatment if imaging improves  Escalate treatment regimen and hold ICI if imaging progresses  Continue treatment and follow up closely until new symptoms appear if no change in imaging |
| G2 | New or worsening symptoms, including: shortness of breath, cough, chest pain, fever, and hypoxia; involving multiple lobes and 25%-50% of lung parenchyma, affecting daily life and requiring pharmacological intervention. | Perform a high-resolution CT of the chest, blood routine, hepatic and renal function, electrolytes, and lung function analysis  Hold ICI until ≤G1  Intravenous methylprednisolone 1~2mg/( kg·d); if symptoms improve after 48~72 hours of treatment, taper the hormone by 5~10mg per week for 4 to 6 weeks; if symptoms do not improve, treat as G3~G4 reactions; if infection cannot be completely excluded, consider empiric anti-infective therapy; repeat chest CT in 3-4 weeks.  If clinical symptoms and imaging abnormalities resolve to ≤G1, immune agents can be used after evaluation. |
| G3 | Severe new-onset symptoms involving all lobes or >50% of lung parenchyma, limiting self-care ADL; oxygen and hospitalization indicated | Perform a high-resolution CT of the chest, blood routine, hepatic and renal function, electrolytes, and lung function analysis  Permanently discontinue ICI, hospitalize  Consider empiric anti-infective therapy if infection has not been completely ruled out; consider respiratory or infectious disease consultation if necessary  Intravenous methylprednisolone 2mg/(kg·d), pulmonary ventilation as appropriate; if clinical symptoms improve after 48 hours of hormone therapy, continue treatment until symptoms improve to ≤G1, then taper the dose over 4-6 weeks; if no significant improvement, consider intravenous infliximab (5 mg/kg), or mycophenolate mofetil (1 g/time, bid), or intravenous immunoglobulin h |
| G4 | Life-threatening respiratory compromise, ARDS; urgent intervention indicated (eg, intubation) |  |

Rheumatoid/skeletal muscle toxicity

Rheumatoid arthritis

| Grade | Description | Class I recommendation |
| --- | --- | --- |
| G1 | Mild pain with inflammatory symptoms (improvement of symptoms with movement or heat), erythema, or joint swelling | Continue ICI  NSAIDs (e.g., naproxen 0.5g BID for 4-6 weeks)  If NSAIDs ineffective, consider prednisone 10-20 mg daily for 2-4 weeks; if no improvement in 2-4 weeks, escalate to grade 2 management |
| G2 | Moderate pain associated with signs of inflammation, erythema, or joint swelling; limiting instrumental ADL | Hold ICI  Prednisone 0.5mg/(kg·d) for 4-6 weeks; if symptoms do not improve, escalate to grade 3 management  Consider endocrinology consultation if symptoms do not improve after 4 weeks |
| G3 | Severe pain associated with signs of inflammation, erythema or joint swelling; irreversible joint damage; disabling; limiting self-care ADL | Hold or permanently discontinue ICI  Prednisone 1mg/(kg·d) for 4-6 weeks  Consider endocrinology consultation if symptoms do not improve in 2 weeks  Consider additional immunosuppression (e.g methotrexate, sulfasalazine, leflunomide) |

Myositis or myalgia

| Grade | Description | Class I recommendation |
| --- | --- | --- |
| G1 | Mild symptoms with or without pain | Continue ICI  Comprehensive assessment of the patient's muscle strength  Monitor creatine kinase, aldolase, aminotransferase (AST, ALT), and lactate dehydrogenase (LDH)  Consider glucocorticoid therapy if creatine kinase levels are elevated with weakened muscle strength  Administer acetaminophen or NSAIDs for analgesia after relevant contraindications have been ruled out |
| G2 | Moderate symptoms with or without pain; limiting instrumental ADL | Hold ICI until associated symptoms are controlled, creatine kinase has returned to normal levels and prednisone dose <10mg  Administer NSAIDs for analgesia after relevant contraindications have been ruled out  Administer 0.5~1 mg/(kg·d) prednisone (or equivalent dose of other drug) if creatine kinase is ≥3 times the upper limit of normal |
| G3 | Severe symptoms with or without pain; limiting self-care ADL | Hold ICI until G1  Consider hospitalization  Consider endocrinology or neurology consultation  Start 1mg/(kg·d) methylprednisolone (or equivalent dose of other drugs) |

# Appendix 5: Study Diagram

| Visits  Items | Screening period^1^ | Baseline^2^ | Dosing period (3 weeks (D-3~D+7)) | | Follow-up period | | |
| --- | --- | --- | --- | --- | --- | --- | --- |
|  |  |  |  |  | End visit^3^ | Safety follow-up^4^ | Survival follow-up^5^ |
| Study period (day) | D-28~D1 | D-7~D1 | Within 7 days of each cycle | Within -7 days of every 2 weeks | Within 7 days of last dose/withdrawal | +30 days (±7 days) after last dose | +30 days of previous follow-up (±7 days) |
| Informed consent | **×** |  |  |  |  |  |  |
| Decision of inclusion and exclusion criteria | **×** |  |  |  |  |  |  |
| Demographic/medical history/history of tumor treatment^6^ | **×** |  |  |  |  |  |  |
| Tumor diagnosis and stage, including tumor tissue specimens^7^ | **×** |  |  |  |  |  |  |
| Blood/urine HCG^8^ | **×** |  |  |  | **×** |  |  |
| Vital signs, physical examination^9^ | **×** | **×** | **×** |  | **×** | **×** |  |
| PS scoring^10^ | **×** | **×** | **×** |  | **×** | **×** |  |
| Imaging^11^ | **×** |  |  | **×** | **×** |  |  |
| ECG^12^ | **×** |  | **×** |  | **×** | **×** |  |
| Blood routine^13^ | **×** | **×** | **×** |  | **×** | **×** |  |
| Coagulation function^14^ | **×** |  |  |  | **×** |  |  |
| Blood biochemistry^15^ | **×** | **×** | **×** |  | **×** | **×** |  |
| Immune safety test^16^ | **×** |  |  | **×** | **×** | **×** |  |
| HBsAg, HCV-Ab (or RNA), HIV antibody^17^ | **×** |  |  |  |  |  |  |
| HBV-DNA (Optional)^18^ | **×** |  |  | **×** |  |  |  |
| Urine/fecal routine and fecal occult blood test^19^ | **×** | **×** | **×** |  | **×** | **×** |  |
| Survival follow-up |  |  |  |  |  | **×** | **×** |
| Expression of PD-L1^20^ | **×** |  |  |  |  |  |  |
| Blood sampling for immunogenity^21^ | **×** |  |  |  |  |  |  |
| Quality of life scoring^22^ | **×** |  |  | **×** | **×** |  |  |
| Concomitant medications, adverse events^23^ | **×** | | | | | |  |

1. In addition to the tumor imaging and other examinations established within the specified time limit before the first dose, the written informed consent must be obtained before the start of any clinical study procedure. Subjects who have failed previous screening are allowed to be screened again, and at the time of re-screening, they must re-sign the informed consent form and re-register to obtain a new subject number.
2. Baseline data are recorded as the non-missing test value closest to the first dose. There is no need to repeat it at baseline for the examination during screening period within 1 week before the first dose.
3. To confirm the safety of subjects at the end of treatment, an examination at end-of-trial (EOT) should be performed within 7 days after their last treatment/confirmed withdrawal from the study. No medication should be taken within 7 days prior to confirmation of termination of study treatment. Previous evaluations and examinations should not be repeated, and imaging should be performed if 4 weeks have passed since the last screening.
4. Within 90 days after the last dose, safety should be followed up every 30 ± 7 days. The first time for evaluation or examination should be returned to the hospital or in a local hospital. The examination at the end of the trial will be the first safety follow-up after the end of medication if more than one drug cycle (3 weeks) has passed since the last dose at the time of withdrawal from the study. Subsequent two telephone follow-up visits are conducted to collect information on survival status, adverse events, concomitant medications, and concomitant treatments. The follow-up will end until death, loss to follow-up, initiation of other anti-tumor therapy, or completion of all three times of safety follow-up.
5. After the termination of treatment and the end of safety follow-up, telephone follow-up is conducted every 30 days (±7 days) to collect the subject's survival information (date of death and cause of death) and information after the end of study treatment (including subsequent anti-tumor therapy) until death or lost to follow-up or the termination of study.
6. These include information collection of past and current medical history, as well as previous/concomitant medications (between signing the ICF and the end of the screening period). The specific regimens, start and end times, and outcomes of all previous antitumor therapies (systemic or local, radiotherapy, chemotherapy, etc.) should be recorded.
7. The histological diagnosis, time of first diagnosis, time of progression or recurrence are recorded. These includes specimen type and acquisition method, pathological classification, secondary staging, and TNM staging.
8. Indicated only for childbearing potential women without hysterectomy surgeries. Blood/urine HCG pregnancy test is required at the screening period and at the end of the test.
9. Vital signs examinations, including temperature, pulse rate, respiration, systolic and diastolic blood pressure in the resting state. Vital signs are evaluated within 60 minutes before the infusion of each administration and within 60 minutes after the completion of all therapeutic drugs. Abnormal findings and new or worsening signs of physical examination should be recorded. A full physical examination is required for a single dose and prior to the start of the infusion for each cycle. Height is only measured during the screening period. Weight is measured during the screening period and prior to each cycle of treatment, but the frequency of measurements can be increased as needed.
10. PS scoring is performed during the screening period, prior to each treatment cycle, at the end of the trial, and at the safety follow-up.
11. Imaging examinations should be performed every 6 weeks ± 7 days until progression or death during the screening period, after the start of treatment, regardless of the treatment is delayed or interrupted. Contrast-enhanced computed tomography (CT) is preferred for scans of the chest, abdomen, pelvis and lesions. Only if contrast-enhanced CT is contraindicated (e.g., contrast allergy), chest CT without contrast, with contrast-enhanced MRI or non-contrast CT for other sites. Magnetic resonance imaging (MRI) is preferred for brain imaging. The imaging examination methods of the same subject should be consistent throughout the trial period (mode and contrast, except for change of examination methods due to contraindication to CT, acceptance of the results of PET-CT during the screening period whereas accepting the results of ECT at follow-up). During the screening period, contrast-enhanced MRI of the brain (preferred) or contrast-enhanced CT scan as well as whole-body bone scan (ECT) are also performed (if bone metastases have been confirmed by PET-CT, ECT should not be repeated). Results of tests conducted at the same center within 28 days prior to receiving the first dose (brain and ECT results within 42 days) are acceptable
12. ECT should be repeated only when CR is confirmed in the efficacy evaluation of the target lesions or there is suspicion of bone progression. A brain examination is required during the treatment period according to indication of a tumor in the brain in the screening period, or to confirm the occurrence of CR or PR due to clinical indications. Evaluation should also be performed when disease progression is suspected (e.g., worsening symptoms) and when participants withdraw from treatment (if the assessment has not been completed in the previous 4 weeks). Baseline imaging and efficacy assessment of imaging should be performed at the same study institute. Lesions that are not specified should be performed simultaneously in the subsequent follow-up.

Results of tests conducted at the same center within 28 days prior to receiving the first dose (brain and ECT results within 42 days) are acceptable.

1. QTc is recorded during the screening period and before each cycle of dosing. Echocardiography will be examined when there are obvious cardiac symptoms. Results at the same center within 28 days prior to treatment.
2. The blood routine includes white blood cell count, absolute neutrophil count, red blood cell count, hemoglobin, platelet count. It is tested within 7 days prior to each administration of ZKAB001/placebo. Results within 7 days before the first administration of study drugs are accepted.
3. Coagulation function at least includes international normalized ratio (INR), activated partial thromboplastin time (APTT), fibrinogen (FIB), thrombin time (TT). Results within 7 days before the first administration of study drugs are accepted. Examinations are performed during the screening period, at the time of withdrawal from the trial, and when clinically indicated during the study or at the discretion of the investigator.
4. Blood biochemistry includes total bilirubin, direct bilirubin or indirect bilirubin, alkaline phosphatase, aspartate aminotransferase, alanine aminotransferase, albumin, creatinine, urea/urea nitrogen, sodium, potassium, calcium, glucose. It is tested within 7 days prior to each administration of ZKAB001/placebo. Results within 7 days before the first administration of study drugs are accepted.
5. It includes thyroid-stimulating hormone and free T4 level. Results within 7 days before the first administration of study drugs are accepted. It is tested every 6 weeks.
6. It includes HBV, HCV, and HIV tests. Results within 28 days before the first administration of study drugs are accepted. Positive result of HIV/HCV during the screening period will be excluded according to the exclusion criteria.
7. Patients with positive HBsAg require a comprehensive HBV-DNA test to rule out active phase of hepatitis B (unless HBV-DNA titer < 500 IU/mL or copy number <1000 copies/mL after antiviral therapy). HBV-DNA testing is repeated every 2 cycles in positive patients. Antiviral therapy should be scheduled based on HBV-DNA results.
8. Urine routine includes urine specific gravity, urine pH, urine protein, urine glucose, urine white blood cell and urine red blood cell. It is tested within 7 days prior to each administration of ZKAB001/placebo. Results within 7 days before the first administration of study drugs are accepted. Fecal routine includes fecal occult blood test.
9. No less than 5 white slices of tumor tissue specimens (old or fresh) should be provided, and less than 5 pieces must be approved by the sponsor to detect the PD-L1 expression level of tumor cells.
10. According to the enrollment order, the first 100 subjects were collected for blood sample once before the administration of ZKAB001/placebo, after the administration of the first day of the first cycle (C1D1), and before the administration of C5D1 and C12D1, respectively. Blood samples are collected as close as possible to the initiation of reaction, the time of elimination, and approximately 30 days after the end of the reaction for pre- and post-immunogenicity analysis in case of infusion-related reaction. Subjects for blood sampling with transfusion reactions cannot be limited to the first 100 subjects.

At each of the timepoints above, about 3mL of venous blood is collected in the serum separation tube. The serum is evenly divided into 2 cryopreservation tubes (1 for immunogenicity [ADA] and 1 for backup), all of which are placed in a cryogenic freezer and stored at -60~-80 °C for 6 months and -20 °C for 1 month until they are transported to the central laboratory for testing.

1. EORTC QLQ-C30 and EORTC QLQ-LC13 scales are evaluated during the screening period, at each imaging assessment, at the time of withdrawal from the trial, and under the condition of unscheduled imaging assessments.
2. Adverse events that occur after signing informed consent but prior to administration of study drug should be documented in the medical history/current medical conditions sections of the CRF.

# Appendix 6: EORTC QLQ-C30+LC13 Quality of Life Score

1. Aronson NK, et al.The European Organization for Research and Treatment of Cancer (EORTC) modular approach to quality of life assessment in oncology. Int J Ment Health 1994;23:75–96
2. Bergman, B, et al.The EORTC QLQ-LC13: a modular supplement to the EORTC Core Quality of Life Questionnaire (QLQ-C30) for use in lung cancer clinical trials. EORTC Study Group on Quality of Life. European Journal of Cancer 1994;30a(5): 635-642

| **We are interested in learning some information about you and your health. Please answer all of the following questions independently and circle the answer that best describes you. There are no “right” or “wrong” answers. The information you provide will be kept strictly confidential.** | |
| --- | --- |
| 1. Do you have any trouble doing strenuous activities,like carrying a heavy shopping bag or  a suitcase? | □1-Not at all □2-A Little  □3-Quite a Bit □4-Very much |
| 2. Do you have any trouble taking a long walk? | □1-Not at all □2-A Little  □3-Quite a Bit □4-Very much |
| 3. Do you have any trouble taking a short walk  outside of the house? | □1-Not at all □2-A Little  □3-Quite a Bit □4-Very much |
| 4. Do you have to stay in a bed or a chair for  most of the day? | □1-Not at all □2-A Little  □3-Quite a Bit □4-Very much |
| 5. Do you need help with eating, dressing, washing yourself or using the toilet? | □1-Not at all □2-A Little  □3-Quite a Bit □4-Very much |
| **DURING THE PAST WEEK:** | |
| 6. Are you limited in any way in doing either  your work or daily activities? | □1-Not at all □2-A Little  □3-Quite a Bit □4-Very much |
| 7. Are you limited in any way in hobbies and leisure activities? | □1-Not at all □2-A Little  □3-Quite a Bit □4-Very much |
| 8. Were you short of breath? | □1-Not at all □2-A Little  □3-Quite a Bit □4-Very much |
| 9. Have you had pain? | □1-Not at all □2-A Little  □3-Quite a Bit □4-Very much |
| 10. Did you need to rest? | □1-Not at all □2-A Little  □3-Quite a Bit □4-Very much |
| 11. Have you had trouble sleeping? | □1-Not at all □2-A Little  □3-Quite a Bit □4-Very much |
| 12. Have you felt weak？ | □1-Not at all □2-A Little  □3-Quite a Bit □4-Very much |
| 13. Have you lacked appetite? | □1-Not at all □2-A Little  □3-Quite a Bit □4-Very much |
| 14. Have you felt nauseated？ | □1-Not at all □2-A Little  □3-Quite a Bit □4-Very much |
| 15. Have you vomited？ | □1-Not at all □2-A Little  □3-Quite a Bit □4-Very much |
| 16. Have you been constipated？ | □1-Not at all □2-A Little  □3-Quite a Bit □4-Very much |
| 17. Have you had diarrhea？ | □1-Not at all □2-A Little  □3-Quite a Bit □4-Very much |
| 18. Were you tired？ | □1-Not at all □2-A Little  □3-Quite a Bit □4-Very much |
| 19. Did pain interfere with your daily activies？ | □1-Not at all □2-A Little  □3-Quite a Bit □4-Very much |
| 20. Have you had difficulty in concentrating  on things, like reading a newspaper or watching  television? | □1-Not at all □2-A Little  □3-Quite a Bit □4-Very much |
| 21. Did you feel tense? | □1-Not at all □2-A Little  □3-Quite a Bit □4-Very much |
| 22. Did you worry? | □1-Not at all □2-A Little  □3-Quite a Bit □4-Very much |
| 23. Did you feel irritable? | □1-Not at all □2-A Little  □3-Quite a Bit □4-Very much |
| 24. Did you feel depressed? | □1-Not at all □2-A Little  □3-Quite a Bit □4-Very much |
| 25. Have you had difficulty remembering things? | □1-Not at all □2-A Little  □3-Quite a Bit □4-Very much |
| 26. Has your physical condition or medical  treatment interfered with your family life? | □1-Not at all □2-A Little  □3-Quite a Bit □4-Very much |
| 27. Has your physical condition or medical  treatment interfered with your social activities? | □1-Not at all □2-A Little  □3-Quite a Bit □4-Very much |
| 28. Has your physical condition or medical  treatment caused you financial difficulties? | □1-Not at all □2-A Little  □3-Quite a Bit □4-Very much |
| **FOR THE FOLLOWING QUESTIONS PLEASE CIRCLE THE NUMBER BETWEEN 1 AND 7 THAT BEST APPLIES TO YOU** | |
| 29. How would you rate your overall physical condition during the past week? | □1（Very poor） □2 □3 □4 □5 □6 □7（Excellent） |
| 30. How would you rate your overall quality of life during the past week? | □1（Very poor） □2 □3 □4 □5 □6 □7（Excellent） |
| **Patients sometimes experience the following clinical symptoms. Please indicate the extent to which you have experienced these clinical symptoms or problems in the past week, circling the answer that best applies to you.** | |
| **DURING THE PAST WEEK:** | |
| 31. How much did you cough? | □1-Not at all □2-A Little  □3-Quite a Bit □4-Very much |
| 32. Did you cough blood? | □1-Not at all □2-A Little  □3-Quite a Bit □4-Very much |
| 33. Were you short of breath when you rested? | □1-Not at all □2-A Little  □3-Quite a Bit □4-Very much |
| 34. Were you short of breath when you walked? | □1-Not at all □2-A Little  □3-Quite a Bit □4-Very much |
| 35. Were you short of breath when you climbed stairs? | □1-Not at all □2-A Little  □3-Quite a Bit □4-Very much |
| 36. Have you had a sore mouth or tongue? | □1-Not at all □2-A Little  □3-Quite a Bit □4-Very much |
| 37. Have you had trouble swallowing? | □1-Not at all □2-A Little  □3-Quite a Bit □4-Very much |
| 38. Have you had tingling hands or feet? | □1-Not at all □2-A Little  □3-Quite a Bit □4-Very much |
| 39. Have you had hair loss? | □1-Not at all □2-A Little  □3-Quite a Bit □4-Very much |
| 40. Have you had pain in your chest? | □1-Not at all □2-A Little  □3-Quite a Bit □4-Very much |
| 41. Have you had pain in your arm or shoulder? | □1-Not at all □2-A Little  □3-Quite a Bit □4-Very much |
| 42. Have you had pain in other parts of your body? | □1-Not at all □2-A Little  □3-Quite a Bit □4-Very much |
| If yes, where | ________ |
| 43. Did you take any medicine for pain? | □1-No □2-Yes |
| If yes, how much did it help? | □1-Not at all □2-A Little  □3-Quite a Bit □4-Very much |

# Appendix 7: Body Surface Area Calculation

——HU Yongmei, WU Xiaoluo, HU Zhihong, et al. A study on the formula of human body surface area in China[J]. Journal of Physiology,1999, 51(1):4.

Body surface area（m^2^）=0.0061×height（cm）+ 0.0124×weight (kg)-0.0099（unisex）
