# Supplementary material for: A multicenter, randomized, double-blind, placebo-controlled phase 3 study of Socazolimab or placebo combined with carboplatin and etoposide in the first-line treatment of extensive-stage small cell lung cancer
Source: Signal Transduct Target Ther. 2025 Jan 13;10:28. doi: 10.1038/s41392-024-02115-5 (PMC11725569; doi:10.1038/s41392-024-02115-5)
Supplement: Supplementary file 3 — Supplementary Materials [file 41392_2024_2115_MOESM3_ESM.docx]

Supplementary Materials for

**A multicenter, randomized, double-blind, placebo-controlled phase 3 study of Socazolimab or placebo combined with carboplatin and etoposide in the first-line treatment of extensive-stage small cell lung cancer**

Zhiwei Chen, Jianhua Chen, Dingzhi Huang, Wei Zhang, Lin Wu, Tienan Yi, Qiming Wang, Liang Han, Liping Tan, Yinyin Li, Zhihong Zhang, Na Li, Jie li, Tongmei Zhang, Ying Hu, Hongmei Sun , Youhua Wu, Zhiyong He, Runxiang Yang, Peng Cheng, Xingya Li, Jianhua Shi, Guohua Yu, Daiyuan Ma, Benjamin Xiaoyi Li, Xiangrong Dai, Michael Wong, Yujie Li MM, Xiaohui Yu, Shun Lu,for the Socazolimab Study Group

Correspondence to: shunlu@sjtu.edu.cn

**This document includes :** Supplementary Tables 1 to 4

**Supplementary table 1**: Subsequent anticancer therapies

|  | **Socazolimab plus EC group**  **(N = 248)，n (%)** | **Placebo plus EC group**  **(N = 248)，n (%)** |
| --- | --- | --- |
| At least one subsequent anticancer therapy | 188 (75·8) | 209 (84·3) |
| Therapy type |  |  |
| Radiotherapy | 58 (23·4) | 67 (27·0) |
| Surgery | 2 (0·8) | 3 (1·2) |
| Chemotherapy | 149 (60·1) | 161 (64·9) |
| Target therapy | 46 (18·5) | 64 (25·8) |
| Chemotherapy plus target therapy | 6 (2·4) | 8 (3·2) |
| Immunotherapy | 59 (23·8) | 54 (21·8) |
| Chemotherapy plus immunotherapy | 43 (17·3) | 26 (10·5) |
| Traditional Chinese medicine therapy | 29 (11·7) | 45 (18·1) |
| Chemotherapy plus traditional Chinese medicine therapy | 2 (0·8) | 3 (1·2) |
| Others | 9 (3·6) | 13 (5·2) |

**Supplementary table 2**: The treatment-emergent adverse events overview.

|  | **Socazolimab plus EC group**  **(N = 249) n (%)** | **Placebo plus EC group**  **(N = 247) n (%)** |
| --- | --- | --- |
| Treatment-emergent adverse events (TEAE)^a^ | 247 (99·2) | 244 (98·8) |
| Related to any component of the trial regimen (TRAE) | 246 (98·8) | 243 (98·4) |
| Related to socazolimab or placebo | 193 (77·5) | 174 (70·4) |
| TEAE of grade 3 or above | 209 (83·9) | 199 (80·6) |
| TRAE of grade 3 or above | 200 (80·3) | 187 (75·7) |
| TRAE of grade 3 or above related to socazolimab or placebo | 88 (35·3) | 68 (27·5) |
| TEAE according to the dose of carboplatin |  |  |
| TEAE of grade 3 or above in Carboplatin≥750mg^b^ | 24 (80·0) | 23 (67·6) |
| TEAE of grade 3 or above in Carboplatin＜750mg^b^ | 193 (79·1) | 177 (74·7) |
| TEAE according to age |  |  |
| TEAE of grade 3 or above in≥65 years^b^ | 108 (90·0) | 83 (85·6) |
| TEAE of grade 3 or above in＜65 years^b^ | 101 (78·3) | 116 (77·3) |
| Immune-related adverse events (irAE) | 48(19·3) | 24 (9·7) |
| irAE of grade 3 or above | 14 (5·6) | 2 (0·8) |
| Infusion reaction or allergy | 8 (3·2) | 4 (1·6) |
| Infusion reaction or allergy of grade 3 or above | 1 (0·4) | 0 |
| TEAE leading to termination of treatment | 15 (6·0) | 9 (3·6) |
| TRAE leading to termination of treatment | 12 (4·8) | 7 (2·8) |
| TRAE leading to termination of treatment related to socazolimab or placebo | 10 (4·0) | 5 (2·0) |
| Serious adverse events (SAE) | 105 (42·2) | 82 (33·2) |
| SAE related to any component of the trial regimen | 90 (36·1) | 64 (25·9) |
| SAE related to socazolimab or placebo | 38 (15·3) | 24 (9·7) |
| TEAE leading to death | 4 (1·6) | 5 (2·0) |
| TRAE leading to death | 3 (1·2) | 4 (1·6) |
| TRAE leading to death related to socazolimab or placebo | 3 (1·2) | 2 (0·8) |

1. Adverse events of any cause (safety set).
2. The percentage was calculated based on the number of patients in each treatment group of the corresponding population in the safety set.

Abbreviations: TEAE = Treatment-emergent adverse events; TRAE = Treatment-relatedt adverse events; irAE = Immune related adverse events; SAE = Serious adverse events.

**Supplementary table 3**: The immune related adverse events

|  | **Socazolimab plus EC group**  **(N = 249) n (%)** | | **Placebo plus EC group**  **(N = 247) n (%)** | |
| --- | --- | --- | --- | --- |
| Any immune related adverse events | Any grade^a^ | Grade ≥3 | Any grade^a^ | Grade ≥3 |
|  | 48 (19·3) | 15 (6·0) | 24 (9·7) | 2 (0·8) |
| Increased alanine aminotransferase | 7 (2·8) | 3 (1·2) | 7 (2·8) | 0 |
| Increased aspartate aminotransferase | 6 (2·4) | 2 (0·8) | 3 (1·2) | 0 |
| Increased γ-glutamyltransferase | 2 (0·8) | 0 | 0 | 0 |
| Elevated blood glucose | 2 (0·8) | 0 | 0 | 0 |
| Increased serum creatinine | 2 (0·8) | 0 | 0 | 0 |
| Increased blood lactate dehydrogenase | 2 (0·8) | 0 | 0 | 0 |
| Decreased platelet count | 2 (0·8) | 1 (0·4) | 0 | 0 |
| Decreased serum thyrotropin | 1 (0·4) | 0 | 2 (0·8) | 0 |
| Increased serum thyrotropin | 1 (0·4) | 0 | 2 (0·8) | 0 |
| Increased serum creatine phosphokinase | 1 (0·4) | 1 (0·4) | 0 | 0 |
| Increased serum glucose | 1 (0·4) | 0 | 2 (0·8) | 0 |
| Decreased neutrophil count | 1 (0·4) | 1 (0·4) | 0 | 0 |
| Hypothyroidism | 9 (3·6) | 0 | 2 (0·8) | 0 |
| Hyperthyroidism | 7 (2·8) | 0 | 1 (0·4) | 0 |
| Thyroid disease | 3 (1·2) | 0 | 0 | 0 |
| Immune-mediated lung disease | 5 (2·0) | 0 | 3 (1·2) | 2 (0·8) 2 |
| Pulmonary inflammation | 1 (0·4) | 1 (0·4) | 0 | 0 |
| Hyperglycemia | 2 (0·8) | 0 | 1 (0·4) | 0 |
| Hypochloremia | 1 (0·4) | 1 (0·4) | 0 | 0 |
| Hypercholesterolemia | 1 (0·4) | 1 (0·4) | 0 | 0 |
| Rash | 3 (1·2) | 0 | 2 (0·8) | 0 |
| Abnormal liver function | 2 (0·8) | 2 (0·8) | 0 | 0 |
| Immune-mediated hepatitis | 1 (0·4) | 1 (0·4) | 0 | 0 |
| Fever | 1 (0·4) | 1 (0·4) | 0 | 0 |
| Supraventricular extrasystole | 2 (0·8) | 0 | 0 | 0 |
| Myocarditis | 1 (0·4) | 1 (0·4) | 0 | 0 |
| Anemia | 3 (1·2) | 0 | 0 | 0 |
| Albuminuria | 1 (0·4) | 0 | 2 (0·8) | 0 |
| Acute pancreatitis | 1 (0·4) | 1 (0·4) |  |  |

1. Occurred in 2 or greater of patients in either group.

**Supplementary table 4: Non-author Collaborators**

*First name and last name are required and will appear in PubMed.

| Group Name(s): | | | | | |
| --- | --- | --- | --- | --- | --- |
| First Name and  Middle Initial(s) | Last Name | Academic  Degrees | Institution | Location (city,  state/province, country) | Role or Contribution,  eg, chair, principal  investigator |
| Xingxiang | Xu | MD | Jiangsu Subei People's Hospital | Yangzhou,China | Principal investigator |
| Shihong | Wei | MMed | Gansu Cancer Hospital | Lanzhou, China | Principal investigator |
| Lei | Yang | B.Med | Gansu Cancer Hospital | Lanzhou, China | Principal investigator |
| Yan | Yu | MD | Harbin Medical University Affiliated Tumor Hospital | Harbin, China | Principal investigator |
| Dan | Zhu | MMed | Jinhua Central Hospital | Jinhua, China | Principal investigator |
| Feng | Luo | MD | West China Hospital of Sichuan University | Chengdu, China | Principal investigator |
| Xuhong | Min | B.Med | Anhui Provincial Chest Hospital | Hefei, China | Principal investigator |
| Zhi | Xu | MD | The Second Affiliated Hospital of PLA Army Medical University | Chongqing , China | Principal investigator |
| Youlun | Ni | MD | The First Affiliated Hospital of Chongqing Medical University | Chongqing, China | Principal investigator |
| Yu | Yao | MD | The First Affiliated Hospital of Xi'an Jiaotong University | Xi'an , China | Principal investigator |
| Hui | Zhao | MD | The Second Affiliated Hospital of Anhui Medical University | Hefei, China | Principal investigator |
| Hong | Shen | MD | The Second Affiliated Hospital of Zhejiang University School of Medicine | Hangzhou , China | Principal investigator |
| Junzhen | Junzhen | MMed | The Affiliated Hospital of Inner Mongolia Medical University | Huhhot, China | Principal investigator |
| Qun | Hu | MD | The Affiliated Hospital of Inner Mongolia Medical University | Huhhot,, China | Principal investigator |
| Peng | Zhang | MD | Shanghai Pulmonary Hospital | Shanghai, China | Principal investigator |
| Xiaoling | Li | MD | Liaoning Cancer Hospital | Shenyang , China | Principal investigator |
| Ke | Hu | MD | Hubei Provincial People's Hospital | Wuhan , China | Principal investigator |
| Xianzhe | Yin | B.Med | Nanyang Second People's Hospital | Nanyang, China | Principal investigator |
| Kejing | Tang | MD，PhD | The First Affiliated Hospital of Sun Yat sen University | Guangzhou , China | Principal investigator |
| Daqing | Wang | B.Med | Hengshui People's Hospital | Hengshui, China | Principal investigator |
| Yuansong | Bai | MD, PhD | Jilin University Sino Japanese Friendship Hospital | Changchun , China | Principal investigator |
| Cailing | Jin | MMed | The First Affiliated Hospital of Xinxiang Medical College | Xinxiang, China | Principal investigator |
| Jianying | Zhou | MMed | The First Affiliated Hospital of Zhejiang University School of Medicine | Hangzhou , China | Principal investigator |
| Jun | Chen | MD | Tianjin Medical University General Hospital | Tianjin, China | Principal investigator |
| Aiqin | Liu | MMed | Shaanxi Provincial Cancer Hospital | Xi'an, China | Principal investigator |
| Yiping | Zhang | B.Med | Zhejiang Cancer Hospital | Hangzhou, China | Principal investigator |
| Liyun | Miao | MD | Gulou Hospital Affiliated to Nanjing University School of Medicine | Nanjing, China | Principal investigator |
| Xicheng | Wang | MMed | The First Affiliated Hospital of Guangdong Pharmaceutical University | Guangzhou, China | Principal investigator |
| Ningning | Zhou | B.Med | Sun Yat-sen University Cancer Center | Guangzhou, China | Principal investigator |
| Xiaofen | Wang | B.Med | Jieyang People's Hospital | Jieyang, China | Principal investigator |
| Shengqing | Li | MD | Fudan University Affiliated Huashan Hospital | Shanghai , China | Principal investigator |
| Guofang | Zhao | B.Med | Ningbo Second Hospital | Ningbo, China | Principal investigator |
| Xiaosheng | Hang | B.Med | Jiangnan University Affiliated Hospital (Wuxi Fourth People's Hospital) | Wuxi, China | Principal investigator |
